# Levistilide A promotes ferroptosis through the RNF40-HSP90α axis and inhibit colorectal cancer lung metastasis

**DOI:** 10.1038/s41420-026-03265-x

**Published:** 2026-08-15

**Authors:** Jia-Ming He, Chang-Shuo Li, Ying-Qian Liu, Qiang Wei, Chun-Qiao Zhou, Tian-Kuo Luan, Hao Deng, Hui-Min Wang, Jian-Hua Ran, Yong-Ping Ma, Di-Long Chen, Jing Li

**Affiliations:** 1 Department of Biochemistry and Molecular Biology, Basic Medical College, Molecular Medicine & Cancer Research Center, Chongqing Medical University, Chongqing, 400016 P.R. China; 2 Laboratory of Stem Cells and Tissue Engineering, Department of Histology and Embryology, Chongqing Medical University, Chongqing, 400016 P.R. China; 3 Department of Laboratory Medicine, The First Affiliated Hospital of Chongqing Medical University, Chongqing, 400016 P.R. China; 4 Department of Anatomy, Laboratory of Neuroscience and Tissue Engineering, Basic Medical College, Chongqing Medical University, Chongqing, 400016 P.R. China; 5 Chongqing Key Laboratory of Development and Utilization of Genuine Medicinal Materials in Three Gorges Reservoir Area, Chongqing Three Gorges Medical College, Chongqing, 404120 P.R. China; 6 NMPA Key Laboratory for Quality Monitoring of Narcotic Drugs and Psychotropic Substances, Chongqing Institute for Food and Drug Control, Chongqing, 401120 P.R. China

**Keywords:** Targeted therapies, Cell death

## Abstract

The molecular mechanisms underlying lung metastasis of colorectal cancer (CRC) remain largely elusive, and effective therapeutic agents are still lacking. In this study, we identify levistilide A (LeA) as a potential anti-metastatic agent against CRC lung metastasis. We demonstrate that heat shock protein 90α (HSP90α) is markedly upregulated in CRC and promotes lung metastasis by suppressing ferroptosis. Notably, combined treatment with LeA and the ferroptosis inducer RSL3 further alleviates lung metastatic burden in vivo. Mechanistically, we reveal that the E3 ubiquitin ligase ring finger protein 40 (RNF40) suppresses CRC cell proliferation by directly interacting with HSP90α, inducing ubiquitination at lysine 407 and promoting its proteasomal degradation. RNF40-mediated HSP90α downregulation leads to the accumulation of malondialdehyde (MDA) and reactive oxygen species (ROS), thereby inhibiting CRC cell growth. Collectively, our findings provide mechanistic insights into how LeA directly targets HSP90α or facilitates RNF40-HSP90α-mediated degradation of HSP90α to regulate ferroptosis in CRC.

**Figure:**
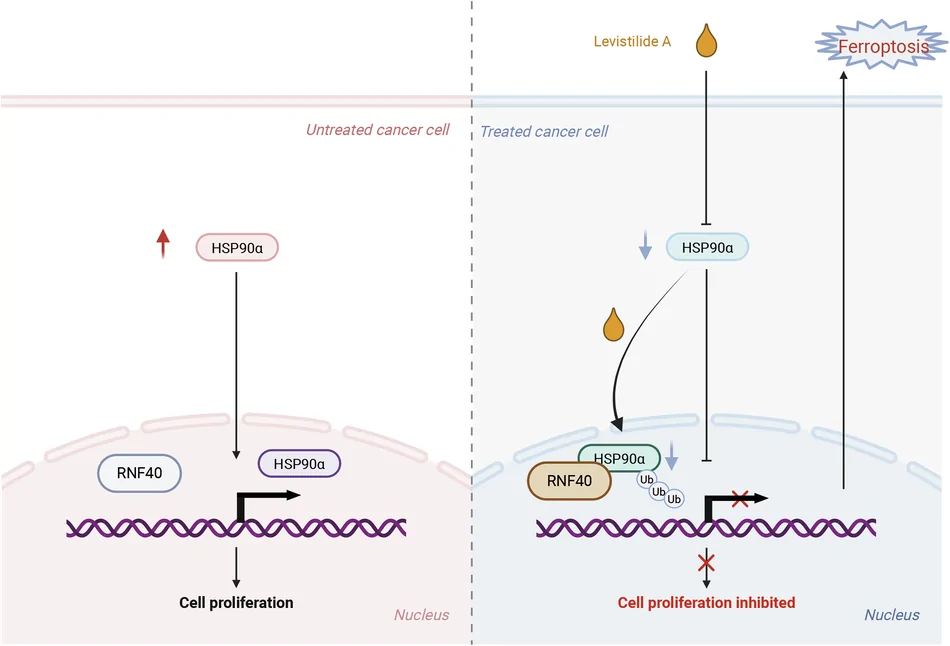

## Introduction

Colorectal cancer (CRC) ranks as the third most frequently diagnosed malignancy worldwide and imposes a substantial global health and socioeconomic burden [1, 2]. The overall 5-year survival rate of patients with CRC is approximately 60%; however, individual prognosis is largely determined by the occurrence of distant metastasis during disease progression [2]. CRC preferentially metastasizes to the liver and lungs, with the lung being one of the most common metastatic sites [3]. Currently, patients with metastatic CRC still face a dismal 5-year survival rate of only 15% [4]. For most patients with lung metastases, therapeutic options remain largely confined to systemic chemotherapy, with or without surgical resection [5]. Moreover, although immunotherapy is costly, it has demonstrated only limited clinical benefit in the majority of patients with CRC lung metastases and poses significant therapeutic challenges [6]. Therefore, there is an urgent unmet need to identify effective pharmacological strategies to alleviate CRC lung metastasis.

Natural products are chemical substances biosynthesized by plants, animals, insects, marine organisms, and microorganisms, and they often possess intrinsic pharmacological or biological activities [7, 8]. These compounds have made profound contributions to the treatment of numerous diseases, exemplified by artemisinin for malaria, simvastatin for hyperlipidemia, paclitaxel for cancer therapy, and rapamycin as an immunosuppressant [9–12]. High-throughput screening (HTS) represents a fundamental strategy for the rapid identification of novel small-molecule candidates and substantially accelerates drug discovery at the validation stage. Therefore, the establishment of appropriate high-throughput screening models is of critical importance for the efficient identification of antitumor natural products [13–15].

Ferroptosis is a distinct form of iron-dependent, non-apoptotic oxidative cell death characterized by the lethal accumulation of lipid peroxides caused by failure of the glutathione-dependent antioxidant defense system [16]. In general, metabolic enzymes responsible for the biosynthesis of oxidizable membrane lipids, such as acyl-CoA synthetase long-chain family member 4 (ACSL4) and lysophosphatidylcholine acyltransferase 3 (LPCAT3), as well as iron-dependent enzymes that oxidize membrane lipids, including arachidonic acid lipoxygenases (ALOXs), confer cellular susceptibility to ferroptosis [17–19]. In parallel, inhibition of other free radical-scavenging metabolites, including coenzyme Q10 (CoQ10) and tetrahydrobiopterin (BH4), can likewise sensitize cells to ferroptosis [20, 21]. Moreover, increased abundance of labile iron is sufficient to trigger ferroptotic cell death [22]. At present, multiple therapeutic agents that target ferroptosis have entered preclinical or clinical evaluation [23]. For example, Anomanolide C promotes GPX4 ubiquitination to induce autophagy-dependent ferroptosis, thereby suppressing the progression and metastasis of triple-negative breast cancer [24]. FerroTerminator-1 inhibits c-Myc-ACSL4-mediated ferroptosis, attenuates hepatic lipid accumulation, inflammation, and fibrosis, and exhibits therapeutic potential for patients with metabolic dysfunction-associated fatty liver disease (MAFLD) [25]. However, although several FDA-approved drugs, such as sorafenib, artemisinin, and gemcitabine, have shown promise in preclinical studies of ferroptosis induction, their therapeutic efficacy is often compromised by unfavorable off-target effects, resulting in a narrow therapeutic window and increased toxicity, which substantially limits their clinical application in ferroptosis-based therapies [26]. Therefore, the identification and development of safe and effective ferroptosis-targeted agents remain of paramount importance.

Heat shock protein 90α (HSP90α) is the most abundant member of the heat shock protein family, and its expression is markedly upregulated under cellular stress conditions. In mammals, approximately 600 cochaperones and client proteins have been identified to interact with HSP90α [27, 28]. Under physiological conditions, HSP90α maintains cellular homeostasis and functional integrity by forming multiprotein complexes with its cochaperones and client proteins [29]. Accumulating evidence indicates that HSP90α participates in the progression of multiple diseases by modulating key regulatory proteins involved in PCD, including receptor-interacting protein kinase 1 (RIP1) in necroptosis and Beclin-1 in apoptosis and autophagy [30, 31]. Accordingly, HSP90α and its cochaperone complexes have emerged as promising therapeutic targets [32–34]. Previous studies have demonstrated that HSP90α inhibitors represent potential antitumor agents, with compounds such as geldanamycin and 17-allylamino-17-demethoxygeldanamycin (17-AAG) having entered clinical trials [35, 36]. Collectively, these findings highlight the druggability and therapeutic feasibility of HSP90α as a pharmacological target.

In this study, we identify LeA as a potent ferroptosis inducer that effectively suppresses lung metastasis of CRC. We further validate HSP90α as the direct molecular target of LeA. Mechanistically, we demonstrate that HSP90α functions as a previously unrecognized ferroptosis suppressor, and that LeA promotes the interaction between RNF40 and HSP90α, thereby facilitating HSP90α degradation. In addition, combined treatment with LeA and the ferroptosis inducer RSL3 markedly inhibits CRC lung metastasis. Collectively, these findings uncover a novel ferroptosis suppressor and establish a promising therapeutic strategy for the treatment of metastatic CRC through targeted modulation of ferroptosis.

## Results

### Levistilide A inhibits colorectal cancer metastasis

**Fig. 1:**
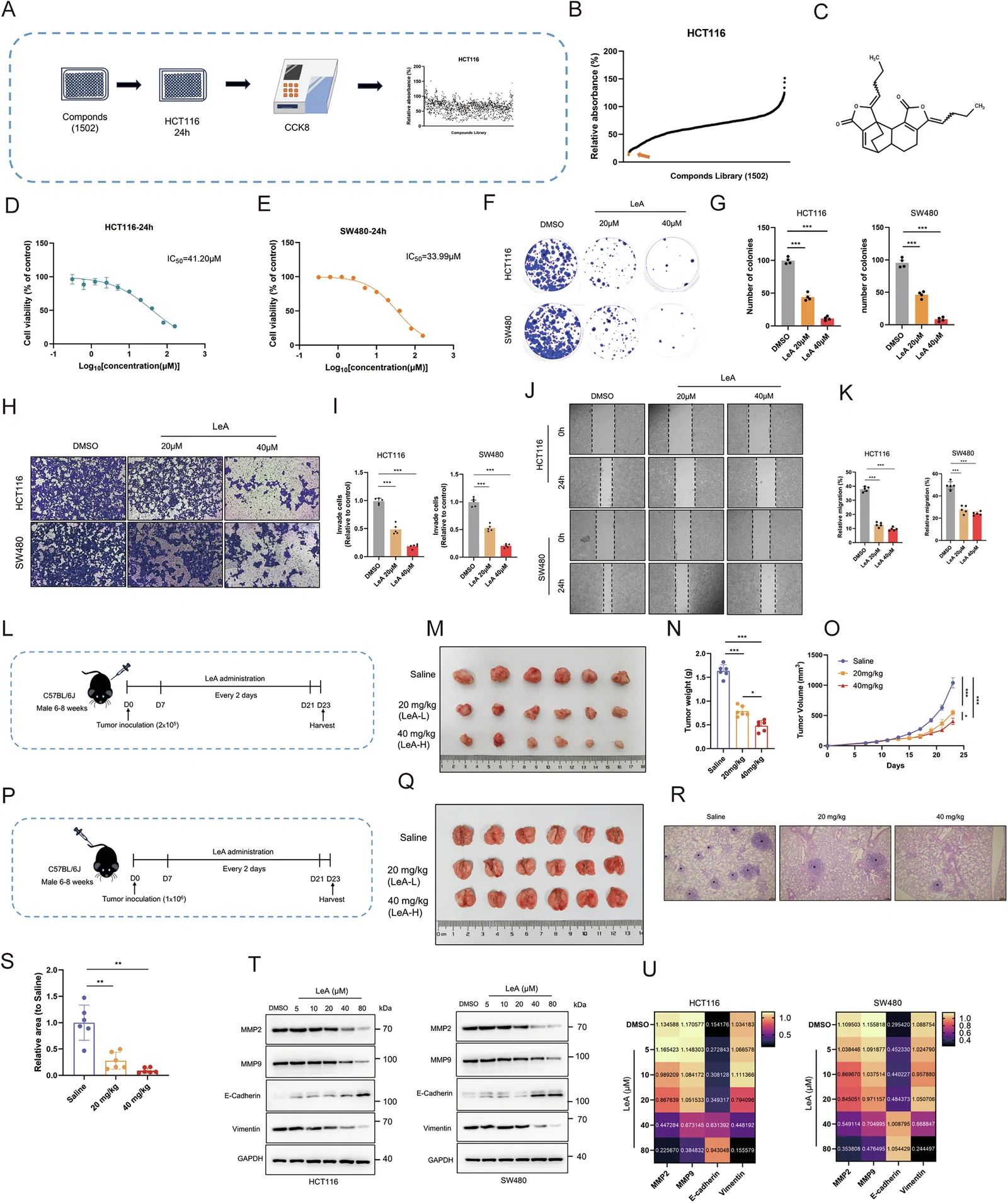
LeA inhibits colorectal cancer lung metastasis. **A** Schematic of the high-throughput screening. HCT116 cells were treated with 1502 small-molecule compounds in a 96-well plate for 24 h. Cell viability was subsequently measured, and the results were statistically analyzed. **B** Ranking of the effects of different compounds from the library on HCT116 cell viability. The lower-left corner highlights the top-ranked compounds that most effectively inhibited colorectal cancer cell growth. **C** Chemical structure of Levistilide A. **D**, **E** CCK-8 assay was performed to determine the half-maximal inhibitory concentration (IC₅₀) of LeA in HCT116 and SW480 cells after 24-h treatment with various concentrations of the compound. **F**, **G** Colony formation assays were performed in 12-well plates. HCT116 and SW480 cells were treated with DMSO or Levistilide A (LeA) at 20 or 40 μM for 24 h, after which the drug was removed and cells were cultured for 7 days. Representative images are shown on the left, and quantitative analysis on the right. Experiments were independently repeated three times. **H**, **I** Transwell assays were performed with HCT116 and SW480 cells treated with DMSO or LeA at 20 or 40 μM for 24 h. After drug removal, cells were cultured for an additional 2 days. Representative images were captured at ×10 magnification (left), and quantification is shown on the right. Experiments were independently repeated three times. **J**, **K** Wound healing assays were performed on HCT116 and SW480 cells treated with DMSO or Levistilide A (LeA) at 20 or 40 μM for 24 h. After drug removal, cells were cultured for an additional 2 days. Representative images were captured at ×10 magnification (left), with quantification shown on the right. Experiments were independently repeated three times. **L** Schematic diagram of the mouse subcutaneous tumor formation assay. **M** Representative images of tumors collected at the end of the mouse subcutaneous xenograft experiment are shown, including the control group (saline) and groups treated with different concentrations of Levistilide A (LeA-L: 20 mg/kg; LeA-H: 40 mg/kg). **N** Tumor weight measurements for each group. **O** Tumor growth curves for each treatment group. **P** Schematic diagram of the mouse colorectal cancer lung metastasis model experimental workflow. **Q** Representative images of lungs from the mouse colorectal cancer lung metastasis model at the end of the experiment, showing the control group (saline) and treatment groups with different LeA doses (LeA-L: 20 mg/kg; LeA-H: 40 mg/kg). **R** HE staining showing lung tissue architecture and metastatic tumor structures (Scale bar: 100 μm). **S** Quantitative analysis showing the metastatic lung area across different treatment groups. **T** Protein expression analysis in HCT116 and SW480 cells following 24-h treatment with DMSO or various concentrations of LeA (5, 10, 20, 40, 80 μM). Experiments were independently repeated three times. (U) Heatmap showing the relative protein expression under different treatments, with the y-axis representing the treatment conditions and the x-axis representing the individual proteins.

To identify novel food-derived compounds with potent anti–colorectal cancer activity, we screened a compound library containing 1502 small molecules. Cells were treated with each compound at a concentration of 25 μM for 24 h, and compounds exhibiting an inhibition rate greater than 80% were selected for further investigation (Fig. 1A, B). Among the candidate compounds, we focused on LeA (Fig. 1C), which has previously been reported to exert anti-tumor effects only in breast cancer cells by inducing reactive oxygen species (ROS) accumulation [37]. Cell viability assays demonstrated that LeA markedly inhibited the proliferation of colorectal cancer cells in a dose- and time-dependent manner (Fig. 1D–G; Fig. S1A–S1J). In addition, LeA significantly suppressed the migratory and invasive capacities of colorectal cancer cells, as assessed by wound-healing and transwell assays (Fig. 1H–K). To further evaluate the anti-tumor efficacy of LeA in vivo, we employed murine colorectal cancer models. In the subcutaneous xenograft model, LeA treatment significantly reduced tumor growth compared with control mice (Fig. 1L–O). Consistently, in the lung metastasis model, LeA markedly decreased the number and burden of metastatic nodules in the lungs (Fig. 1P–S). Hematoxylin and eosin (HE) staining of major organs revealed no obvious histopathological abnormalities, indicating that LeA did not induce overt organ toxicity (Fig. S1K–S1L). Moreover, analysis of proteins associated with cell invasion and epithelial–mesenchymal transition (EMT) further confirmed that LeA effectively attenuated the invasive potential of colorectal cancer cells at the molecular level (Fig. 1T, U). Collectively, these findings suggest that LeA represents a promising candidate for the development of therapeutic strategies targeting lung metastasis in colorectal cancer.

### Levistilide A inhibits colorectal cancer through ferroptosis

**Fig. 2:**
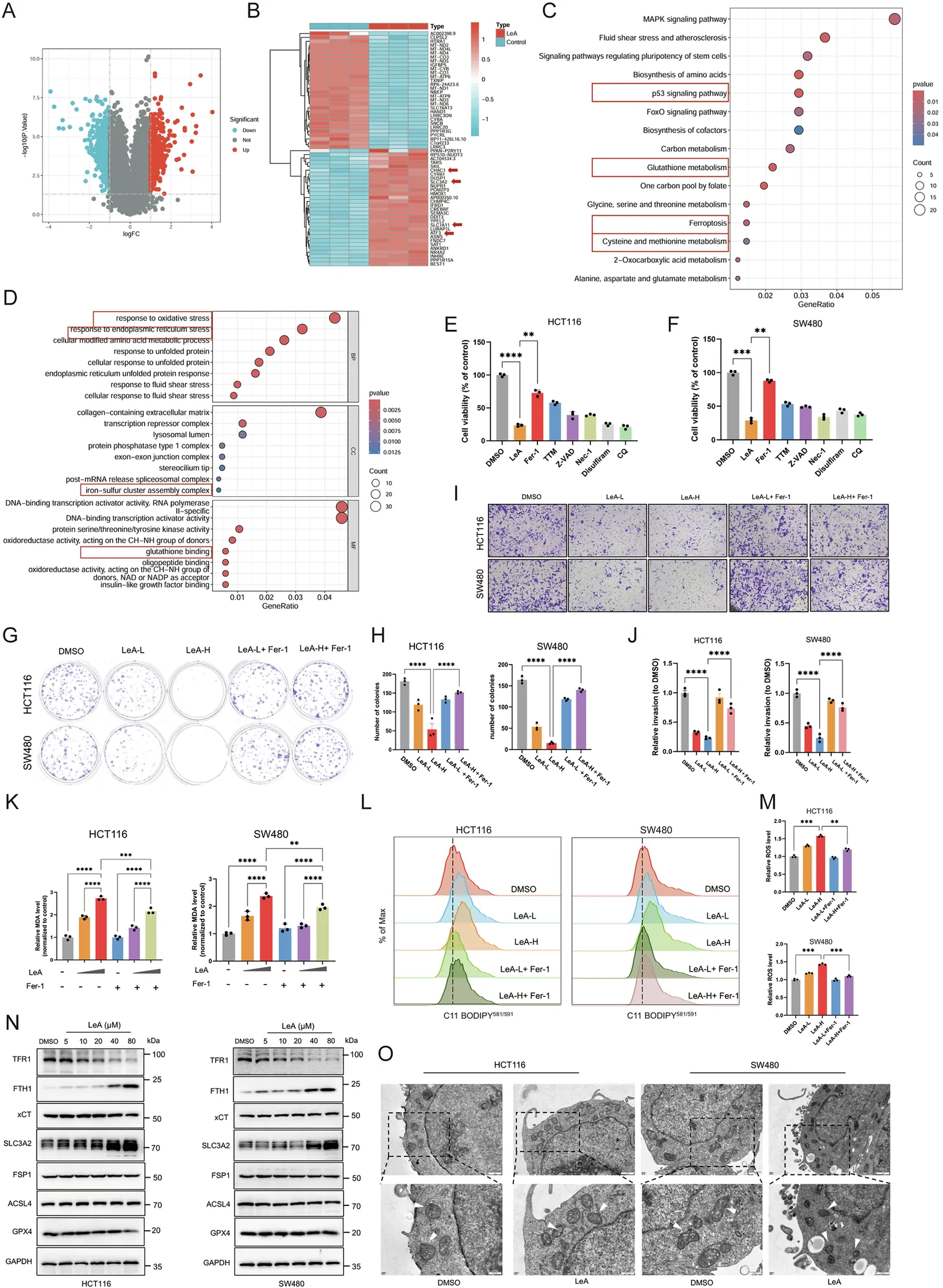
LeA induces ferroptosis in colorectal cancer cells. **A**, **B** HCT116 cells were treated with 40 μM LeA for 12 h, followed by RNA extraction for sequencing. Differentially expressed genes were analyzed using the criteria |log₂ fold change|≥ 1 and *P* < 0.05, which were used to generate the volcano plot and heatmap. **C** KEGG pathway analysis showing the top 15 enriched pathways with *P* < 0.05. **D** GO analysis showing the top 8 enriched pathways with *P* < 0.05. **E**, **F** Cells were pretreated with various programmed cell death inhibitors at different concentrations for 1 h, followed by treatment with 40 μM LeA for 24 h. The inhibitors used were: 5 μM Necrostatin-1s, 10 μM Z-VAD-fmk, 10 μM ferrostatin-1, 10 μM TTM, 5 μM Disulfiram, 20 μM chloroquine (CQ), or DMSO as control. **G**, **H** The colony formation assay was performed with the following treatment groups: DMSO, LeA-L (20 μM), LeA-H (40 μM), LeA-L (20 μM) plus Fer-1 (5 μM), and LeA-H (40 μM) plus Fer-1 (5 μM). Fer-1 was added 1 h prior to LeA treatment. After 7 days, colonies were counted and quantified. **I**,**J** The Transwell assay was performed with the following treatment groups: DMSO, LeA-L (20 μM), LeA-H (40 μM), LeA-L (20 μM) plus Fer-1 (5 μM), and LeA-H (40 μM) plus Fer-1 (5 μM). Fer-1 was added 1 h prior to LeA treatment. After 24 h, cells were stained and quantified. **K** The MDA assay was performed with the following treatment groups: DMSO, LeA-L (20 μM), LeA-H (40 μM), LeA-L (20 μM) plus Fer-1 (5 μM), and LeA-H (40 μM) plus Fer-1 (5 μM). Fer-1 was added 1 h prior to LeA treatment. After 24 h, MDA levels were measured and quantified. **L**, **M** The ROS assay was conducted with the following treatment groups: DMSO, LeA-L (20 μM), LeA-H (40 μM), LeA-L (20 μM) plus Fer-1 (5 μM), and LeA-H (40 μM) plus Fer-1 (5 μM). After 12 h of treatment, cells were incubated with 5 μM BODIPY 581/591 C11 probe for 30 min, collected, and analyzed by flow cytometry. **N** Protein expression analysis in HCT116 and SW480 cells following 24-h treatment with DMSO or various concentrations of LeA (5, 10, 20, 40, 80 μM). Experiments were independently repeated three times. **O** Cells were treated with DMSO or the drug at 40 μM for 12 h, and then collected. TEM images showing the ultrastructure of tumor cells, with white arrows indicating mitochondria. The scale bar for the upper image is 1 μm, and for the lower image is 500 nm.

To elucidate the potential mechanism underlying the anti-tumor effects of LeA, we performed transcriptomic sequencing analysis. Differentially expressed genes were significantly enriched in multiple signaling pathways associated with ferroptosis (Fig. 2A–D; Fig. S2A). To further determine the specific mode of programmed cell death induced by LeA, a panel of pharmacological inhibitors targeting distinct cell death pathways was applied. Among these inhibitors, the ferroptosis inhibitor ferrostatin-1 (Fer-1) most effectively rescued LeA–induced cell death (Fig. 2E, F). In parallel, we conducted a secondary screening of the initial compound library by co-treatment with Fer-1. Consistently, LeA was again identified as a top candidate whose cytotoxic effects were significantly reversed by ferroptosis inhibition (Fig. S2B). Functional assays further demonstrated that Fer-1 markedly attenuated LeA–induced suppression of colorectal cancer cell proliferation, migration, and invasion (Fig. 2G–J). Moreover, the elevation of intracellular reactive oxygen species (ROS) and malondialdehyde (MDA) levels triggered by LeA was substantially reduced upon Fer-1 treatment (Fig. 2K–M). To further characterize the ferroptotic phenotype, we measured the GSH/GSSG ratio and intracellular Fe²⁺ levels. LeA treatment significantly reduced the GSH/GSSG ratio (Fig. S2C) and increased labile iron pool (Fig. S2D) in both HCT116 and SW480 cells. Co-treatment with the ferroptosis inhibitor Fer-1 effectively reversed these changes, further supporting that LeA induces ferroptosis through disruption of redox and iron homeostasis. However, analysis of canonical ferroptosis-related proteins revealed no significant changes in the expression levels of GPX4, xCT (SLC7A11), FSP1, or ACSL4. In addition, alterations in iron metabolism–related markers were indicative of a cellular state resistant to classical ferroptosis (Fig. 2N; Fig. S2E–F). These findings suggest that LeA does not engage the canonical ferroptosis regulatory machinery and instead may act through a previously uncharacterized ferroptosis-associated pathway, implicating its molecular target as a potential novel ferroptosis regulator. Consistent with this hypothesis, transmission electron microscopy revealed that LeA treatment induced typical ferroptotic mitochondrial ultrastructural features, including mitochondrial shrinkage and increased membrane density, in colorectal cancer cells (Fig. 2O). Collectively, these results indicate that LeA exerts its anti-tumor effects through a non-canonical ferroptosis-like mechanism, likely mediated by its direct molecular target.

### Levistilide A targets heat shock protein 90α

**Fig. 3:**
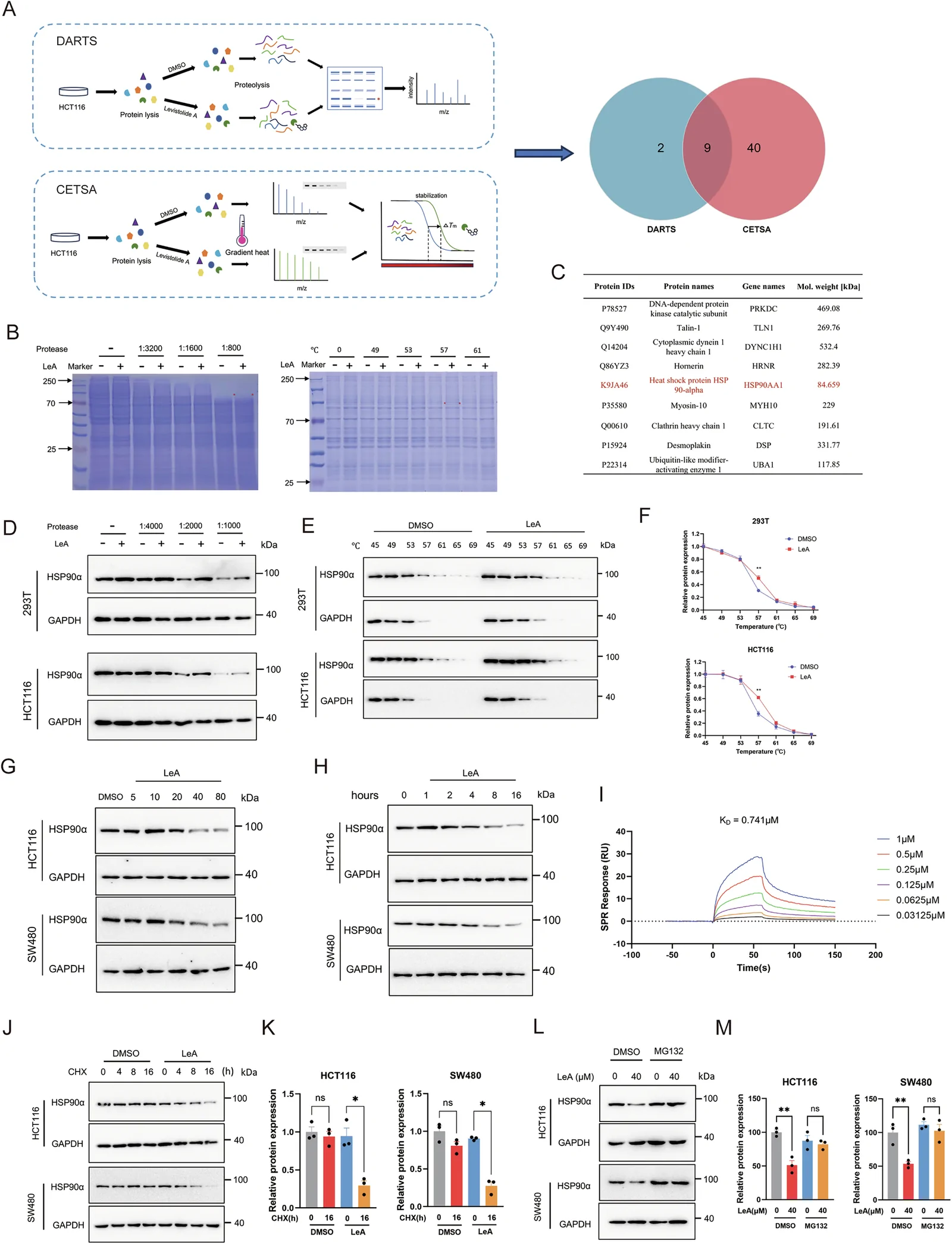
LeA targets HSP90α by promoting its proteasomal degradation and reducing its protein stability. **A** Schematic illustration showing the workflow for identifying drug target proteins using DARTS and CETSA, with a Venn diagram depicting the intersection process; nine proteins were found in the overlap. **B** Coomassie Brilliant Blue staining showing the regions of the PAGE gel excised for mass spectrometry analysis. Red stars indicate the positions of the gel slices. **C** Detailed protein information obtained from the intersection of DARTS and CETSA mass spectrometry results. Proteins highlighted in red indicate the identified target protein. **D** DARTS assay performed after treatment with varying concentrations of protease (1:4000, 1:2000, 1:1000) or LeA (200 μM), followed by verification via Western blot. **E**, **F** Cell lysates were incubated with LeA (200 μM) for 1 h and then subjected to heat treatment at various temperatures for 3 min. Protein stability was assessed by Western blot and quantified. **G** HCT116 and SW480 cells were treated with DMSO or varying concentrations of LeA (5, 10, 20, 40, 80 μM) for 12 h, followed by cell lysis and Western blot analysis. **H** HCT116 and SW480 cells were treated with DMSO or 40 μM LeA for 1, 2, 4, 8, or 16 h, followed by cell lysis and Western blot analysis. **I** SPR analysis was performed to assess the binding affinity between the LeA and HSP90α, using six different drug concentrations. (**J**,**K**) HCT116 and SW480 cells were treated with cycloheximide (CHX, 100 µg/mL) in combination with LeA (40 µM) for 0, 4, 8, and 16 h. Cells were then lysed, and protein levels were analyzed by WB with subsequent quantification. **L**, **M** HCT116 and SW480 cells were treated with LeA (40 µM) for 12 h, with MG132 (20 µM) added during the first 6 h prior to cell lysis. Protein levels were analyzed by WB and subsequently quantified.

To identify the direct molecular target of LeA, we integrated DARTS–LC/MS and CETSA–LC/MS–based target identification strategies (Fig. 3A). Proteins with confidence scores greater than 200 from both mass spectrometry datasets were intersected, and combined with the molecular weight information of excised gel bands from DARTS analysis, HSP90α was prioritized as a putative target of LeA (Fig. 3B, C). Subsequent validation by DARTS demonstrated that LeA markedly protected HSP90α from proteolytic degradation, which was detectable even at a dilution of 1:2000 (Fig. 3D). Consistently, CETSA validation revealed that LeA significantly enhanced the thermal stability of HSP90α at 57 °C (Fig. 3E, F), further supporting a direct interaction between the compound and the target protein. Interestingly, despite stabilizing HSP90α in DARTS and CETSA assays, LeA treatment led to a pronounced downregulation of HSP90α protein levels in colorectal cancer cells in both dose- and time-dependent manners (Fig. 3G, H). To directly characterize their interaction, recombinant HSP90α protein was purified and subjected to surface plasmon resonance (SPR) analysis. SPR measurements confirmed a strong binding affinity between LeA and HSP90α (Fig. 3I), with an association rate constant (k_on) of 8.42 × 10³ M⁻¹ s⁻¹, a dissociation rate constant (k_off) of 6.24 × 10⁻³ s⁻¹, and an equilibrium dissociation constant (K_D) of 7.41 × 10⁻⁷ M, indicative of a rapid association and slow dissociation binding mode. Molecular dynamics simulations further supported the stability of the LeA–HSP90α complex (Fig. S3A–C).

To elucidate the mechanism underlying HSP90α downregulation, we first assessed transcriptional regulation and observed no significant changes in HSP90AA1 mRNA expression following LeA treatment (Fig. S3D). In contrast, cycloheximide chase assays revealed a markedly shortened half-life of HSP90α protein upon LeA exposure (Fig. 3J, K). Moreover, the proteasome inhibitor MG132 effectively rescued LeA–induced HSP90α degradation (Fig. 3L, M), indicating that LeA reduces HSP90α levels through a proteasome-dependent protein degradation mechanism. Consistent with a role for HSP90α in ferroptosis regulation, analysis of the CTRP database showed that HSP90AA1 expression positively correlates with cellular sensitivity to multiple ferroptosis inducers (Fig. S3E). In contrast, data from the DepMap database revealed no significant correlation between HSP90AA1 expression and canonical ferroptosis suppressor genes (Fig. S3F). Functionally, knockdown of HSP90AA1 significantly enhanced the sensitivity of colorectal cancer cells to the ferroptosis inducer RSL3 (Fig. S3G, H). Collectively, these findings identify LeA as a novel HSP90α inhibitor that acts by destabilizing HSP90α protein rather than suppressing its transcription. Importantly, targeting HSP90α emerges as a previously underappreciated strategy to potentiate ferroptosis, providing mechanistic insight into the ferroptosis-inducing activity of LeA.

### Levistilide A promotes the interaction between RNF40 and HSP90α

**Fig. 4:**
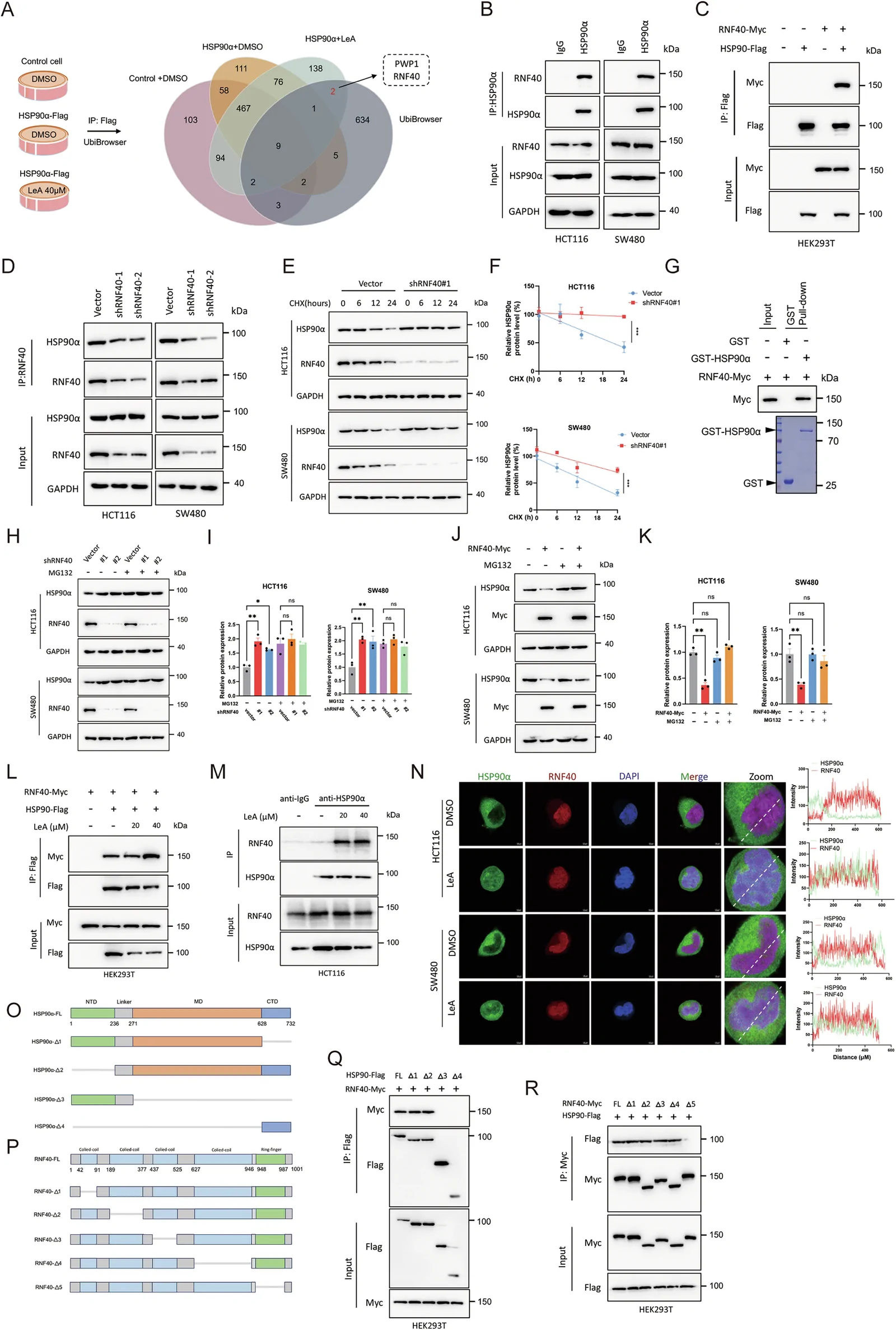
LeA enhances the interaction between RNF40 and HSP90α. **A** 293 T cells were divided into the following groups: DMSO treatment, DMSO treatment combined with HSP90α-Flag transfection, and LeA (40 µM) treatment combined with HSP90α-Flag transfection. UbiBrowser was used to intersect ubiquitination-related proteins. Proteins highlighted in red represent those whose interaction with HSP90α-Flag was enhanced by LeA, with PWP1 and RNF40 specifically annotated within the dashed box. **B** Endogenous co-IP assays were performed in HCT116 and SW480 cells, and the results were validated by WB. **C** Exogenous co-IP assays were performed in HEK293T cells. RNF40-Myc and HSP90α-Flag were transfected in the respective groups, and the interactions were validated by WB. **D** Endogenous co-immunoprecipitation (IP) assays were performed in HCT116 and SW480 cells with stable RNF40 knockdown, and the interactions were validated by WB. **E**, **F** HCT116 and SW480 cells with stable RNF40 knockdown were treated with 50 μg/mL CHX for different time points (0, 6, 12, and 24 h). After treatment, protein levels were analyzed by WB and quantified. **G** GST and GST-HSP90α proteins were separately purified from *E. coli*. Then, 50 μg of GST or 50 μg of GST-HSP90α was incubated with HEK293T cell lysates at 4 °C. After washing, the bound proteins were eluted, boiled in 2× SDS loading buffer, and analyzed by WB. **H**,**I** In stable RNF40 knockdown HCT116 and SW480 cells, MG132 (20 μM) was added 6 h prior to sample collection. Cells were then lysed, and protein levels were analyzed by WB. The experiment was independently repeated three times, and quantitative analysis was performed. **J**, **K** In HCT116 and SW480 cells stably overexpressing RNF40-Myc, MG132 (20 μM) was added 6 h prior to sample collection. Cells were then lysed, and protein levels were analyzed by WB. The experiment was independently repeated three times, and quantitative analysis was performed. **L** In HEK293T cells, exogenous RNF40-Myc or HSP90α-Flag was transfected, followed by treatment with LeA at 20 μM or 40 μM. IP assays were then performed, and the interactions were analyzed by WB. **M** Endogenous IP was performed in HCT116 cells treated with 20 μM or 40 μM LeA, followed by WB analysis to assess protein interactions. **N** IF staining was performed in HCT116 and SW480 cells treated with DMSO or 40 μM LeA for 8 h, followed by imaging with a confocal microscope. Quantification of fluorescence intensity within the dashed regions was performed using ImageJ software. Scale bar: 100 μm. **O**, **P** Schematic diagrams of the protein domains of HSP90α and RNF40. **Q** Co-transfection of full-length RNF40-Myc with various truncated HSP90α-Flag constructs was performed. After 36 h, IP was carried out, followed by WB analysis. **R** Co-transfection of full-length HSP90α-Flag with various truncated RNF40-Myc constructs was performed. After 36 h, IP was conducted, followed by WB analysis.

HSP90α is a well-characterized molecular chaperone whose functions critically depend on interactions with a wide range of co-chaperones and client proteins. To identify the specific factor(s) responsible for LeA–induced HSP90α destabilization, we performed immunoprecipitation coupled with mass spectrometry (IP-MS) to profile HSP90α-interacting proteins under LeA treatment. Given that protein degradation primarily occurs through either the lysosomal pathway or the ubiquitin–proteasome system, and that proteasome inhibition by MG132 markedly rescued LeA–induced HSP90α degradation (Fig. 3L, M), we focused our analysis on ubiquitin-related regulators. By integrating IP-MS candidates with data from the ubiquitination database UbiBrowser (http://ubbrowser.bio-it.cn), we identified two ubiquitin-associated proteins, RNF40 and PWP1, as potential mediators of HSP90α regulation (Fig. 4A). RNF40 is a RING-type E3 ubiquitin ligase known to regulate histone modifications, DNA damage responses, and tumor progression and metastasis [38, 39]. Periodic tryptophan protein 1 (PWP1) contains WD40 repeat domains and plays essential roles in ribosome biogenesis and regulatory protein networks [40, 41]. Notably, previous studies have reported a physical interaction between RNF40 and HSP90α [42]. Consistently, analysis of the TCGA dataset revealed a significant positive correlation between RNF40 and HSP90AA1 expression levels (Fig. S4A). To validate this finding in independent clinical cohorts, we analyzed two CRC datasets from the GEO database. As shown in Fig. S4B-C, a significant positive correlation between RNF40 and HSP90AA1 expression was also observed in both validation sets (GSE21815: *R* = 0.5771, *P* < 0.001; GSE31905: R = 0.3485, *P* < 0.0039), confirming the robustness of the TCGA observation.

To validate the IP-MS findings, we performed co-immunoprecipitation (co-IP) assays. Overexpression of Flag-tagged HSP90α demonstrated that LeA markedly enhanced the interaction between RNF40 and HSP90α (Fig. 4B, C). In contrast, RNF40 knockdown reduced both HSP90α protein levels and the interaction between the two proteins (Fig. 4D, Fig. S4B). Functionally, RNF40 depletion significantly prolonged the half-life of HSP90α, whereas RNF40 overexpression produced the opposite effect (Fig. 4E, F; Fig. S4C). To determine whether RNF40 and HSP90α interact directly, we conducted in vitro GST pull-down assays using purified proteins. These experiments confirmed a direct physical interaction between RNF40 and HSP90α (Fig. 4G). Moreover, under proteasome-inhibited conditions (MG132 treatment), RNF40 knockdown enhanced HSP90α stability, while RNF40 overexpression accelerated HSP90α degradation (Fig. 4H–K), further supporting RNF40 as a critical determinant of HSP90α proteostasis.

Consistent with these findings, LeA treatment resulted in a marked reduction of HSP90α protein levels relative to controls. Exogenous co-IP experiments revealed that treatment with 40 μM LeA significantly strengthened the association between RNF40 and HSP90α (Fig. 4L), which was further confirmed by endogenous co-IP assays (Fig. 4M). Immunofluorescence analysis demonstrated that LeA promoted nuclear translocation of HSP90α and increased its spatial colocalization with RNF40, thereby facilitating ubiquitination-dependent regulation (Fig. 4N).

Collectively, these results indicate that LeA enhances RNF40-HSP90α association. This enhancement is reminiscent of a molecular glue-like mechanism, although definitive classification awaits structural validation [43]. To further define the structural basis of this interaction, we generated a series of truncation mutants of RNF40 and HSP90α based on their known domain architectures [39, 44]. HSP90α consists of an N-terminal ATP-binding domain, a middle client-binding domain, and a C-terminal dimerization domain (Fig. 4O), whereas RNF40 is composed of four helical domains and a C-terminal RING-finger domain (Fig. 4P). Co-IP assays revealed that deletion of the middle domain of HSP90α or the C-terminal RING-finger domain of RNF40 abolished their interaction (Fig. 4Q, R). These findings demonstrate that the HSP90α middle domain and the RNF40 RING-finger domain are essential for complex formation. Taken together, our data establish that Levistilide A promotes RNF40–HSP90α interaction, thereby facilitating RNF40-dependent ubiquitination and proteasomal degradation of HSP90α.

To further investigate whether LeA directly facilitates the RNF40-HSP90α interaction, we performed surface plasmon resonance (SPR) analysis in two configurations. First, we immobilized RNF40 on the sensor chip and measured its direct binding affinity for LeA, yielding a Kd of 0.853 μM (Fig. S4D), which is comparable to the affinity between LeA and HSP90α (Kd = 0.74 μM, Fig. 3I). Second, we pre-incubated LeA with RNF40 and injected the complex over immobilized HSP90α. Remarkably, the pre-formed RNF40-LeA complex bound to HSP90α with a Kd of 0.29 μM (Fig. S4E), representing an approximately 2.5- to 3-fold higher affinity compared to either binary interaction alone. This cooperative binding behavior is characteristic of a molecular glue-like mechanism, wherein LeA simultaneously engages both RNF40 and HSP90α to stabilize their interaction. Although a co-crystal structure of the ternary complex would be required for definitive classification, these biophysical data provide orthogonal evidence supporting the model that LeA promotes RNF40-HSP90α complex formation.

### RNF40 catalyzes K48-linked ubiquitination of HSP90α at K407

**Fig. 5:**
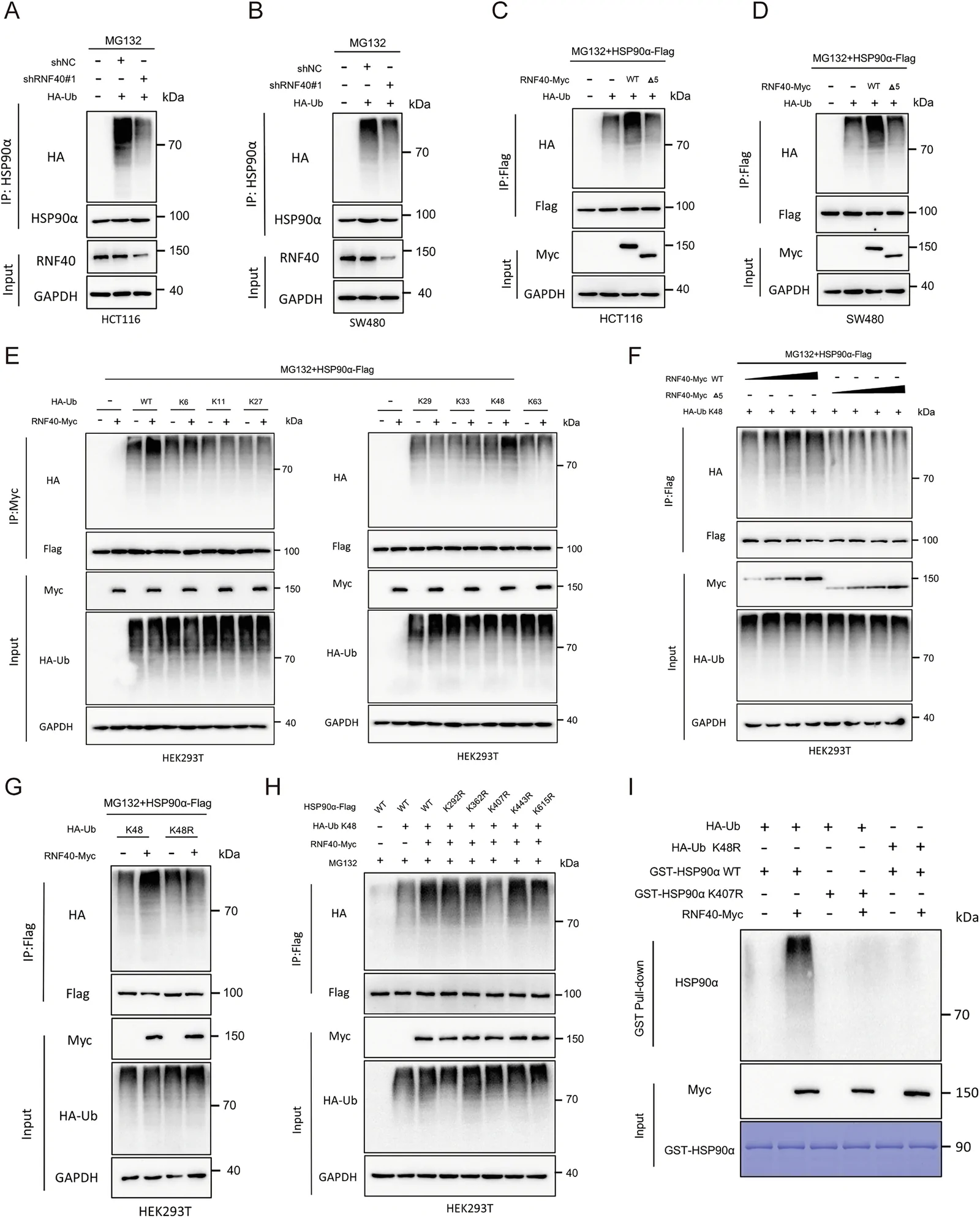
RNF40 regulates HSP90α at K407 via K48-linked ubiquitination. **A**, **B** In HCT116 and SW480 cells, HA-Ub was co-transfected with shNC or shRNF40 plasmids for 36 h. Cells were treated with MG132 (20 µM) for 6 h prior to collection, followed by IP to assess ubiquitination levels. **C**, **D** In HCT116 and SW480 cells, HA-Ub, HSP90α-Flag, and either wild-type RNF40-Myc or RNF40-Myc mutant plasmids were co-transfected for 36 h. Cells were treated with MG132 (20 µM) for 6 h prior to collection, followed by IP to assess ubiquitination levels. **E** In HEK293T cells, HSP90α-Flag, RNF40-Myc, and HA-Ub plasmids carrying different mutation sites were co-transfected for 36 h. Cells were treated with MG132 (20 µM) for 6 h prior to collection, followed by IP to assess ubiquitination levels. **F** In HEK293T cells, HSP90α-Flag, RNF40-Myc, truncated RNF40-Myc plasmids, or HA-Ub K48 mutant plasmids were co-transfected for 36 h. Cells were treated with MG132 (20 µM) for 6 h prior to collection, followed by IP to assess ubiquitination levels. **G** In HEK293T cells, HSP90α-Flag, RNF40-Myc, HA-Ub, or HA-Ub K48 mutant plasmids were co-transfected for 36 h. Six hours prior to collection, cells were treated with MG132 (20 µM), followed by immunoprecipitation to assess ubiquitination levels. **H** In HEK293T cells, RNF40-Myc, HA-Ub K48, HSP90α-Flag, or HSP90α-Flag plasmids carrying different point mutations were co-transfected for 36 h. Six hours prior to collection, cells were treated with MG132 (20 µM), followed by immunoprecipitation to assess ubiquitination levels. **I** GST-HSP90α and GST-HSP90α K407 proteins were separately purified from prokaryotic E. coli. Each 50 µg of purified protein was incubated at 4 °C with HEK293T crude lysates transfected with HA-Ub, HA-Ub K48R, or RNF40-Myc. After incubation, proteins were eluted, mixed with 2× SDS loading buffer, boiled, and subjected to WB analysis.

To further determine whether RNF40 downregulates HSP90α through ubiquitin-mediated degradation, we next examined the ubiquitination status of HSP90α upon modulation of RNF40 expression. Consistent with our hypothesis, RNF40 knockdown markedly reduced HSP90α ubiquitination in both HCT116 and SW480 cells (Fig. 5A, B). Conversely, RNF40 overexpression significantly increased HSP90α ubiquitination in these cells (Fig. 5C, D). Previous studies have demonstrated that the ubiquitin ligase activity of RNF40 is mediated by its C-terminal RING-finger domain (reference). In line with this notion, overexpression of wild-type RNF40 robustly enhanced HSP90α ubiquitination, whereas an RNF40 mutant lacking the RING-finger domain (RNF40 Δ5) failed to do so (Fig. 5C, D). These observations were further corroborated by in vitro ubiquitination assays, confirming the requirement of the RING-finger domain for RNF40-mediated HSP90α ubiquitination (Fig. S5A). To identify the specific ubiquitin linkage type catalyzed by RNF40, we performed co-transfection experiments with ubiquitin mutants harboring distinct lysine linkages. We found that RNF40 induced K48-linked polyubiquitination of HSP90α in a dose-dependent manner (Fig. 5E, F), consistent with a proteasome-dependent degradation pathway. In contrast, expression of a ubiquitin mutant in which lysine 48 was replaced by arginine (K48R) failed to enhance HSP90α ubiquitination (Fig. 5G), further confirming the specificity of K48-linked ubiquitin chains. To pinpoint the RNF40-mediated ubiquitination site(s) on HSP90α, we used PhosphoSitePlus (https://www.phosphosite.org) to predict potential ubiquitinated lysine residues, identifying K292, K362, K407, K443, and K615 as candidate sites (Figure S5B). Based on these predictions, we generated a series of HSP90α lysine-to-arginine mutants and co-expressed them with RNF40-Myc. Notably, RNF40 failed to promote K48-linked polyubiquitination of the HSP90α K407R mutant (Fig. 5H), a finding that was further validated by in vitro ubiquitination assays (Fig. 5I).

Given that HSP90α functions as a highly conserved molecular chaperone, we next assessed the evolutionary conservation of the identified site. Sequence alignment analysis revealed that lysine 407 is highly conserved across species (Fig. S5C). Consistently, the HSP90α K407R mutant exhibited a significantly prolonged protein half-life compared with wild-type HSP90α (Fig. S5D, E), supporting a critical role for this residue in regulating HSP90α stability. Collectively, these data demonstrate that RNF40 specifically catalyzes K48-linked polyubiquitination of HSP90α at lysine 407, thereby promoting its proteasomal degradation.

### Levistilide A inhibits the metastasis of colorectal cancer cells by promoting RNF40-HSP90 interaction

**Fig. 6:**
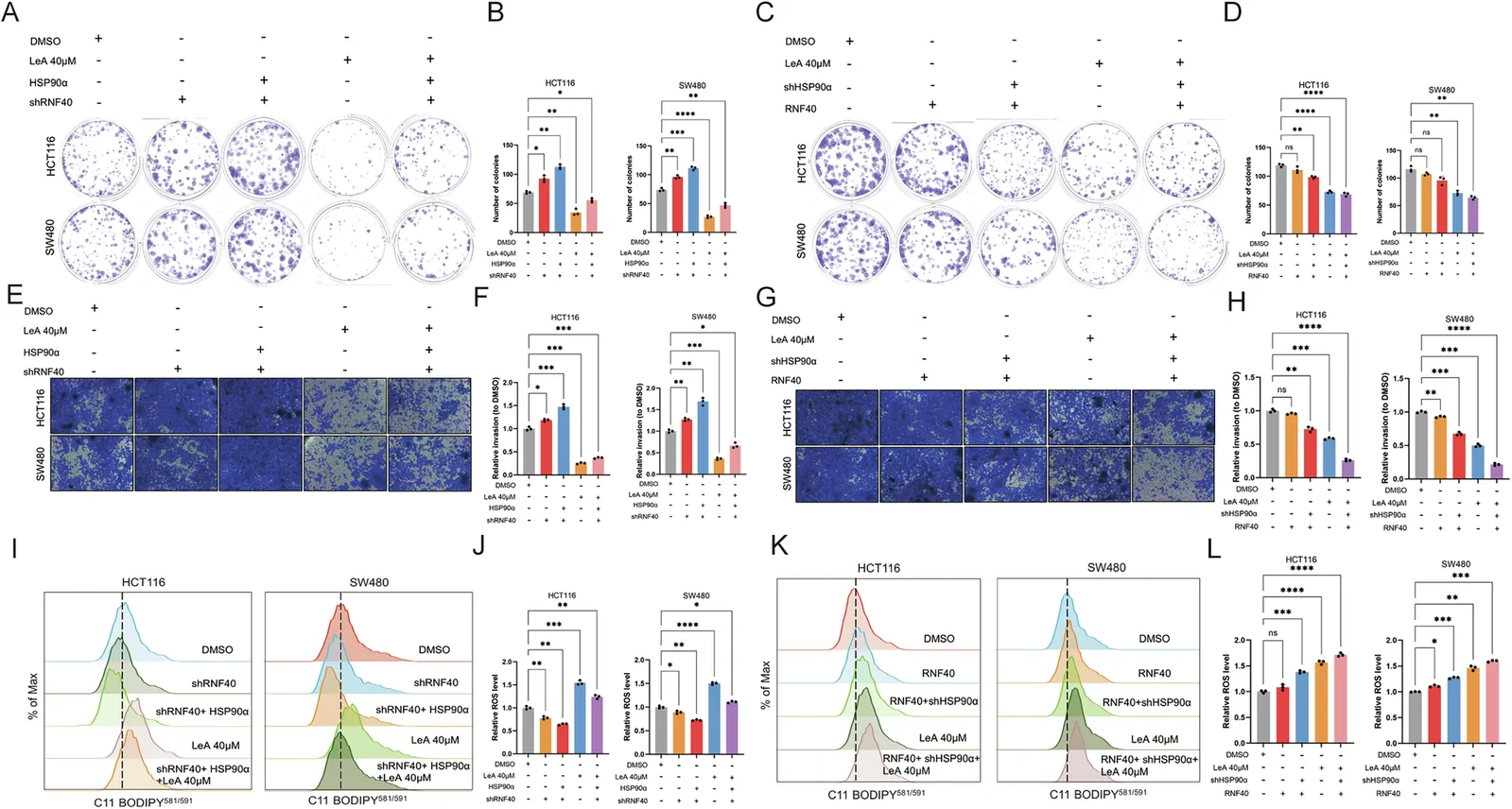
LeA regulates ferroptosis in colorectal cancer via the RNF40–HSP90α axis. **A**, **B** Stable HCT116 or SW480 cells transfected with shRNF40 or HSP90α were treated with 40 µM LeA or DMSO for 16 h. The medium was then replaced with fresh culture medium, and cells were cultured for 7 days. Subsequently, colonies were stained with crystal violet, and the number of colonies was quantified. **C**, **D** Stable HCT116 or SW480 cells transfected with RNF40 or shHSP90α were treated with 40 µM LeA or DMSO for 16 h. All other procedures remained unchanged. **E**, **F** HCT116 or SW480 cells stably transfected with shRNF40 or HSP90α were treated with 40 µM LeA or DMSO for 12 h, followed by replacement with fresh medium. After 36 h, colonies were stained with crystal violet, photographed, and quantified. **G**, **H** HCT116 or SW480 cells stably transfected with RNF40 or shHSP90α were treated with 40 µM LeA or DMSO for 12 h. All other procedures remained unchanged. **I**, **J** HCT116 or SW480 cells stably transfected with shRNF40 or HSP90α were treated with 40 µM LeA or DMSO for 12 h, incubated with the probe, and then analyzed by flow cytometry. The number of positive cells was quantified. **K**, **L** HCT116 or SW480 cells stably transfected with RNF40 or shHSP90α were treated with 40 µM LeA or DMSO for 12 h. All other procedures remained unchanged.

We next investigated how LeA modulates CRC cell behavior through the RNF40–HSP90α interaction. Using stably transfected cell lines, we first assessed changes in cell proliferation. Colony formation assays revealed that RNF40 knockdown increased colony numbers, while combined HSP90α overexpression and RNF40 knockdown further enhanced this effect. Interestingly, the cytotoxic effect of LeA on CRC cells was partially reversed in cells with HSP90α overexpression and RNF40 knockdown, suggesting that the efficacy of LeA targeting HSP90α is influenced by RNF40 deficiency (Fig. 6A, B). Conversely, RNF40 overexpression reduced CRC cell colony formation, and co-knockdown of HSP90α further decreased colony numbers. Notably, LeA treatment was ineffective under the combined RNF40 overexpression and HSP90α knockdown condition, further confirming that LeA exerts its effects primarily through HSP90α (Fig. 6C, D). In invasion assays, HSP90α overexpression together with RNF40 knockdown markedly enhanced CRC cell invasiveness and attenuated LeA’s inhibitory effect (Fig. 6E, F). In contrast, RNF40 overexpression combined with HSP90α knockdown significantly suppressed invasiveness, and co-treatment with LeA further reduced cell invasion (Fig. 6G, H). We next examined oxidative stress. The LeA-induced increase in intracellular ROS was diminished by HSP90α overexpression combined with RNF40 knockdown (Fig. 6I, J). Conversely, RNF40 overexpression together with HSP90α knockdown further elevated ROS levels (Fig. 6K, L). Similar trends were observed in MDA assays, consistent with ROS measurements (Fig. S6A, B). Collectively, these results indicate that LeA regulates CRC cell proliferation and invasiveness by modulating the RNF40–HSP90α interaction, and that these effects are closely associated with alterations in intracellular ROS and MDA levels.

### Levistilide A combined with ferroptosis inducer RSL3 inhibits colorectal cancer metastasis

**Fig. 7:**
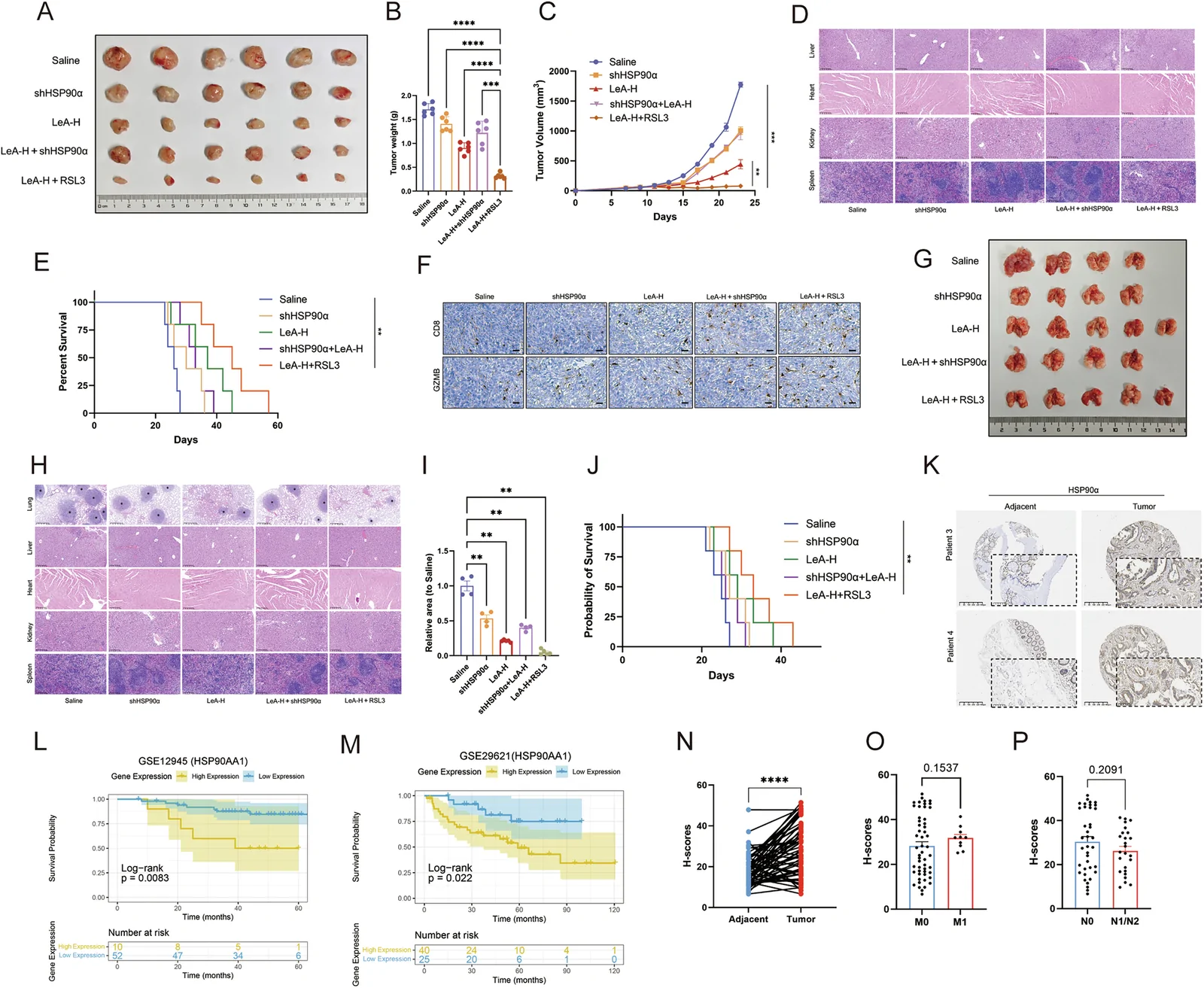
LeA in combination with RSL3 inhibits colorectal cancer metastasis. **A** Schematic of the mouse subcutaneous xenograft model. Groups included: saline control, shHSP90α, LeA-H (40 mg/kg), LeA-H (40 mg/kg) combined with shHSP90α, and LeA-H (40 mg/kg) combined with RSL3 (20 mg/kg). **B** Tumor weight quantification. **C** Tumor growth progression curve. **D** HE staining showing the morphology of the heart, liver, spleen, and kidneys in the mouse subcutaneous xenograft model. **E** Survival curves of mice in the subcutaneous xenograft model (*n* = 5, log-rank test). **F** IHC staining showing CD8 and GZMB in mouse tumor tissue sections. **G** Schematic of the mouse lung metastasis model. Groups include: saline control, shHSP90α, LeA-H (40 mg/kg), LeA-H (40 mg/kg) combined with shHSP90α, and LeA-H (40 mg/kg) combined with RSL3 (20 mg/kg). **H** HE staining showing the histological status of heart, liver, spleen, lung, and kidney in the mouse lung metastasis model. **I** Quantification of relative lung tumor area in the mouse lung metastasis model. **J** Survival curves of mice in the lung metastasis model (*n* = 5, log-rank test). **K** Representative staining of colorectal cancer tissues and paired adjacent normal tissues in the tissue microarray. Main images: ×5 magnification (scale bar: 400 μm). Insets (lower right): ×20 magnification (scale bar: 100 μm). **L**, **M** Kaplan-Meier survival curves showing overall survival of CRC patients stratified by HSP90AA1 expression levels in two independent GEO cohorts (GSE12945, *n* = 62; GSE29621, *n* = 65). Statistical significance was determined by the log-rank test. **N** H-scores of tumor tissues versus adjacent normal tissues in the tissue microarray (*n* = 63). **O** H-scores of colorectal cancer tissues with different metastasis statuses in M0 (*n* = 53) and M1 (*n* = 10) in the tissue microarray. **P** H-scores of colorectal cancer tissues with different lymph node metastasis statuses in N0 (*n* = 38) vs. N1/N2 (*n* = 25) in the tissue microarray.

We found that LeA does not exert its effects through classical ferroptosis signaling pathways. To evaluate whether LeA could synergize with the GPX4-targeting ferroptosis inducer RSL3 to further suppress colorectal cancer (CRC) metastasis, we conducted in vivo studies. In the subcutaneous xenograft model, combination treatment with LeA and RSL3 significantly inhibited tumor growth and reduced tumor volume (Fig. 7A–C). Hematoxylin and eosin (HE) staining indicated that LeA treatment had no discernible toxicity on the heart, liver, spleen, or kidney of mice (Fig. 7D). Moreover, LeA combined with RSL3 prolonged tumor-bearing survival time (Fig. 7E). Immunohistochemical (IHC) analyses revealed that this combination enhanced CD8 + T cell and macrophage infiltration, suggesting a potential remodeling of the tumor immune microenvironment (Fig. 7F). In the lung metastasis model, LeA in combination with RSL3 effectively suppressed pulmonary metastases of CRC (Fig. 7G–I), without causing significant organ toxicity (Fig. 7H, I). Importantly, the combined treatment improved overall survival in mice with lung metastases (Fig. 7J). Collectively, these data suggest that LeA is a promising candidate for treating metastatic CRC, and its combination with other therapeutic agents warrants further investigation.

Based on these findings, we hypothesized that HSP90α serves as a prognostic factor in CRC metastasis. To validate this, we analyzed HSP90α expression in tumor and paired adjacent normal tissues from clinical CRC specimens using a tissue microarray (Fig. 7K, Fig. S7A). To evaluate the prognostic significance of HSP90α in colorectal cancer, we analyzed two independent GEO cohorts. As shown in Fig. 7L, M, patients with higher HSP90AA1 expression exhibited significantly worse overall survival (log-rank *P* < 0.05), consistent with the established pro-tumorigenic role of HSP90α. We also observed that HSP90α expression was higher in tumor tissues compared with adjacent normal tissues (Fig. 7N). We then stratified CRC patients by metastatic status. Notably, M1-stage tumors showed a trend toward higher HSP90α expression compared to M0-stage tumors (Fig. 7O). However, no significant difference in HSP90α expression was observed between lymph node-negative (N0) and lymph node-positive (N1/2) patients (Fig. 7P), suggesting that HSP90α may play a more prominent role in hematogenous metastasis (e.g., lung metastasis) than in lymphatic dissemination. This is consistent with our experimental model, in which LeA suppressed CRC lung metastasis via the tail vein injection model. Collectively, these clinical data align with our mechanistic findings and support the rationale for targeting HSP90α degradation as a therapeutic strategy in CRC, particularly in advanced or metastatic disease.

## Discussion

Levistilide A (LeA) is a naturally occurring phthalide dimer isolated from the traditional Chinese medicinal herb *Ligusticum chuanxiong* Hort. Accumulating evidence has revealed diverse pharmacological activities of LeA, including anti-fibrosis [45], neuroprotective [46], and anti-depression [47]. Interesting, LeA has been reported to suppress the proliferation of breast cancer cells by inducing reactive oxygen species (ROS) accumulation and activating the Nrf2/HO-1 signaling pathway [37]. However, the direct molecular target of LeA and its role in colorectal cancer (CRC) metastasis remained largely unknown prior to this study. Here, we identify HSP90α as the direct target of LeA and elucidate a previously unrecognized mechanism whereby LeA promotes RNF40-HSP90α interaction to induce ferroptosis, thereby inhibiting CRC metastasis. Our findings expand the pharmacological understanding of LeA and provide a mechanistic basis for its potential application in metastatic CRC.

Colorectal cancer (CRC) remains a major global health burden, with metastatic disease representing the primary cause of CRC-related mortality [1, 4]. Despite advances in chemotherapy, targeted therapy, and surgical interventions, the prognosis for patients with metastatic CRC (mCRC) remains dismal, and therapeutic options are often limited [48]. The liver and lungs are the most frequent metastatic sites, and current strategies, including EGFR- or VEGFR-targeted therapies and immunotherapy, provide only modest benefits for a subset of patients and are associated with considerable financial costs [49–51]. These limitations underscore the urgent need to develop novel therapeutic agents capable of effectively suppressing mCRC progression and metastasis.

High-throughput screening (HTS) is a fundamental strategy for discovering novel small-molecule drugs and plays a pivotal role during the drug validation phase. Drug discovery is generally categorized into two main approaches: target-based and phenotypic-based screening, with the key distinction being the timing of molecular target confirmation during the hit identification process [52]. Additionally, HTS can be performed at either the biochemical or cellular level. Biochemical screening primarily utilizes purified target proteins to assess ligand binding or enzymatic inhibition in vitro [53]. In contrast, cell-based screening offers the significant advantage of simultaneously evaluating multiple targets, as chemical probes or lead compounds can be identified based on their interactions with specific targets or their ability to induce phenotypic changes in cells or organisms [54]. Among these screening strategies, cell viability assays and high-content imaging are the most commonly employed readouts. Other binding-based approaches, such as SPR, enable real-time monitoring of drug-target interactions by detecting changes in the refractive index at the metal/glass/solution interface, which are proportional to the mass of the bound analyte. In CETSA, compounds binding to a target protein stabilize its structure and increase its melting temperature [55]. Recent studies have applied diverse chemical biology approaches for small-molecule target identification. For instance, high-content screening successfully identified the anti-aging natural product ganoderic acid A [56]. Using DARTS technology, ginsenoside Rb1 was found to exert its effects by binding to and inhibiting QKI, a key protein in circular RNA biogenesis [57]. Moreover, high-throughput screening recently revealed the antidepressant vortioxetine as a potent anti-glioblastoma agent, which is now being considered for clinical investigation [58]. Therefore, selecting an appropriate high-throughput screening model and using suitable small molecules is highly advantageous for identifying antitumor natural products. In this study, we combined cell viability assays with CETSA and DARTS to explore the target of the food-derived small molecule LeA, leading to the identification of HSP90α. The repeated validation using CETSA and DARTS further reinforced the reliability of our findings, providing direct and compelling evidence for HSP90α as the molecular target of LeA.

Since its introduction in 2012, ferroptosis has emerged as a critical cell death modality in cancer research, spanning areas such as drug resistance, targeted therapy, immunotherapy, and tumor metastasis. This iron-dependent, lipid peroxidation-driven form of regulated cell death is modulated by multiple cellular metabolic pathways, including redox homeostasis, iron metabolism, mitochondrial function, and amino acid, lipid, and carbohydrate metabolism, and it is closely associated with diverse disease-related signaling pathways [59]. Notably, ferroptosis has been shown to correlate with plasma membrane dynamics and plays a pivotal role in tumor metastasis [60, 61]. Activation of ferroptotic pathways has been reported to effectively suppress tumor dissemination [24, 62]. In this study, we identified HSP90α as a critical regulator that suppresses ferroptosis. We demonstrated that the natural small molecule LeA targets HSP90α to inhibit lung metastasis of colorectal cancer. Mechanistically, we uncovered a novel RNF40-HSP90α axis, through which RNF40 promotes ubiquitination-mediated degradation of HSP90α via protein–protein interactions.

The discovery of HSP90α-targeted therapeutics has progressed through three main stages. The initial efforts focused on inhibitors targeting the N-terminal ATP-binding domain. Subsequently, subtype-specific HSP90 inhibitors were developed, followed by the identification of inhibitors targeting HSP90–co-chaperone protein–protein interactions (PPIs). However, each class of inhibitors exhibits inherent limitations. For instance, ATP-binding domain inhibitors such as 17-AAG and its derivatives are restricted by poor solubility and off-target toxicity. Among HSP90 subtype-selective inhibitors, only the TRAP1 inhibitor SMTIN-C10 has advanced to clinical testing in oncology, whereas the clinical applicability of other molecules remains unclear. Due to the limited structural knowledge of HSP90–co-chaperone PPI interfaces, further investigation of these interfaces is essential to facilitate the development of potent PPI inhibitors [63]. Recently, attention has shifted toward multi-target strategies, including proteolysis-targeting chimeras (PROTACs) and chaperone-mediated protein degradation approaches [64]. In this study, we identified a non-canonical ubiquitination mechanism of HSP90α mediated by the predominantly nuclear E3 ligase RNF40. Beyond its established role in catalyzing H2B monoubiquitination, RNF40 can regulate non-histone proteins, such as inhibiting E2F-1 via interaction with Ebp1 [65, 66]. Interestingly, recent studies demonstrated that RNF40 monoubiquitinates eEF1BδL at lysine 381, enhancing its accumulation and promoting the recruitment of p-TEFb to heat shock gene promoters, thereby facilitating heat shock gene expression [67]. Our data further confirm the direct interaction between RNF40 and HSP90α, suggesting that modulation of this axis could serve as a novel strategy for HSP90α degradation. Notably, the regulatory effect of LeA on the RNF40-HSP90α interaction resembles a molecular glue-like mechanism.

A limitation of this study is the current lack of clinical evidence, as we are actively advancing LeA toward clinical trials. In the future, we plan to explore structural modifications or synthesize derivatives of LeA to further investigate its biological activity. Moreover, the LEA-HSP90α-RNF40 ternary complex cocrystal structure will be further investigated to precisely characterize the drug-target interaction, thereby elucidating the mechanistic basis of the interaction between HSP90α and RNF40 promoted by LeA.

## Materials and methods

### Online data

The survival prognosis curves of the extracted genes, and gene expression correlation analysis were obtained from the GEO database (https://www.ncbi.nlm.nih.gov/geo/). Genetic and drug sensitivity analyses were extracted from Cancer Therapeutics Response Portal (http://portals.broadinstitute.org/ctrp/).

### Cell culture

All cells were preserved and provided by the Stem Cell and Tissue Engineering Laboratory of Chongqing Medical University. HCT116, SW480, HCT8 and MC38 cells were cultured in 1640 medium (Gibco BRL, USA) supplemented with 10% FBS (SP01002, SPERIKON, Chengdu, China) and 1% penicillin-streptomycin (SP00303-0100, SPERIKON, Chengdu, China). HEK293T cells were cultured in Dulbecco’smodified Eagle medium (Gibco BRL, USA) containing 10% FBS (SP01002, SPERIKON, Chengdu, China). Cells were cultured in incubators set at 37 °C with 5% CO2 and maintained at a constant humidity. All cell lines have been authenticated in the past 3 years by short tandem repeat (STR) genotyping and were routinely tested for mycoplasma contamination.

### Plasmids and transfection

The pLV3-CMV-HSP90AA1-3×FLAG-CopGFP, pCMV-3×Myc-RNF40, pET28a-GST, pET28a-GST-HSP90AA1, and pCMV-HA-Ub plasmids were purchased from Miaoling Biology (Wuhan, China). Knockdown plasmids of HSP90AA1, RNF40, and hsp90aa1 were constructed by complement-paired knockdown sequences and inserted into PLKO.1 plasmids, respectively, as shown in Table S1. The construction of HSP90AA1 mutation sites was performed according to the Mut Express Universal Fast Mutagenesis Kit (C216, Vazyme, Nanjing, China). The mutation primers are shown in Table S2. And, plasmids were introduced into the cells as transient transfection by Polyjet (SL100688, SignaGen, Jinan, China) following the instructions provided by the manufacturer.

To generate stably transfected cells, virus was produced by cotransfection of HEK293T cells with target plasmids, psPAX, and pMD2.G at a 4:3:1 w/w/w ratio. The medium containing the secreted virus was collected after 36 h, filtered at 0.45 micmeters, and 8 mg/ml polybrene was added to infect the target cell line.

### High-throughput screening of active compounds

Cell suspensions were prepared at a density of 1 × 10⁶ cells in 10 mL of complete culture medium and uniformly seeded into 96-well plates at a density of 1 × 10⁴ cells per well. Compounds from the Food as Medicine Compound Library (L6300, TOPSCIENCE, Shanghai, China), containing a total of 1502 small molecules, were individually added to the corresponding wells, while blank control wells were included in parallel. After 24 h of incubation, cell viability was assessed using the Cell Counting Kit-8 (CCK-8; MA0218, Meilunbio, Dalian, China), and relative absorbance values were calculated by normalization to the control group.

### Chemicals and reagents

Levistolide A (TOPSCIENCE, T6S1292), RSL3 (TOPSCIENCE, T3646), Erastin (TOPSCIENCE, T1765), Ferrostatin-1 (TOPSCIENCE, T6500), Z-VAD-FMK (TOPSCIENCE, T7020), Necrostatin-1(TOPSCIENCE, T1847), Disulfiram (TOPSCIENCE, T0054), Cycloheximide (MedChemExpress, HY-12320), Chloroquine (TOPSCIENCE, T8689), MG132 (TOPSCIENCE, T2154), Protein A/G Beads 4FF (Smart-Lifesciences, SA032025), GST-tag Purification Resin (Beyotime, P2251), Protease inhibitor cocktail (Selleck, B14001), IPTG (Beyotime, ST098), GSH (Beyotime, ST2479).

### Co-immunoprecipitation and LC/MS

Cells were harvested in NP-40 lysis buffer containing 150 mM NaCl, 50 mM Tris-HCl pH 7.4, 1% NP40 supplemented with protease inhibitor cocktail (Selleck, B14001), and the lysates were centrifuged to remove insoluble debris at 4 °C. Then, the proteins were incubated with the indicated antibodies together with Protein A/G beads for overnight at 4 °C. And, the beads were washed with NP-40 lysis buffer for three times and boiled with 1× SDS loading buffer at 95 °C for 10 min. The interacted proteins were detected using the indicated primary antibodies.

### Protein purification and GST pull-down

An overnight bacterial culture was diluted 1:20 into fresh antibiotic-containing medium and grown at 25 °C with shaking (180 rpm) to an OD₆₀₀ of ~0.6. Protein expression was induced with 0.5 mM IPTG for 10 h. Cells were harvested, resuspended in NP-40 lysis buffer supplemented with protease inhibitors, and lysed by sonication on ice (200 W, 10 s pulses, 6 cycles). The lysate was incubated with pre-equilibrated glutathione (GSH) agarose beads for 1 h. After washing three times with lysis buffer, bound proteins were eluted with elution buffer (50 mM Tris–HCl, 10 mM GSH, pH 7.8). Eluates were pooled to obtain purified GST-tagged proteins. Cells were transiently transfected with plasmids encoding the relevant tagged proteins. After 36 h, total proteins were extracted using NP-40 lysis buffer containing protease inhibitors. Pre-equilibrated GST beads (50 µL slurry) were incubated overnight at 4 °C with gentle rotation in binding buffer (NP-40 lysis buffer, 1 mM EDTA, 1 mM DTT, 10 mM MgCl₂, pH 7.8) together with 25 µg of purified GST or GST-fusion protein and cell lysates according to the experimental design. Beads were then washed twice with binding buffer, and bound proteins were eluted with elution buffer (50 mM Tris–HCl, 400 mM NaCl, 50 mM GSH, 1 mM EDTA, 1 mM DTT, pH 7.8). Eluates were mixed with 2× SDS loading buffer, boiled for 5 min, and analyzed by Western blot.

### Cell Viability assay

The Cell counting Kit-8 (MA0218, Meilunbio, Dalian, China) was employed to detected cell viability. Following the manufacturer’s instruction, the cells were placed in 96-well plates with a concentration of 1 × 10^4^ cells per well. Then, the medium containing 10% CCK8 concentration was replaced according to the provided guidelines. The measurement of absorbance was observed at a wavelength of 450 nm.

### Clone formation assay

Cells were trypsinized, resuspended in complete culture medium, and seeded into 12-well plates at a density of 500 cells per well. After 24 h, cells were treated with the designated compounds according to the experimental design. Following 12 h of treatment, the medium was replaced, and cells were further cultured for 7 days. Subsequently, the cells were washed with phosphate-buffered saline (PBS), fixed with 4% paraformaldehyde for 15 min, and stained with crystal violet solution (BL802A, Biosharp, Beijing, China) for 15 min. After thorough washing with deionized water and air-drying, images of the stained colonies were captured.

### Scratch assay

Cells were trypsinized, resuspended in complete culture medium, and seeded into 6-well plates. Once the cells reached full confluence, a sterile 200 µL pipette tip was used to create a vertical scratch in each well. After 24 h, one random field per group was selected for imaging, and representative images of the wound area were captured.

### Transwell

Matrigel (354277, Corning, USA) was diluted 1:6 with culture medium on ice, and 50 µL was added to each Transwell insert (14341, LABSELECT, Beijing, China) to form a gel at 37 °C for 2 h. Subsequently, 2 × 10⁴ trypsinized cells were seeded into the upper chamber. The lower chamber was filled with 500 µL of culture medium containing 20% FBS, with or without the indicated drugs. After 24 h of incubation at 37 °C, the inserts were washed with PBS, fixed with 5% paraformaldehyde, and stained with crystal violet (Biosharp, Beijing, China). Non-invasive cells on the upper membrane surface were removed with a cotton swab. Invaded cells on the lower surface were imaged under a microscope.

### Western blot

Western blot analysis was performed as described previously [24]. The concentrations of antibodies used in this and subsequent experiments are shown in Table S3. Protein markers were purchased from Epizyme Biotech (WJ102, shanghai, China). WB exposure solution was purchased from Meilunbio (MA0187, Dalian, China). The exposure results obtained were quantified by imagej software.

### RNA sequencing and bioinformatics analysis

Total RNA was extracted from the samples, and mRNA was subsequently enriched. Sequencing libraries were constructed, quality-checked, and sequenced on an Illumina NovaSeq 6000 platform with paired-end 150 bp reads. Raw sequencing reads were subjected to quality control, alignment to the reference genome, and quantification, followed by downstream bioinformatic analyses. All sequencing experiments were performed by APExBIO Technology (Shanghai, China).

Differentially expressed genes (DEGs) were identified based on transcript per million (TPM) matrices, using thresholds of |Log₂ fold change|≥ 1 and *P* < 0.05. Heatmaps and volcano plots were generated using the R packages *ggplot2*, *pheatmap*, *ggsci*, and *dplyr*. Functional enrichment analyses, including Gene Ontology (GO), Kyoto Encyclopedia of Genes and Genomes (KEGG), and Gene Set Enrichment Analysis (GSEA), were performed using the R packages *ggplot2*, *pheatmap*, *ggsci*, *org.Hs.eg.db*, *clusterProfiler*, *enrichplot*, *GSEABase*, and *GSVA*.

### RT-qPCR

Total RNA was extracted using the Super FastPure Cell RNA Isolation Kit (RC102, Vazyme, Nanjing, China) according to the manufacturer’s instructions. Complementary DNA (cDNA) was synthesized from the extracted RNA using the UnionScript First-Strand cDNA Synthesis Mix (SR511, Genesand, Beijing, China). Quantitative PCR (qPCR) was performed using the SYBR Prime qPCR Kit (BG0014, BIOGROUND, Chongqing, China) with gene-specific primers for *HSP90AA1* (forward: 5′-CCCTTCCACGTCTCTGCATT-3′; reverse: 5′-CTCAACCTCCTCCTCCTCCA-3′) and *ACTB* (forward: 5′-CATGTACGTTGCTATCCAGGC-3′; reverse: 5′-CTCCTTAATGTCACGCACGAT-3′) as the internal control. Relative mRNA expression levels were calculated using the 2^−ΔΔCt method and normalized to *ACTB* expression.

### Immunofluorescence

Cells were seeded onto confocal dishes. After treatment, cells were washed with PBS, fixed with 4% paraformaldehyde for 15 min, and permeabilized with 0.2% Triton X-100 for 10 min. Non-specific binding was blocked with immunofluorescence blocking solution (P0260, Beyotime). Cells were then incubated with primary antibodies at 4 °C overnight, followed by incubation with fluorescent secondary antibodies for 1 h at room temperature in the dark. After counterstaining nuclei with DAPI (P0131, Beyotime), images were captured using a Leica TCS SP8 confocal microscope.

### Lipid peroxidation MDA assay

Malondialdehyde (MDA) levels in cell lysates were measured using the Lipid Peroxidation MDA Assay Kit (S0131, Beyotime) following the manufacturer’s instructions. Absorbance was measured at 532 nm, and MDA concentrations were calculated based on a standard curve.

### Cellular lipid-ROS assay

Cells were incubated with 5 μM BODIPY 581/591 C11 probe (D3861, Invitrogen, Carlsbad, US) for 30 min at 37°C. Cells were digested using trypsin and resuspended in PBS. Then, cells were analyzed by flow cytometry.

### GSH/GSSG ratio assay

Intracellular GSH and GSSG levels were measured using a GSH/GSSG Assay Kit (Beyotime, S0053, Shanghai, China) according to the manufacturer’s protocol. Briefly, Cells were seeded in 6-well plates and treated with DMSO, Fer-1 (5 μM), LeA (20 or 40 μM), or LeA plus Fer-1 (5 μM) for 12 h. Cells were then harvested, lysed, and centrifuged at 10,000 × *g* for 10 min at 4 °C. Total GSH and GSSG were determined using a microplate reader at 412 nm. The GSH/GSSG ratio was calculated as (total GSH–GSSG) / GSSG.

### Intracellular Fe^2+^ assay

Intracellular ferrous iron (Fe²⁺) levels were detected using FerroOrange (Dojindo, F374, Kumamoto, Japan). Following treatment with DMSO, Fer-1 (5 μM), LeA (20 or 40 μM), or LeA plus Fer-1 (5 μM) for 16 h, cells were washed twice with PBS and incubated with 1 μM FerroOrange in serum-free 1640 at 37 °C for 30 min in the dark. Cells were then trypsinized, resuspended in PBS, and immediately analyzed by flow cytometry (BD FACSCanto II, BD Biosciences, San Jose, CA, USA) using the FL1 channel (excitation 540 nm, emission 580 nm).

### Transmission electron microscopy assay

Cultured cells were washed to remove the culture medium and subsequently fixed with 2.5% glutaraldehyde at room temperature for 30 min. After the initial fixation, the fixative was replaced with fresh 2.5% glutaraldehyde, and the samples were transferred to 4 °C for storage until further processing. The fixed cells were then prepared for transmission electron microscopy (TEM) observation following standard procedures.

### SPR assay

SPR analysis was performed on a Biacore 1 K instrument (Cytiva, MA, USA). The ligand protein (HSP90α or RNF40) was immobilized on a CM5 sensor chip via standard amine coupling using EDC/NHS chemistry, with a reference channel prepared in parallel. Serially diluted compounds were injected over the chip at a flow rate of 30 µL/min for 60 s. The chip was regenerated with 10 mM glycine-HCl (pH 2.0) between cycles. Binding responses were recorded, reference-subtracted, and globally fitted to a 1:1 Langmuir model using Biacore Insight Evaluation Software (Cytiva, Marlborough, MA, USA) to determine kinetic constants.

For the ternary interaction analysis, RNF40 protein was pre-incubated with LeA (1 μM) for 1 h at room temperature, and the mixture was then injected over a CM5 chip immobilized with HSP90α. The binding response was caculated by Biacore Insight Evaluation Software (Cytiva, Marlborough, MA, USA).

### Cellular thermal shift assay (CETSA)

Cells were trypsinized, washed with PBS, and lysed through three cycles of freeze-thaw in liquid nitrogen. Following centrifugation at 4 °C, the clarified supernatant was collected. Aliquots of the supernatant were incubated with DMSO or indicated compounds for 2 h at room temperature. Samples were then subjected to temperature gradients as specified, centrifuged again, and the final supernatants were collected for LC–MS analysis or mixed with SDS loading buffer for Western blot.

### Drug affinity responsive target stability (DARTS)

Cells were washed twice with PBS and lysed using NP-40 lysis buffer. Lysates were centrifuged at 4 °C, and the supernatants were collected. Supernatants were divided into experimental groups and treated with either DMSO or the designated compounds. *Streptomyces griseus*-derived protease (Roche, 10165921001, Mannheim, Germany) was added to each group at varying concentrations and incubated as indicated. Samples were subsequently analyzed by SDS–PAGE followed by either liquid chromatography–mass spectrometry (LC-MS) for target protein identification or Western blot for validation.

### In vivo ubiquitination assay

Cells were treated with the proteasome inhibitor MG132 for 6 h prior to harvest. Total cellular proteins were then extracted, and immunoprecipitation was performed using an anti-Myc antibody. The ubiquitination level of HSP90α was subsequently assessed by immunoblotting with an anti-HA antibody.

### Molecular dynamics simulations

Molecular dynamics (MD) simulations of the protein–ligand complex were performed in GROMACS 2025.2. The protein and ligand were modeled with the CHARMM36 force field, solvated in a TIP3P water box, and neutralized with 150 mM NaCl. After energy minimization, the system was equilibrated under NVT and NPT ensembles (100 ps each). A production run of 100 ns was conducted using a 2 fs timestep. Trajectories were analyzed for backbone stability (RMSD) and residue flexibility (RMSF).

### Animal experiments

Animals were kept under specific pathogen free (SPF) conditions in accordance with animal welfare laws and regulations. Ethical approval for this study was obtained from Institutional Animal Care and Use Committ of Chongqing Medical University (IACUC-CQMU-2025-12011). All six-week-old male C57BL/6 J mice were obtained from the Experimental Animal Center of Chongqing Medical University, and maintained under SPF conditions with a 12-h light/dark cycle, controlled temperature (22 ± 2 °C), and ad libitum access to food and water. The experimentalists were blinded to the information about tumor or lung when organs were excised and weighed.

#### Subcutaneous xenograft model

For the LeA monotherapy experiment (Fig. 1L–O), MC38 cells (5 × 10⁵ in 100 μL of sterile PBS) were injected subcutaneously into the right flank of each mouse. Seven days after inoculation, when tumors reached a volume of approximately 50–100 mm³, mice were randomly assigned to three groups (*n* = 6 per group): (1) vehicle control (physiological saline, intraperitoneal injection, every other day), (2) LeA low-dose (20 mg/kg in 100 μL saline, intraperitoneal injection, every other day), and (3) LeA high-dose (40 mg/kg in 100 μL saline, intraperitoneal injection, every other day).

For the combination therapy experiment, MC38 cells or MC38-shHsp90aa1 cells (5 × 10⁵ in 100 μL of sterile PBS) were injected subcutaneously. Seven days after inoculation, mice were randomly assigned to five groups (*n* = 6 per group): (1) Saline in MC38, (2) MC38-shHsp90aa1, (3) LeA-H (40 mg/kg) in MC38, (4) LeA-H (40 mg/kg) in MC38-shHsp90aa1, and (5) LeA-H (40 mg/kg) plus RSL3 (5 mg/kg) in MC38. All treatments were administered intraperitoneally every other day.

Tumor dimensions were measured every two days using a digital caliper, and tumor volume was calculated using the formula: tumor volume = length × (width)² / 2. Mice were euthanized by cervical dislocation when the tumor volume exceeded 2000 mm³, when tumors showed ulceration or bleeding, or when the mice exhibited signs of distress (e.g., significant weight loss >20%, lethargy, or hunched posture). At the end of the experiment, tumors were excised, weighed, and either fixed in 10% neutral buffered formalin for histological analysis.

#### Lung metastasis model

For the LeA monotherapy experiment, MC38 cells (2 × 10⁵ in 100 μL of sterile PBS) were injected into the lateral tail vein of each mouse. Seven days after injection, mice were randomly assigned to three groups (*n* = 6 per group): vehicle control, LeA low-dose (20 mg/kg), and LeA high-dose (40 mg/kg). Treatments were administered intraperitoneally every other day for 4 weeks.

For the combination therapy experiment, MC38 cells or MC38-shHsp90aa1 cells (2 × 10⁵ in 100 μL of sterile PBS) were injected into the lateral tail vein. Seven days after injection, mice were randomly assigned to five groups (*n* = 6 per group): (1) Saline in MC38, (2) MC38-shHsp90aa1, (3) LeA-H (40 mg/kg) in MC38, (4) LeA-H (40 mg/kg) in MC38-shHsp90aa1, and (5) LeA-H (40 mg/kg) plus RSL3 (5 mg/kg) in MC38. Treatments were administered intraperitoneally every other day.

At the end of the experiments, mice were euthanized, and the lungs were immediately excised, washed with PBS, and fixed in 10% neutral buffered formalin for 24 h. Fixed lungs were embedded in paraffin, sectioned (5 μm thickness), and subjected to hematoxylin and eosin (HE) staining.

#### Survival analysis

For survival studies, mice bearing subcutaneous xenografts or lung metastases were treated as described above (*n* = 5 per group). Mice were monitored daily, and survival was recorded until natural death or until humane endpoints were reached (tumor volume > 2000 mm³, weight loss > 20%, or severe respiratory distress). Survival curves were generated using the Kaplan-Meier method and compared using the log-rank test.

### Hematoxylin and eosin (HE) staining

Tumor or mouse tissue specimens were collected and immediately fixed in 10% neutral buffered formalin. After fixation, tissues were dehydrated, embedded in paraffin, and sectioned into 5 μm thick slices. Sections were subjected to HE staining following standard protocols, allowing visualization of cell nuclei and cytoplasm for histopathological examination.

### Immunohistochemistry staining

Formalin-fixed, paraffin-embedded mouse tumor tissues were sectioned and subjected to immunohistochemical (IHC) analysis. The selected tissue sections were incubated with specific primary antibodies at the indicated dilutions, including anti-CD8 (Abclonal, A11856) and anti-GZMB (Abclonal, A2557).

### Tissue microarray

A colorectal cancer tissue microarray (YP-CRC1261), comprising 63 paired colorectal cancer tissues and corresponding adjacent non-tumor tissues, was obtained from Shanghai YBLBIO Biotech Co., Ltd. Immunohistochemical staining was performed using an anti-HSP90α antibody (ZEN-BIOSCIENCE, R380809) according to the manufacturer’s protocol.

### BioRender statement

Graphical abstract was generated using BioRender (https://www.biorender.com). Publication licenses were secured in compliance with BioRender’s academic licensing terms, and appropriate attribution is provided within the corresponding figure legends.

### Statistical analysis

All bioinformatics statistical analyses were performed using R software (version: R 4.4.1). All statistics were calculated using GraphPad Prism software (version 8.4.0). The results were repeated at least three experiments (*n* = 3). For comparisons involving more than two groups, one-way ANOVA with Dunnett’s or Tukey’s multiple comparisons test, or two-way ANOVA with Sidak’s or Tukey’s multiple comparisons test were applied. Survival analysis utilized the log-rank test. Statistical significance was determined as values of *P* < 0.05. *P < 0.05, **P < 0.01, ***P < 0.001, ****P < 0.001, ns: not statistically significant. Data were presented as mean ± SEM.

## Supplementary information

Supplement figure

Supplement Table

Original full length western blots

## Data Availability

The mRNA-seq data and protein mass spectrometry have been deposited in BIG Sub under the accession code OMIX013957 and OMIX014017. The original contributions presented in the study are included in the article/Supplementary Material, further inquiries can be directed to the corresponding authors.
