# Supplementary material for: Levistilide A promotes ferroptosis through the RNF40-HSP90α axis and inhibit colorectal cancer lung metastasis
Source: Cell Death Discov. 2026 Aug 15;12:453. doi: 10.1038/s41420-026-03265-x (PMC13754910; doi:10.1038/s41420-026-03265-x)
Supplement: Supplementary file 1 [file 41420_2026_3265_MOESM3_ESM.pdf]

Figure1T

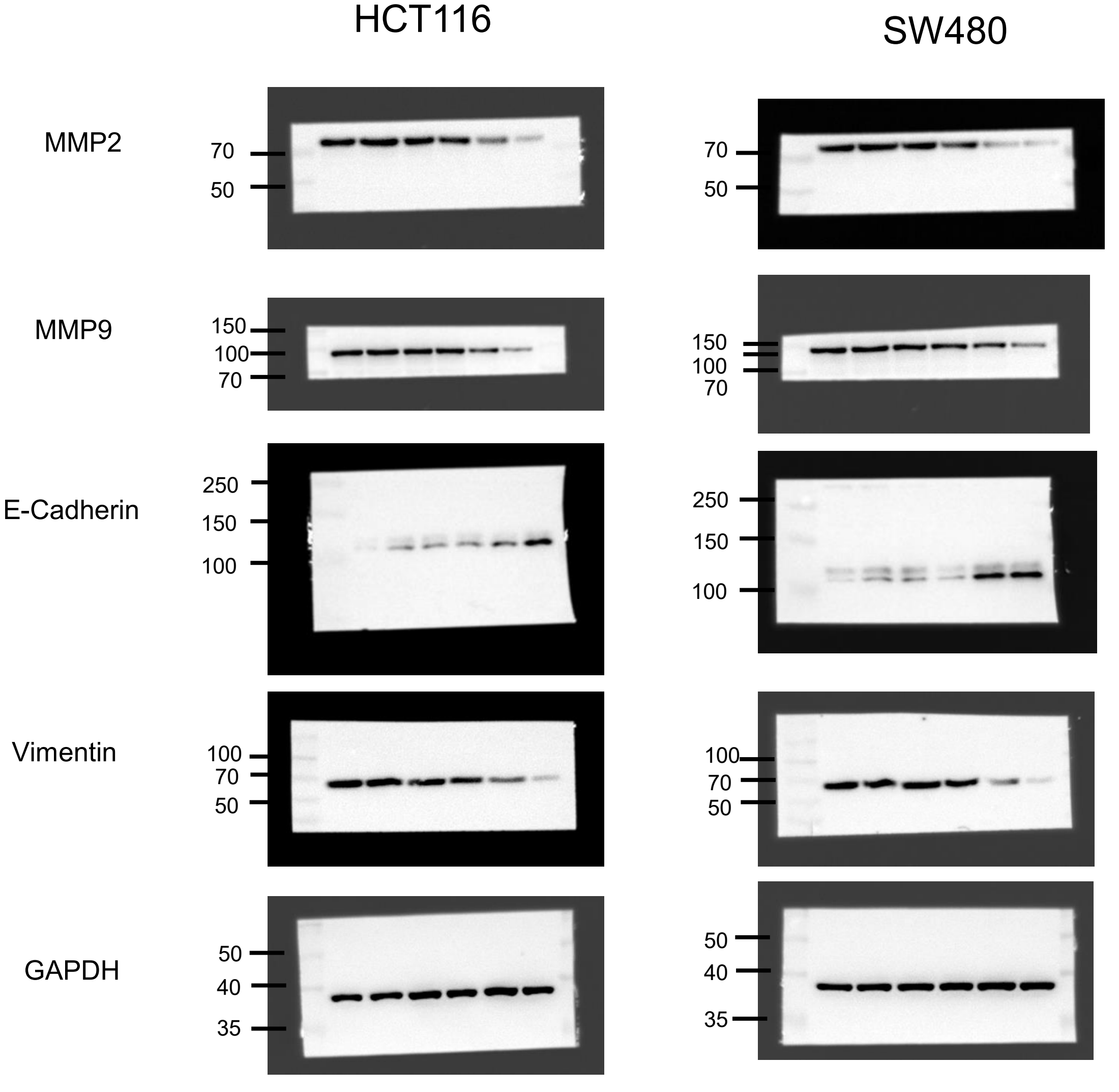
HCT116
SW480
MMP2
70
50
MMP9
150
100
70
E-Cadherin
250
150
100
Vimentin
100
70
50
GAPDH
50
40
35

Figure2N

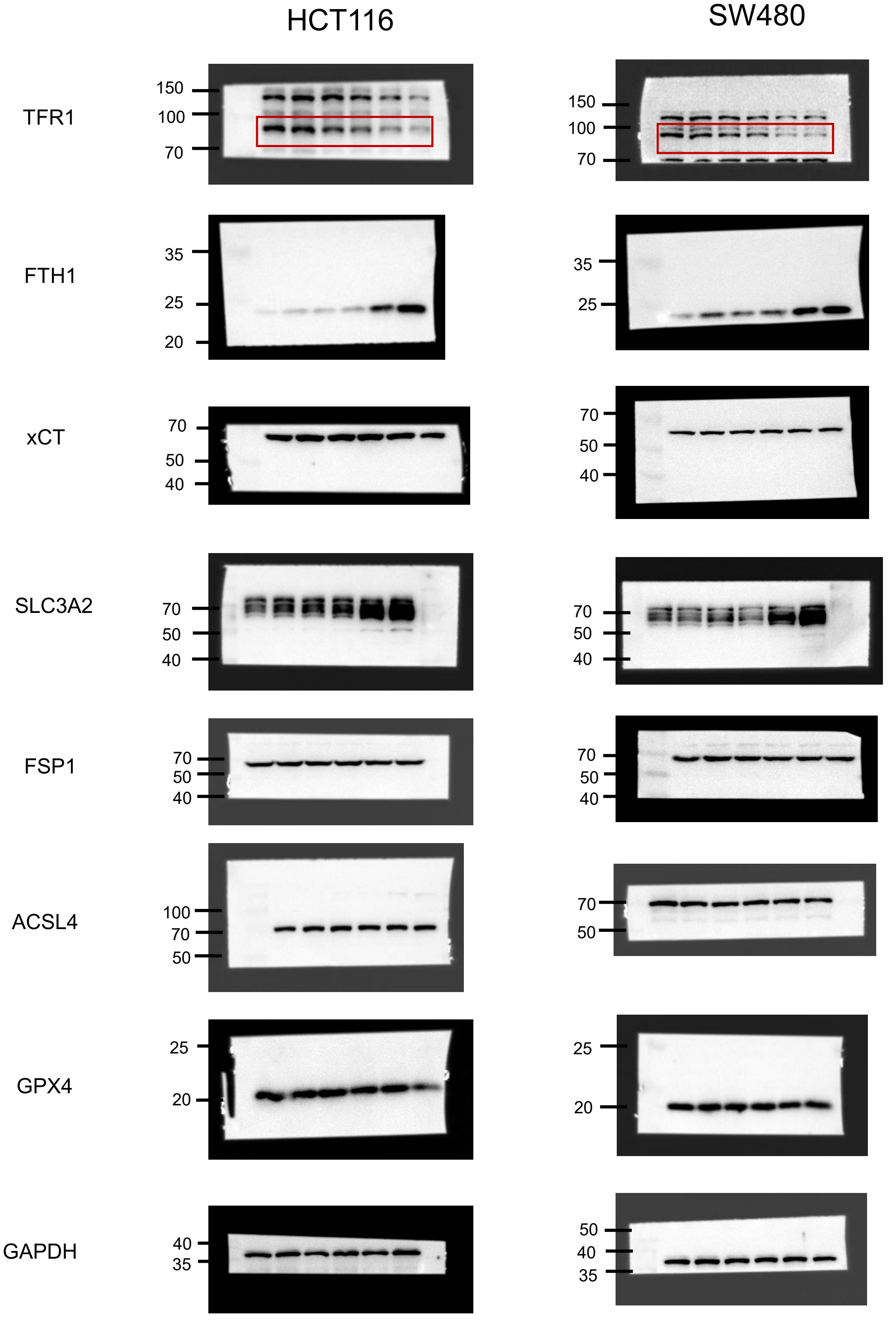
HCT116
SW480
TFR1
150
100
70
FTH1
35
25
20
xCT
70
50
40
SLC3A2
70
50
40
FSP1
70
50
40
ACSL4
100
70
50
GPX4
25
20
GAPDH
40
35
50

Figure3D

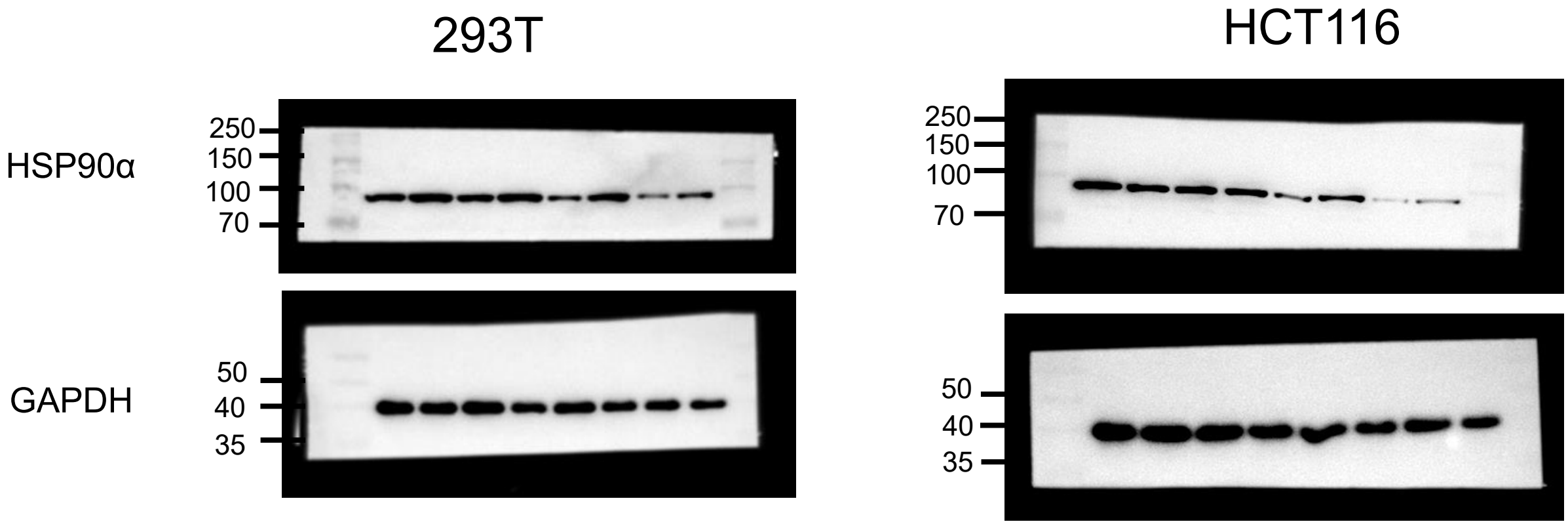

Figure3E

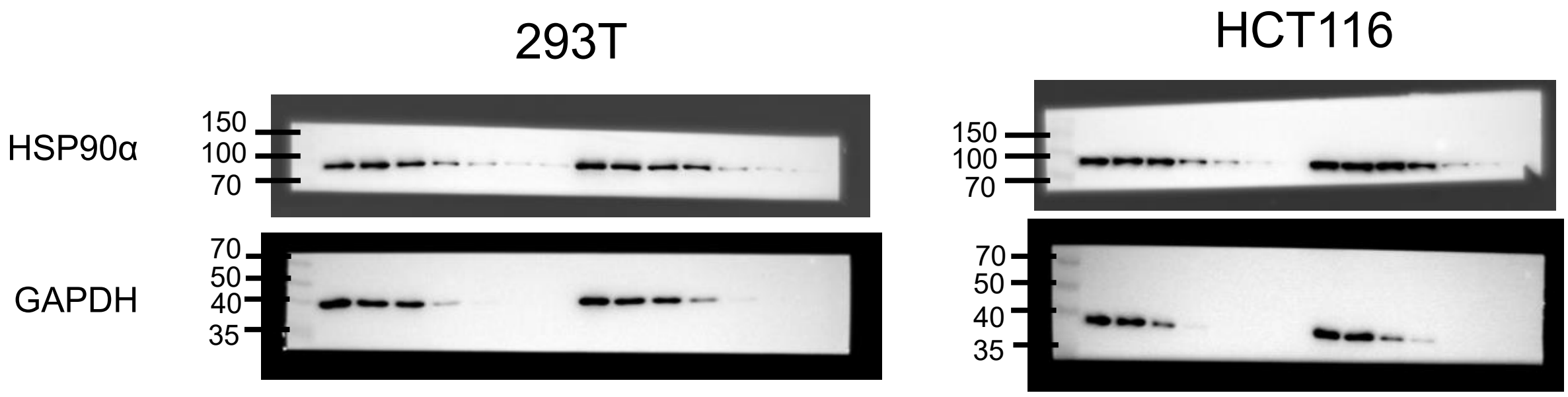

Figure3G

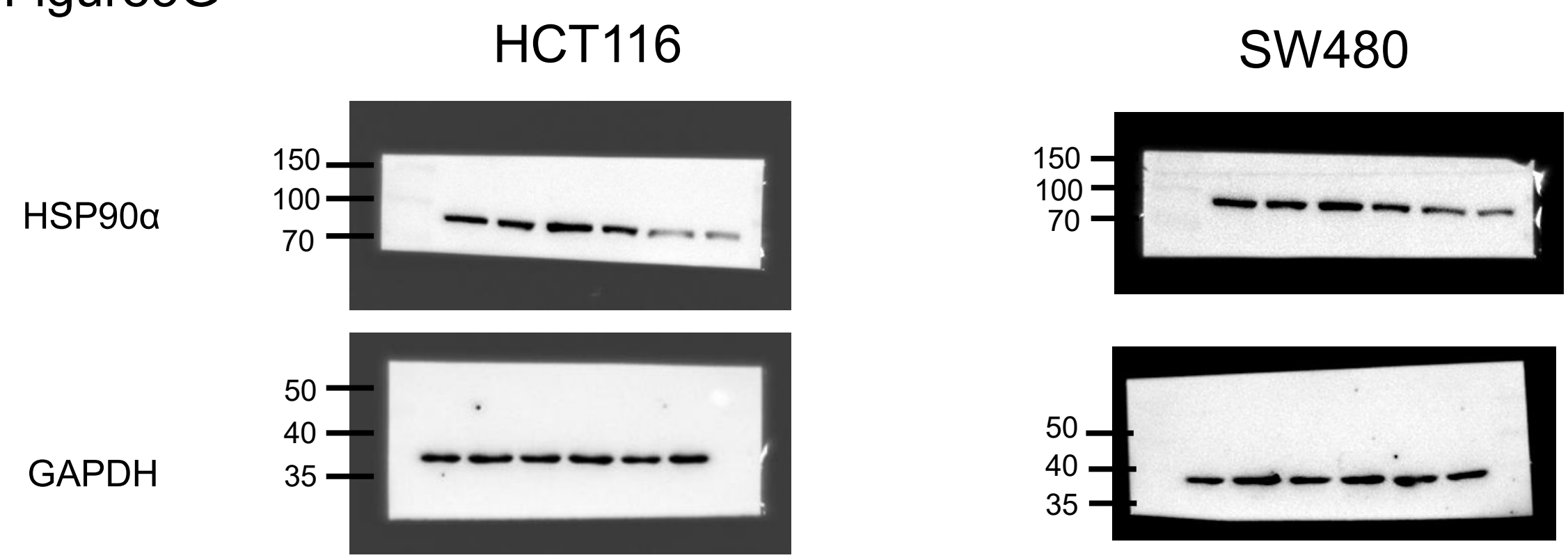

Figure3H

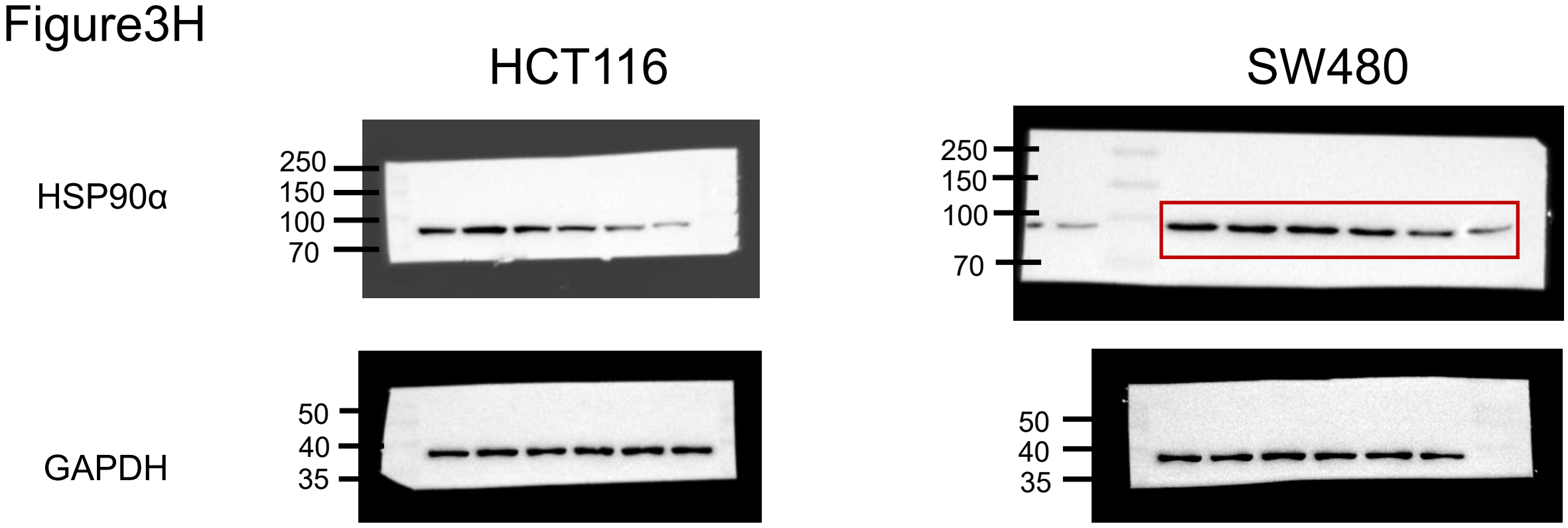

Figure3J

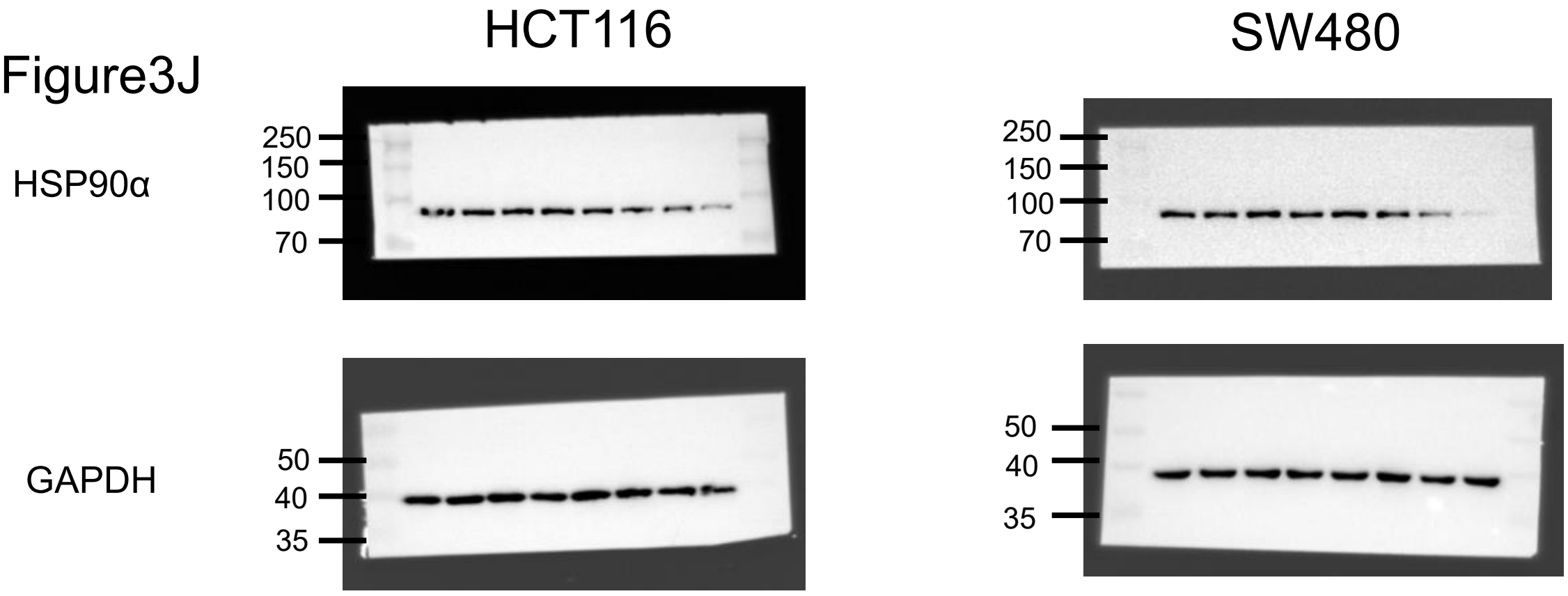

Figure3L

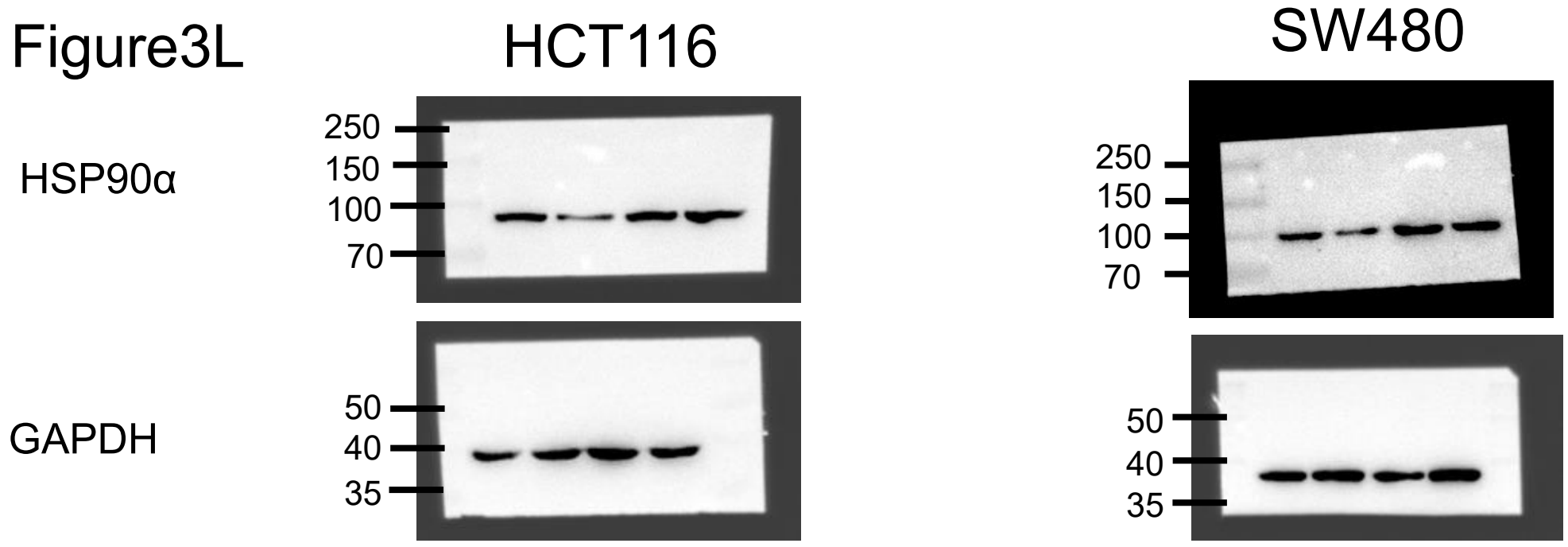

Figure4B

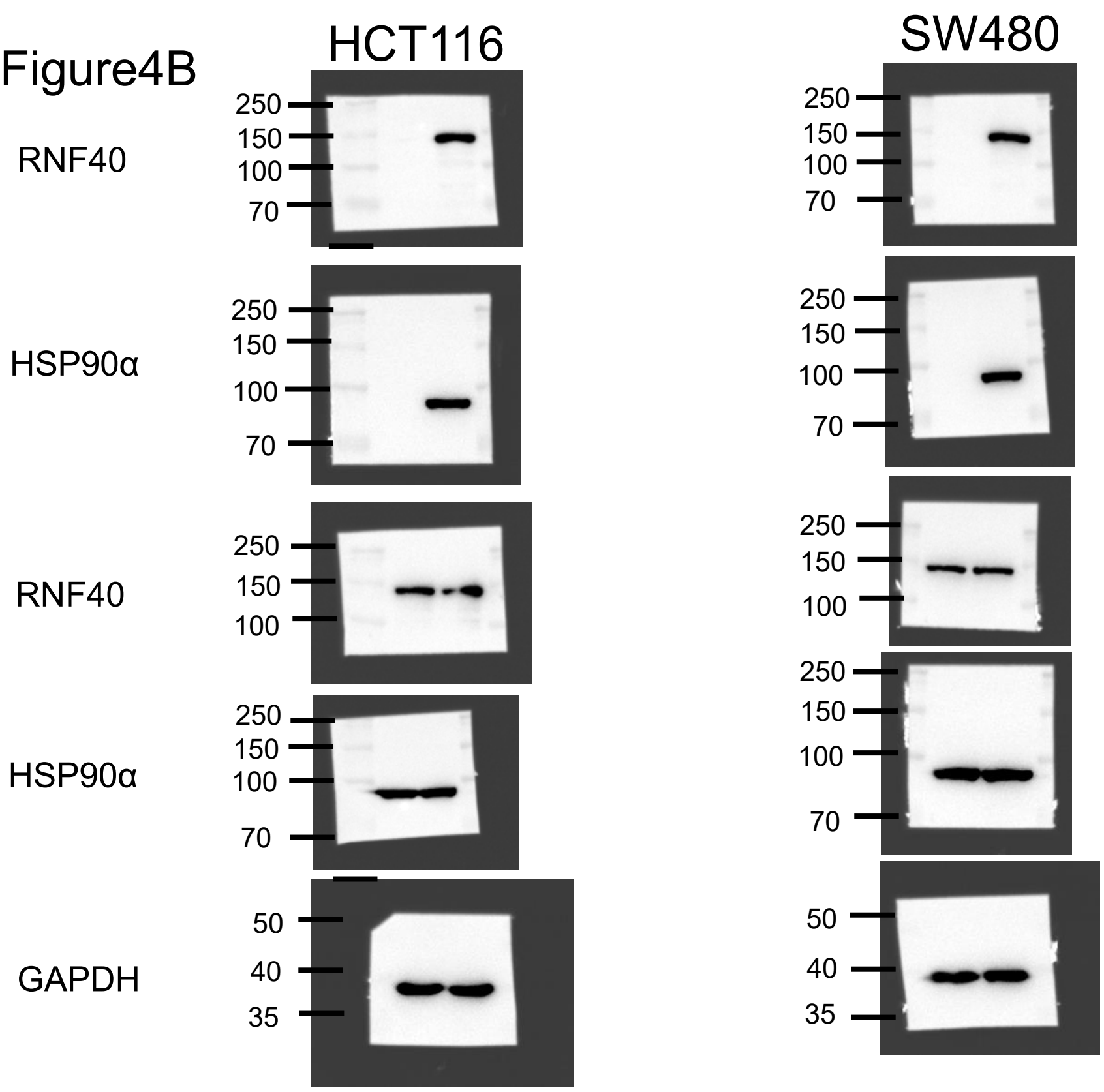

Figure4C

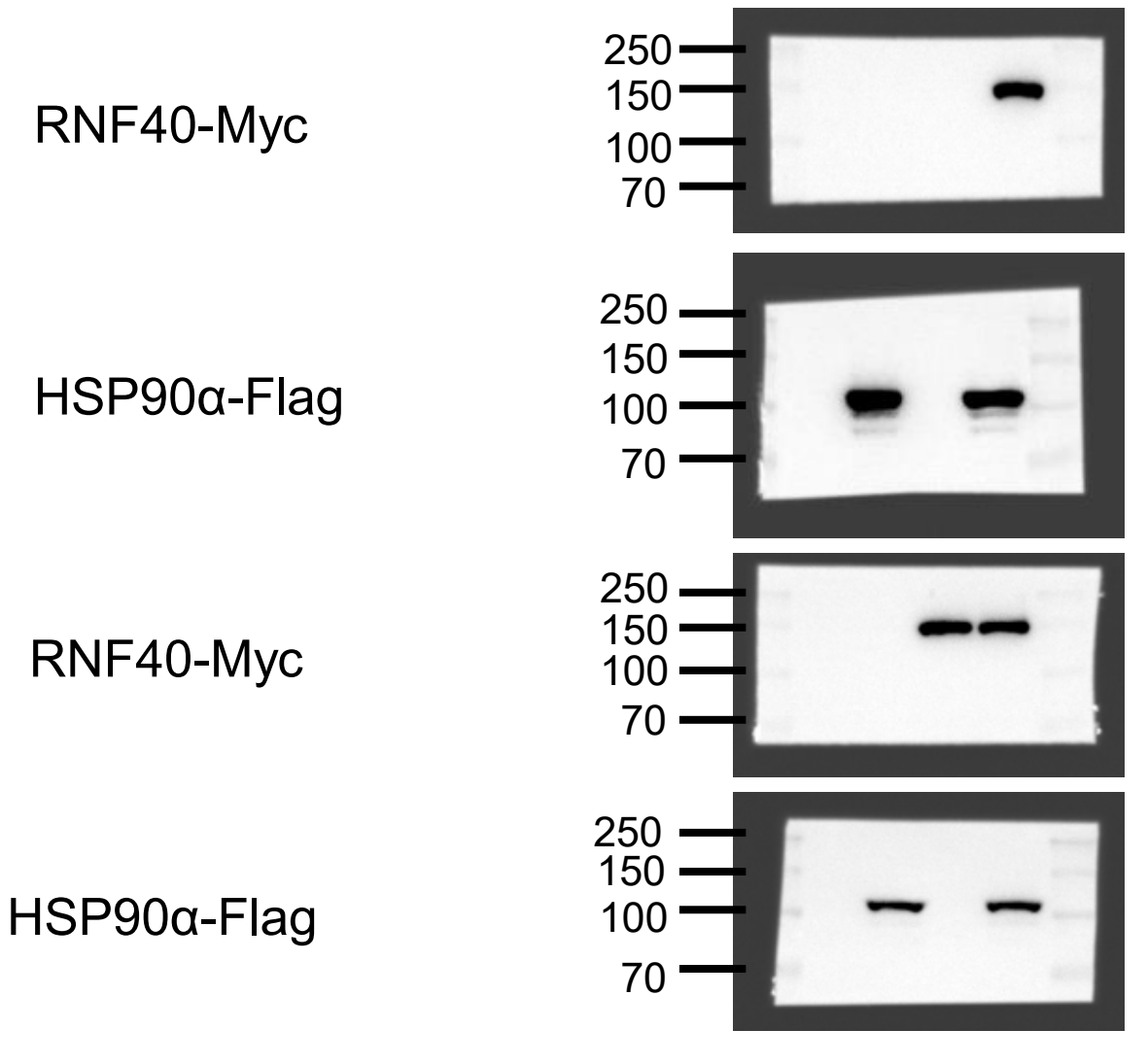

Figure4D

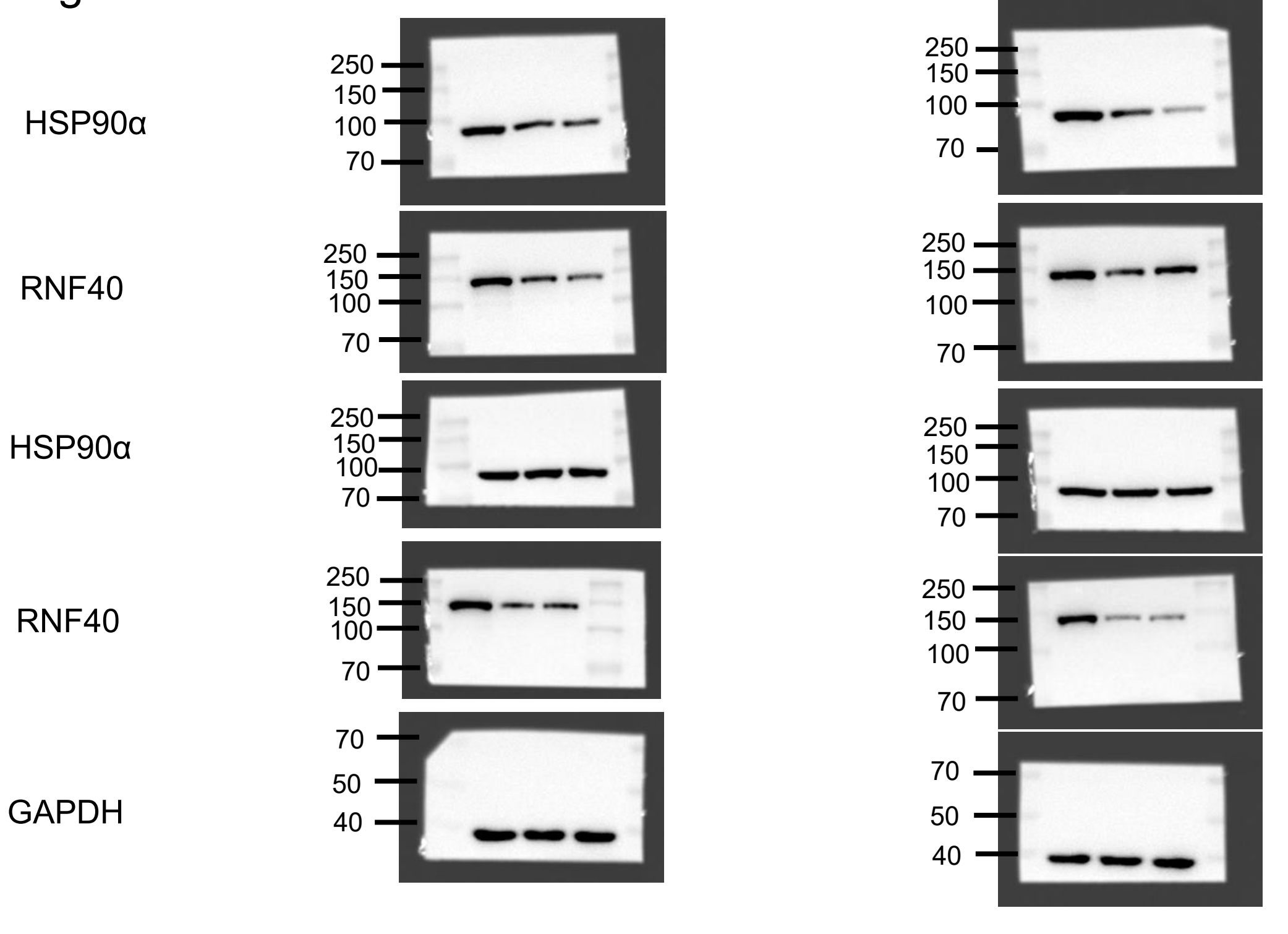

Figure4E

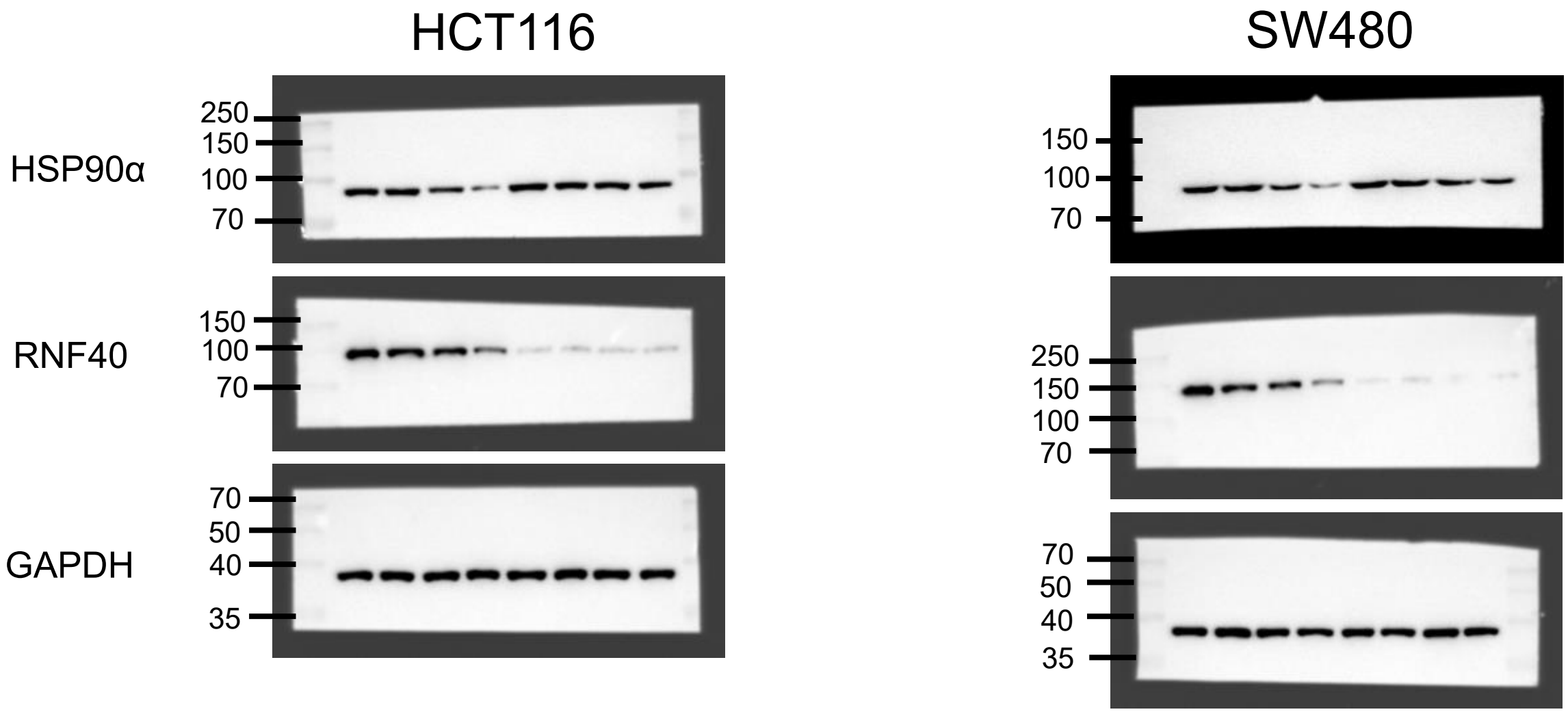

Figure4G

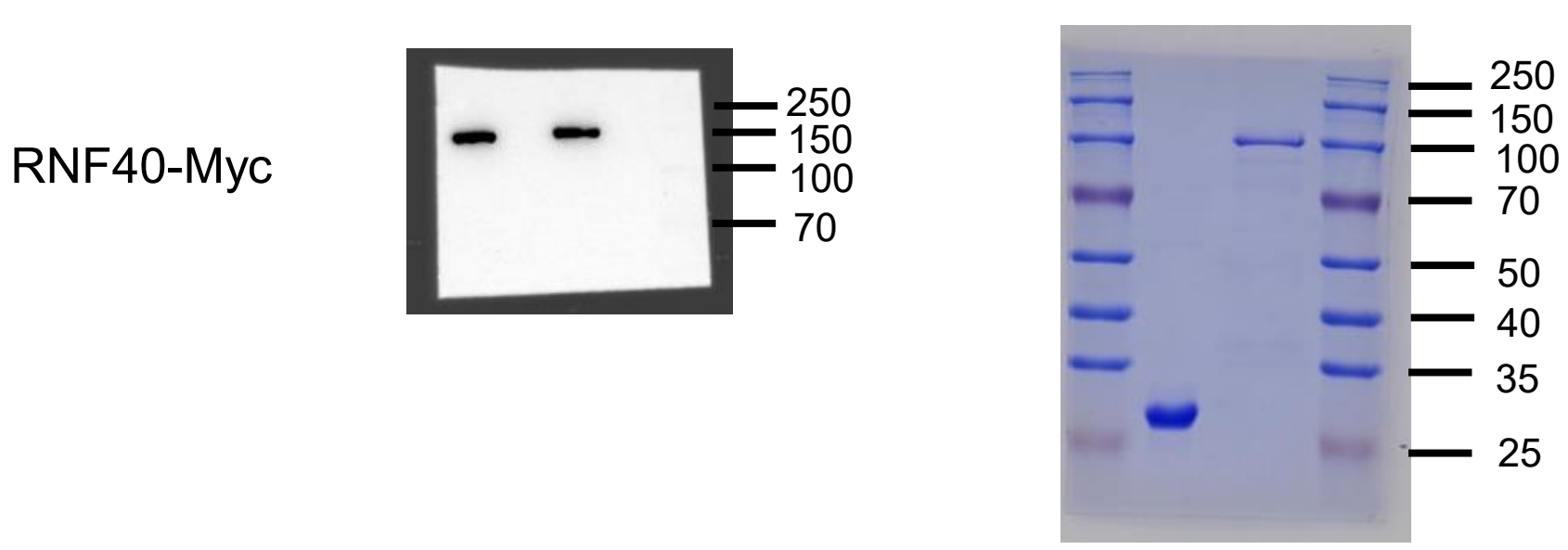

Figure4H

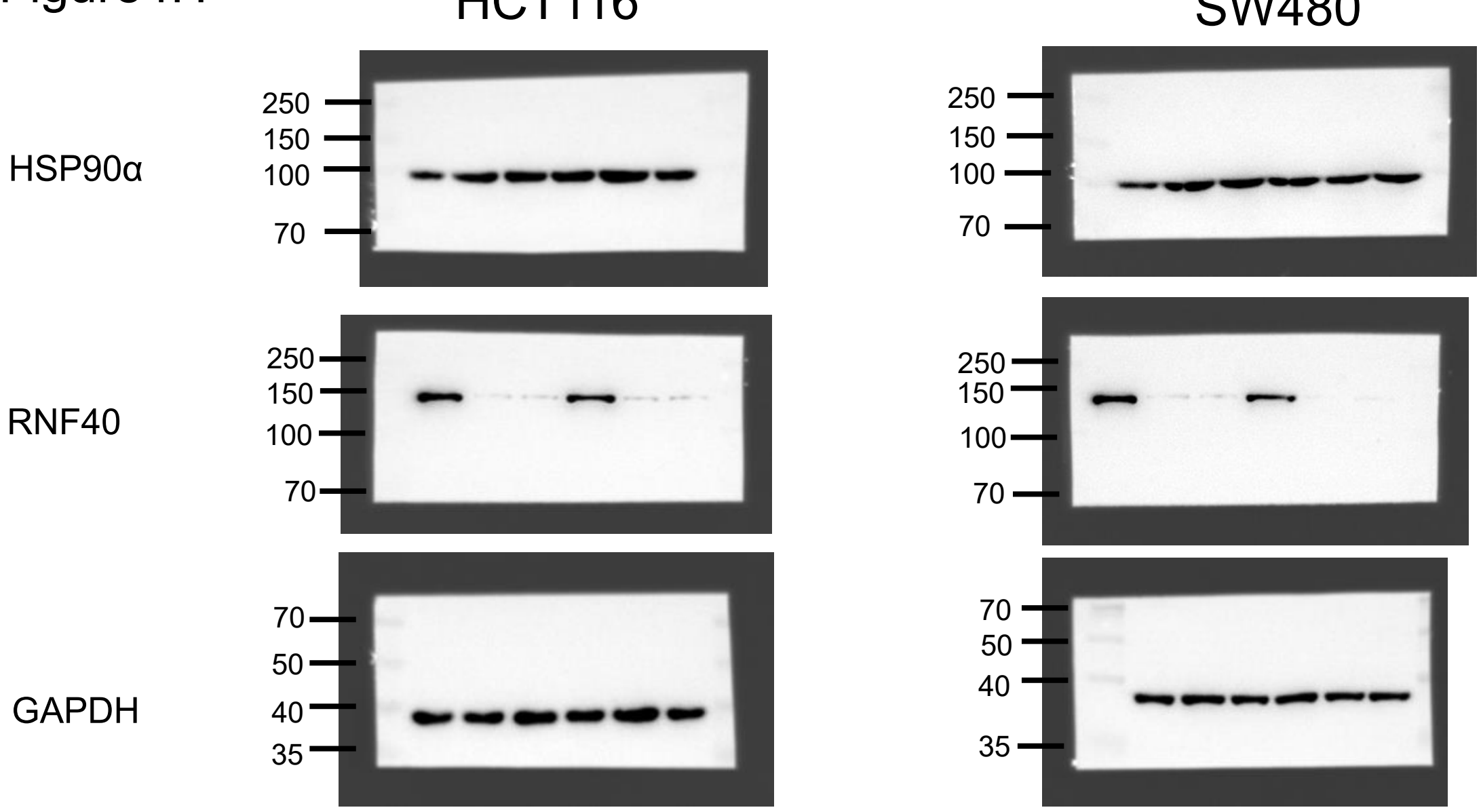

Figure4J

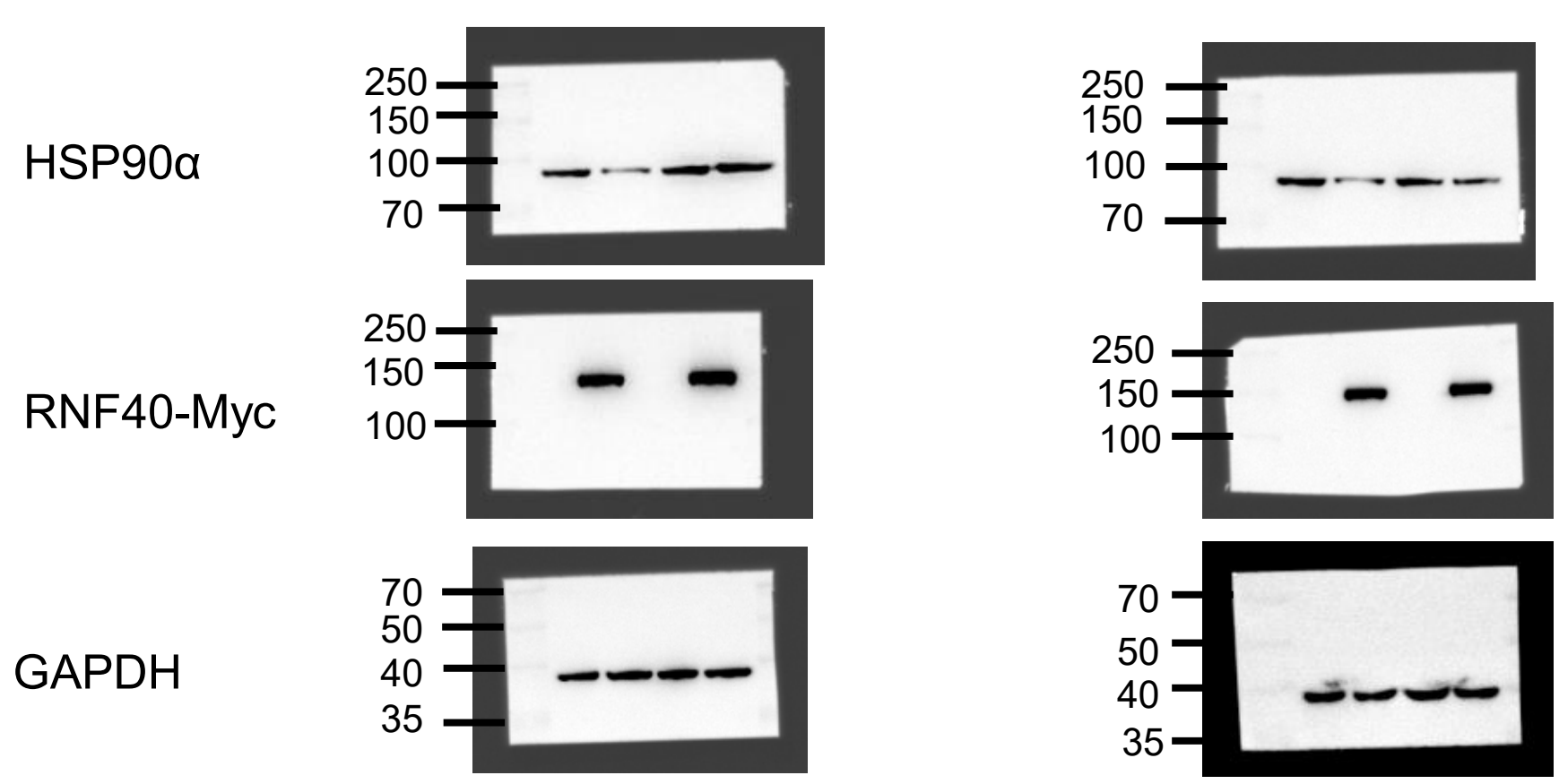

Figure4L

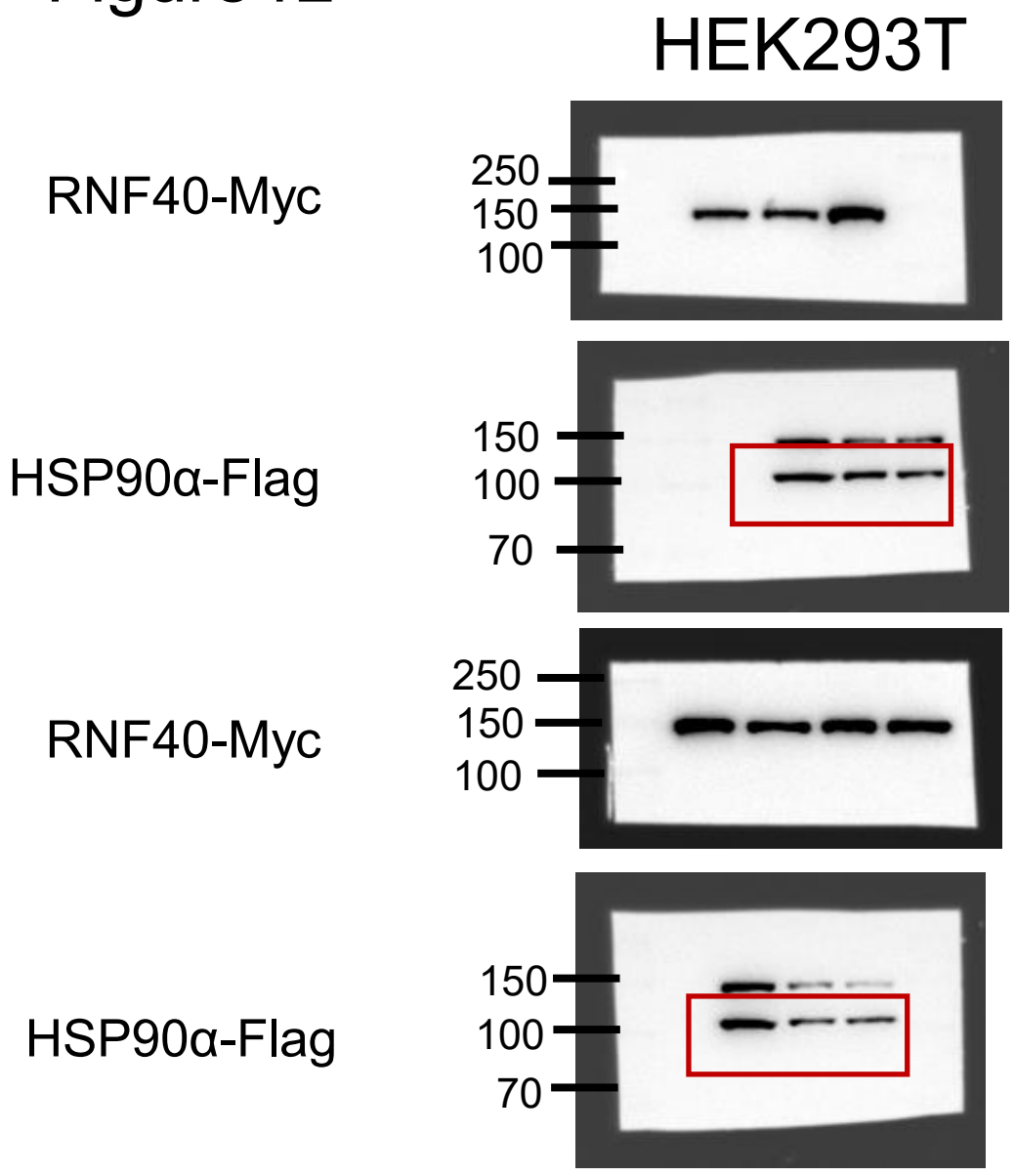

Figure4M

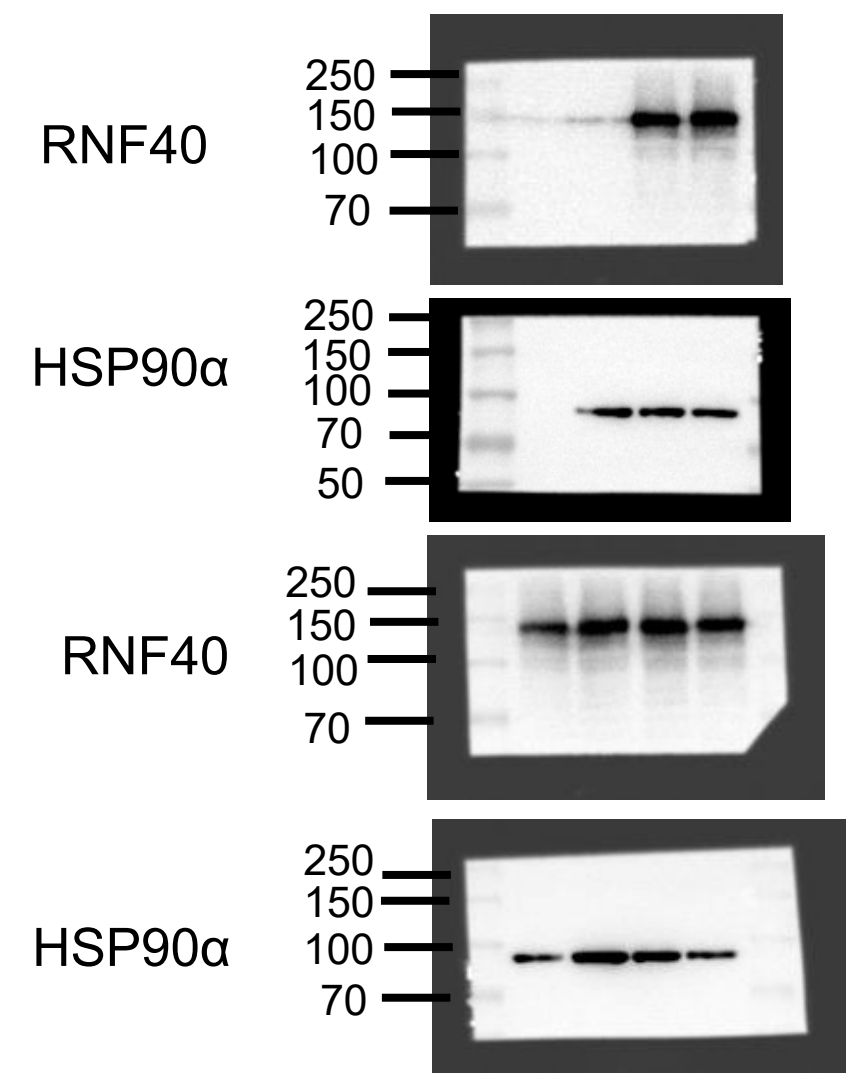

Figure4Q

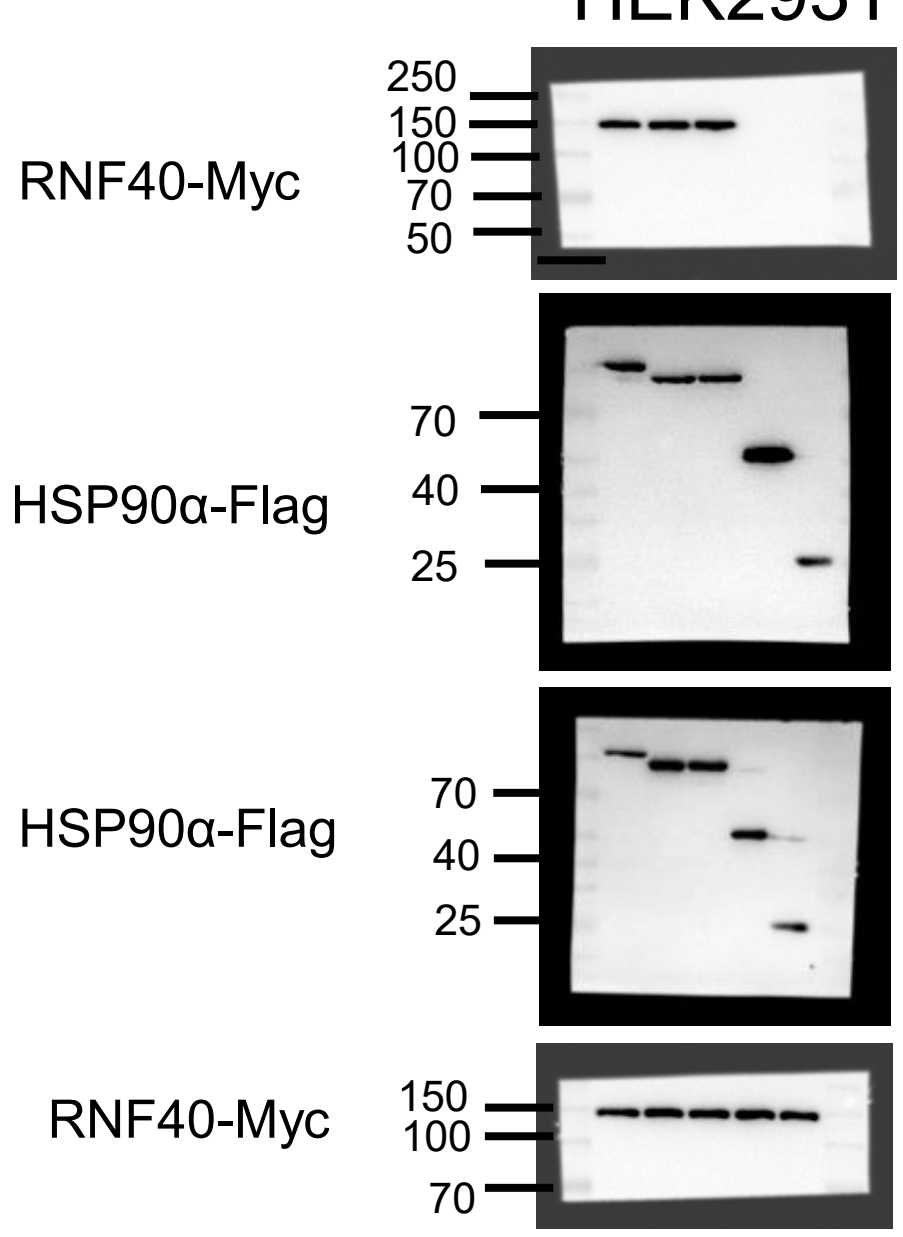

Figure4R

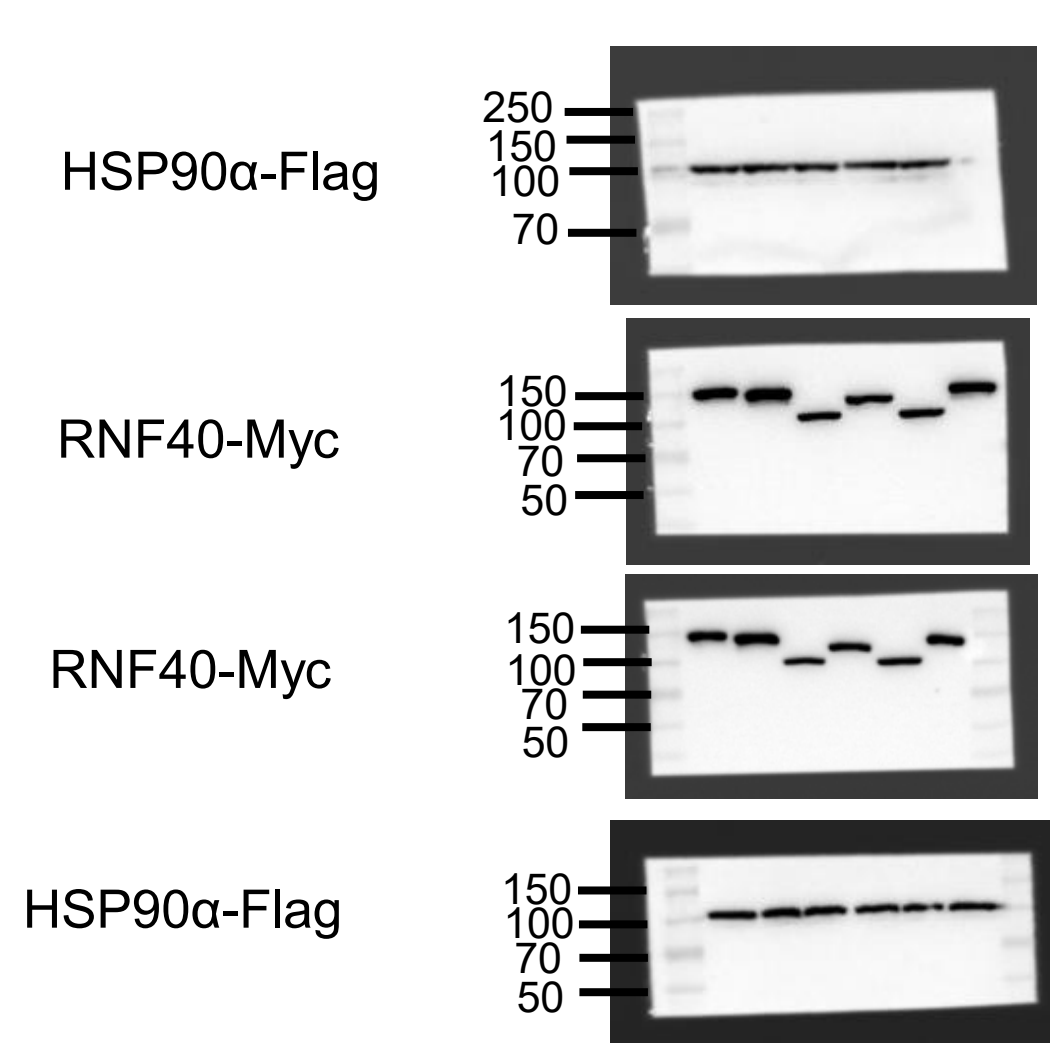

Figure5A

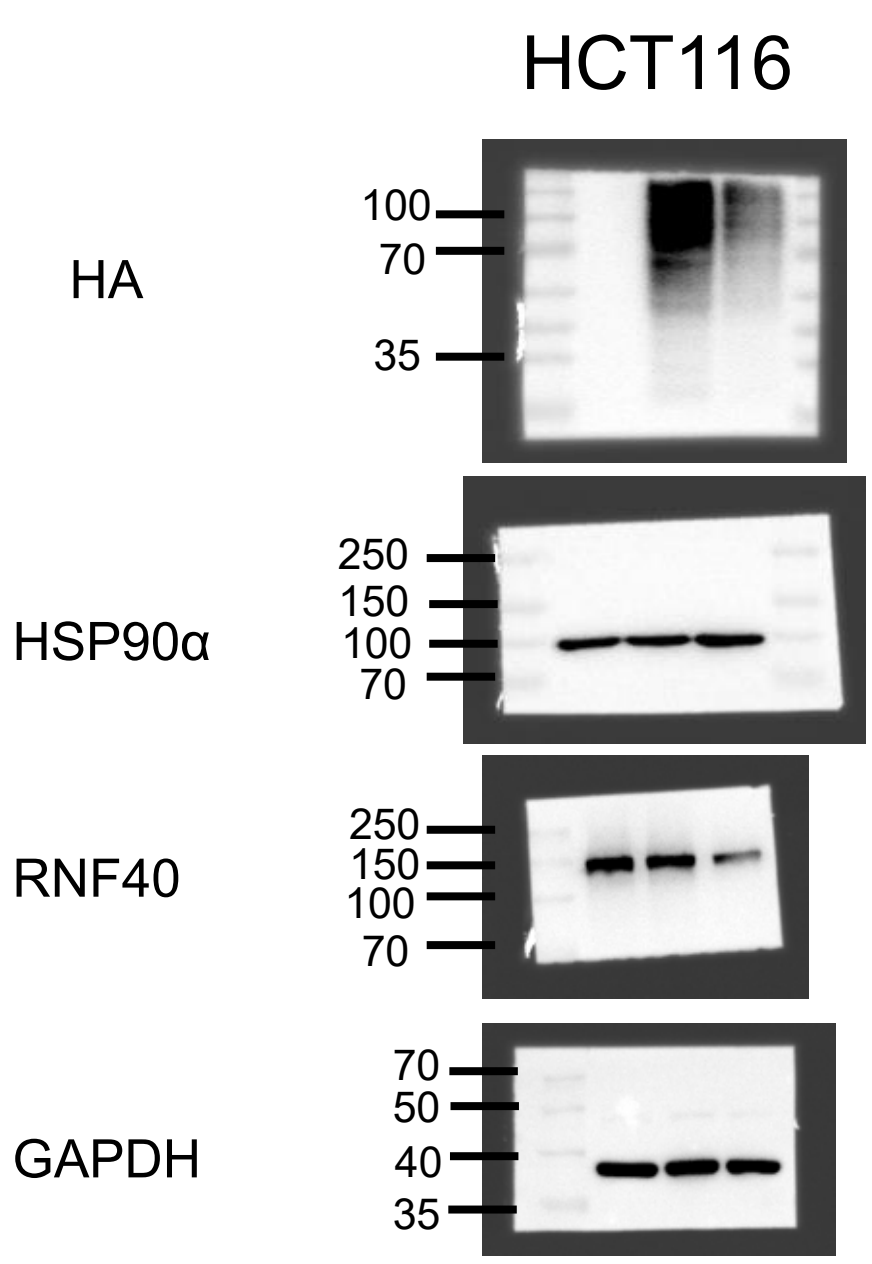

Figure5B

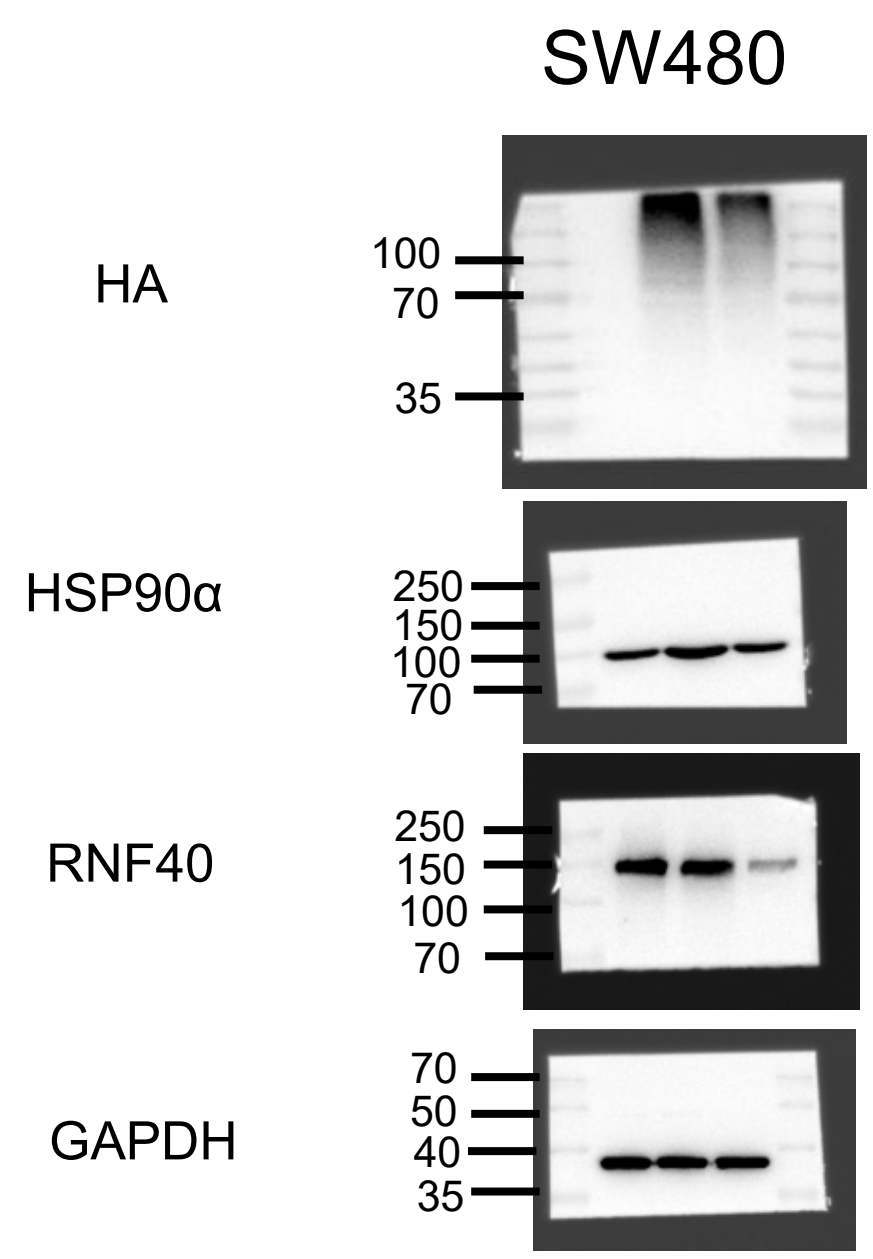

Figure5C

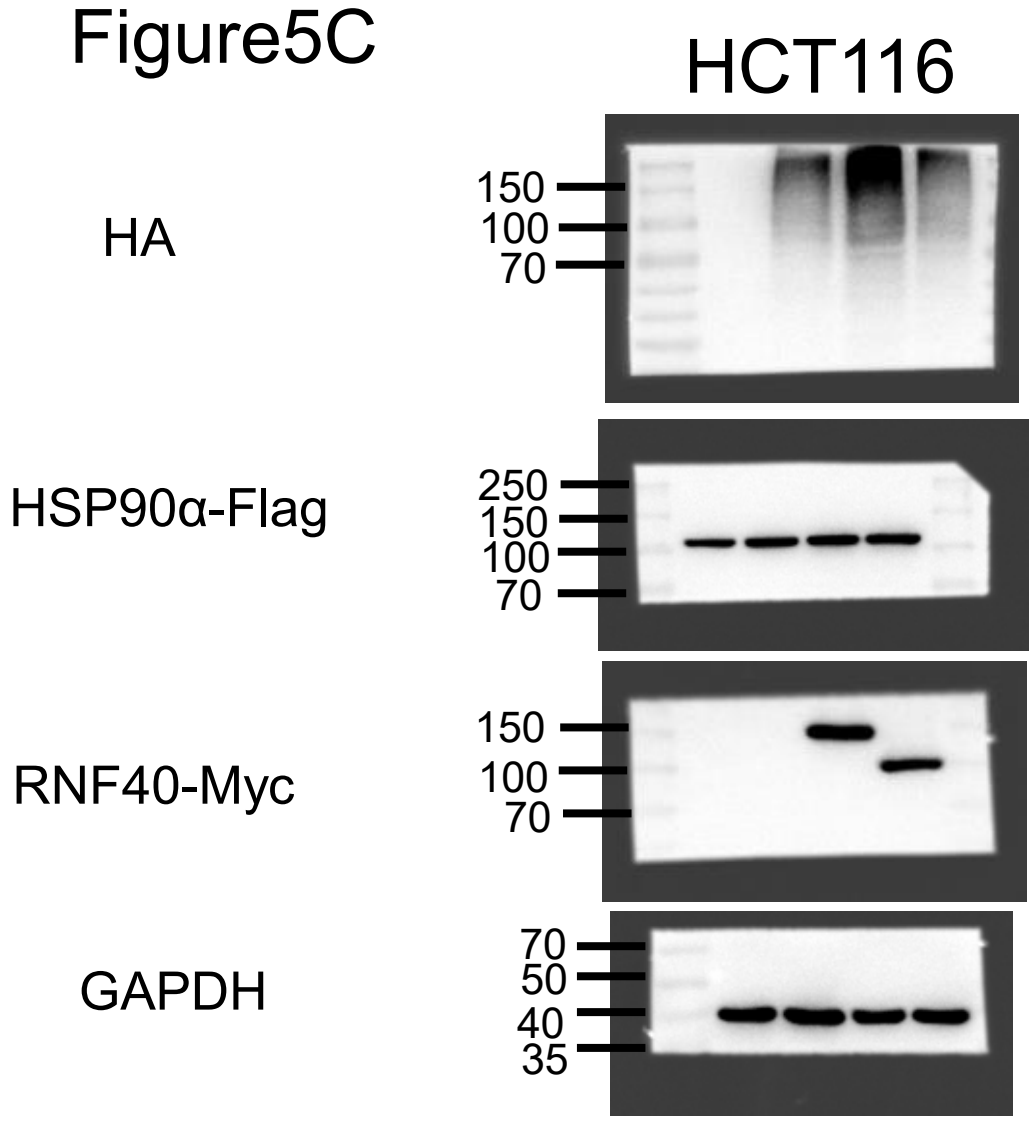

Figure5D

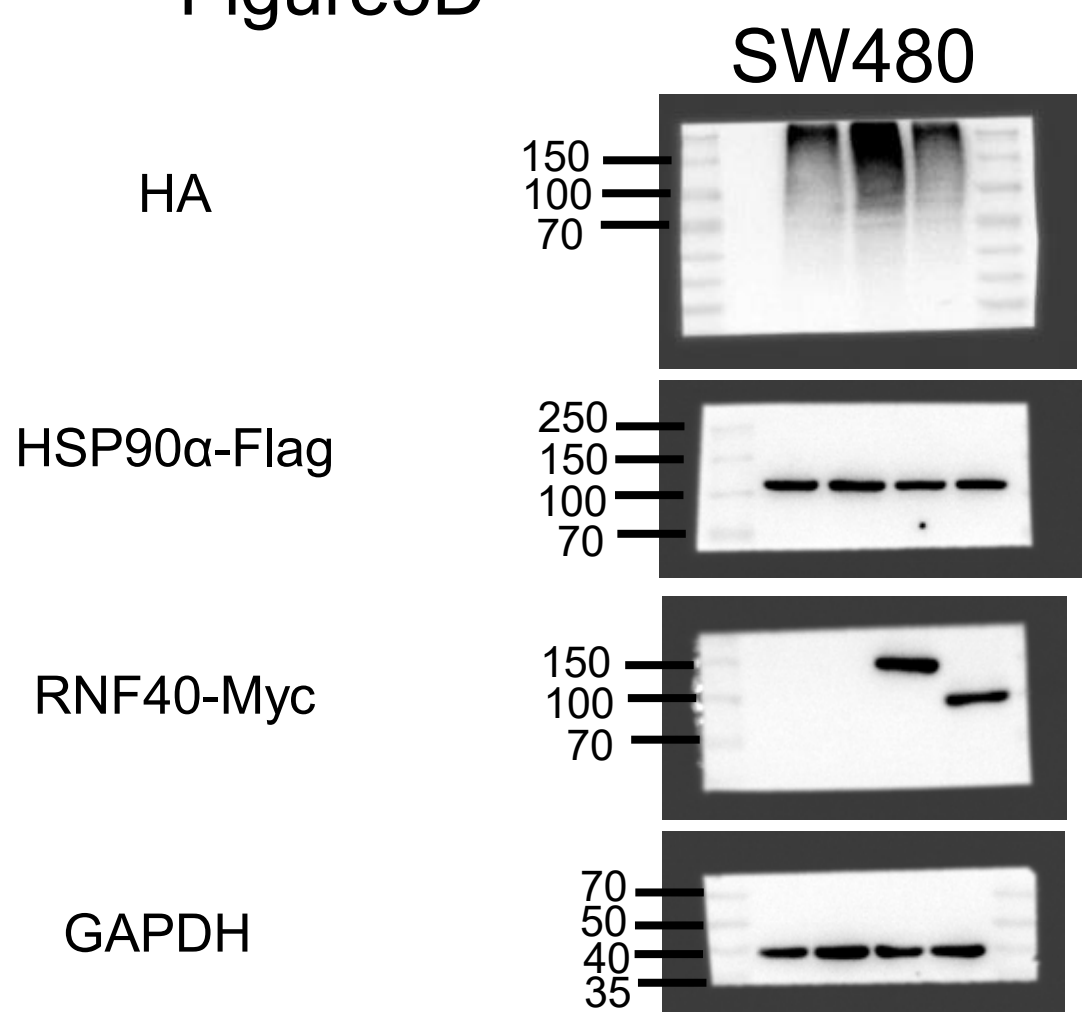

Figure5E

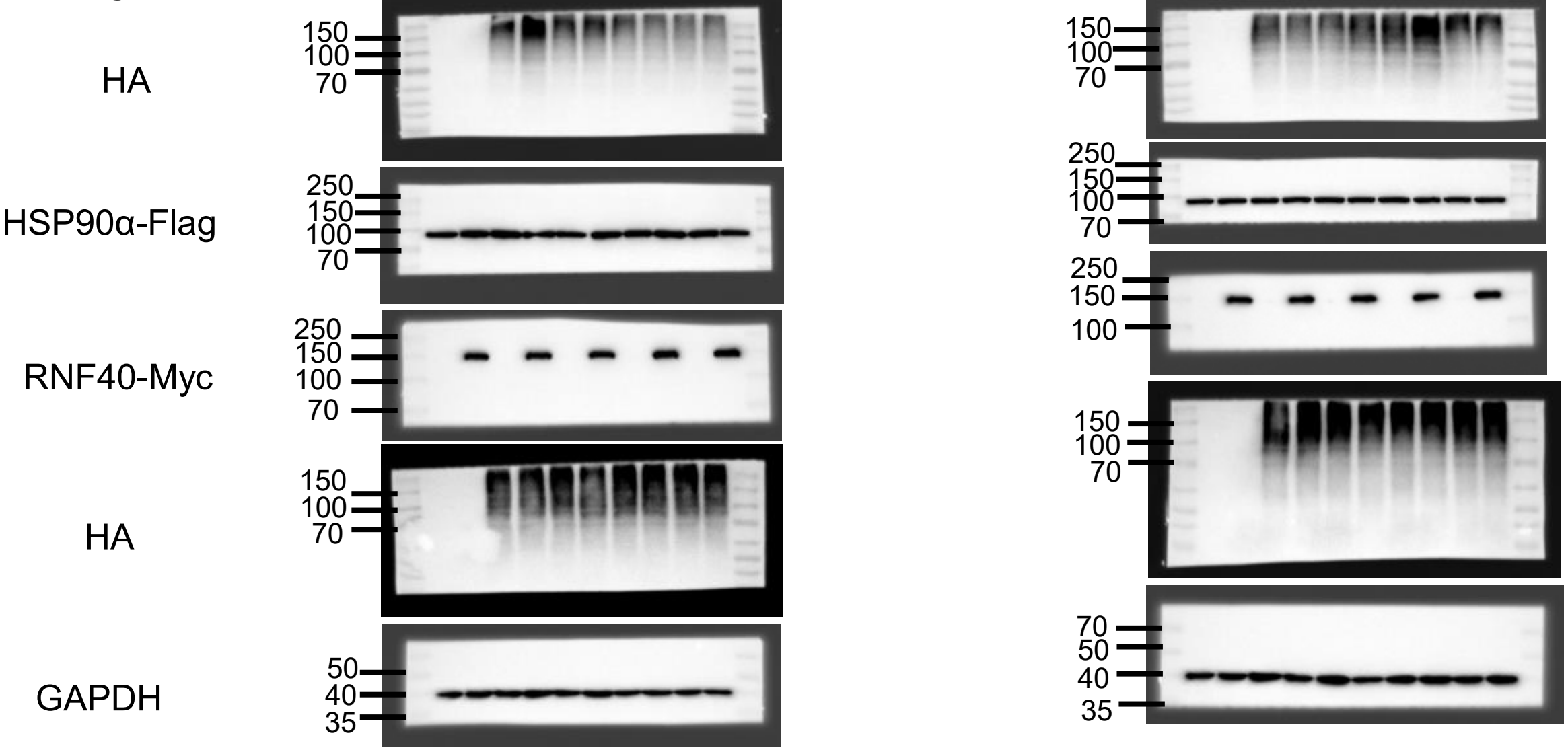

Figure5F

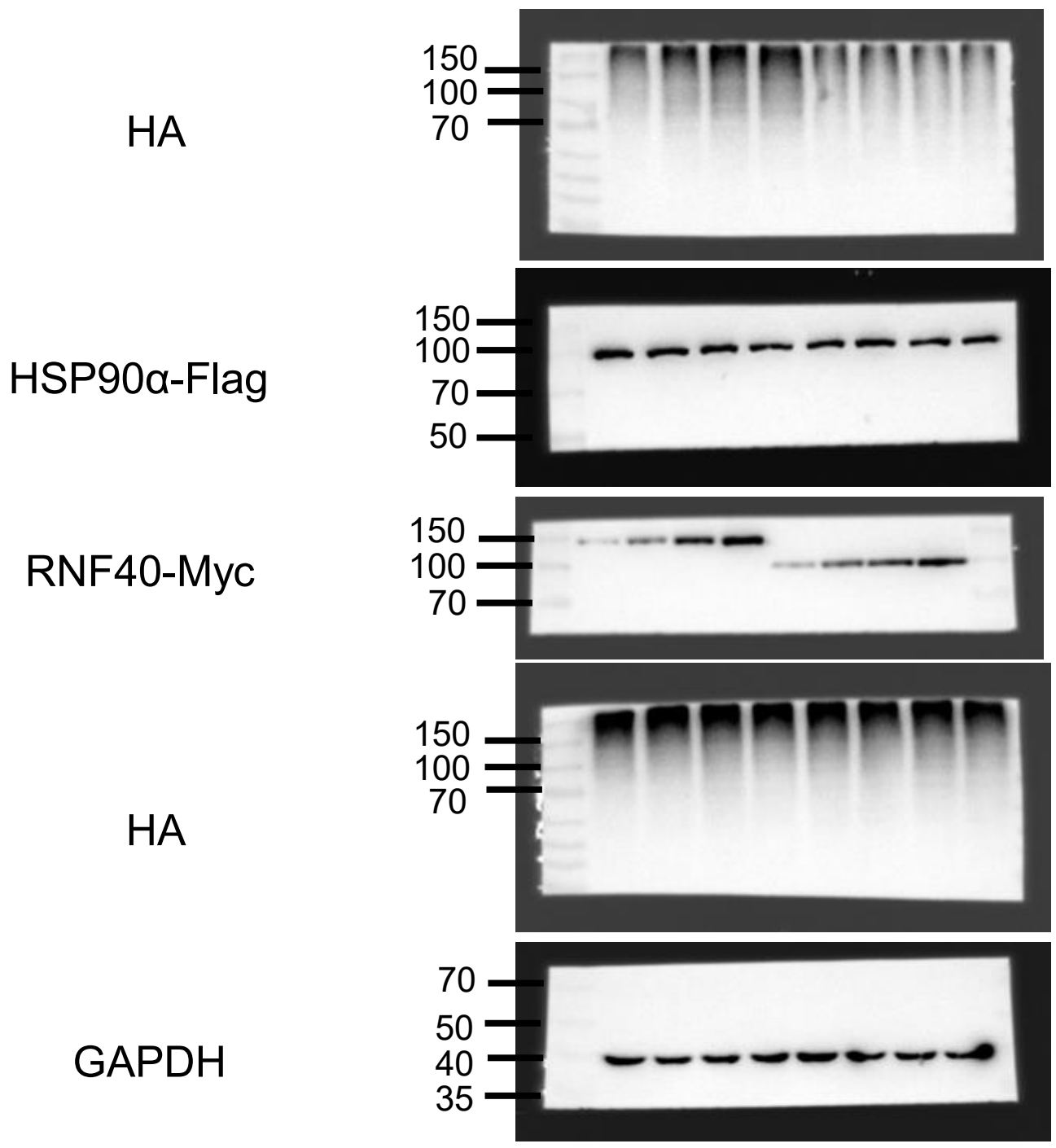

Figure5G

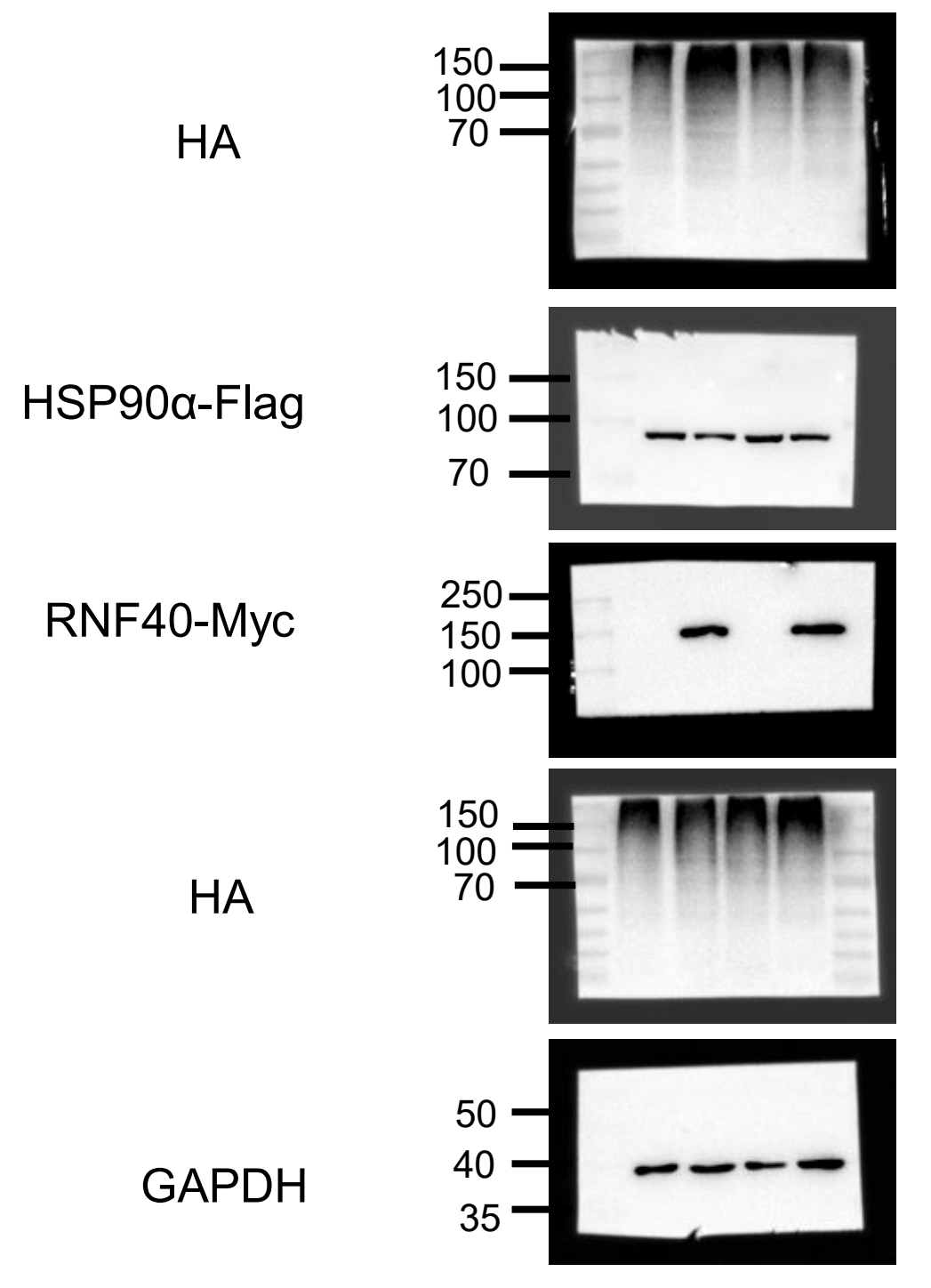

Figure5H

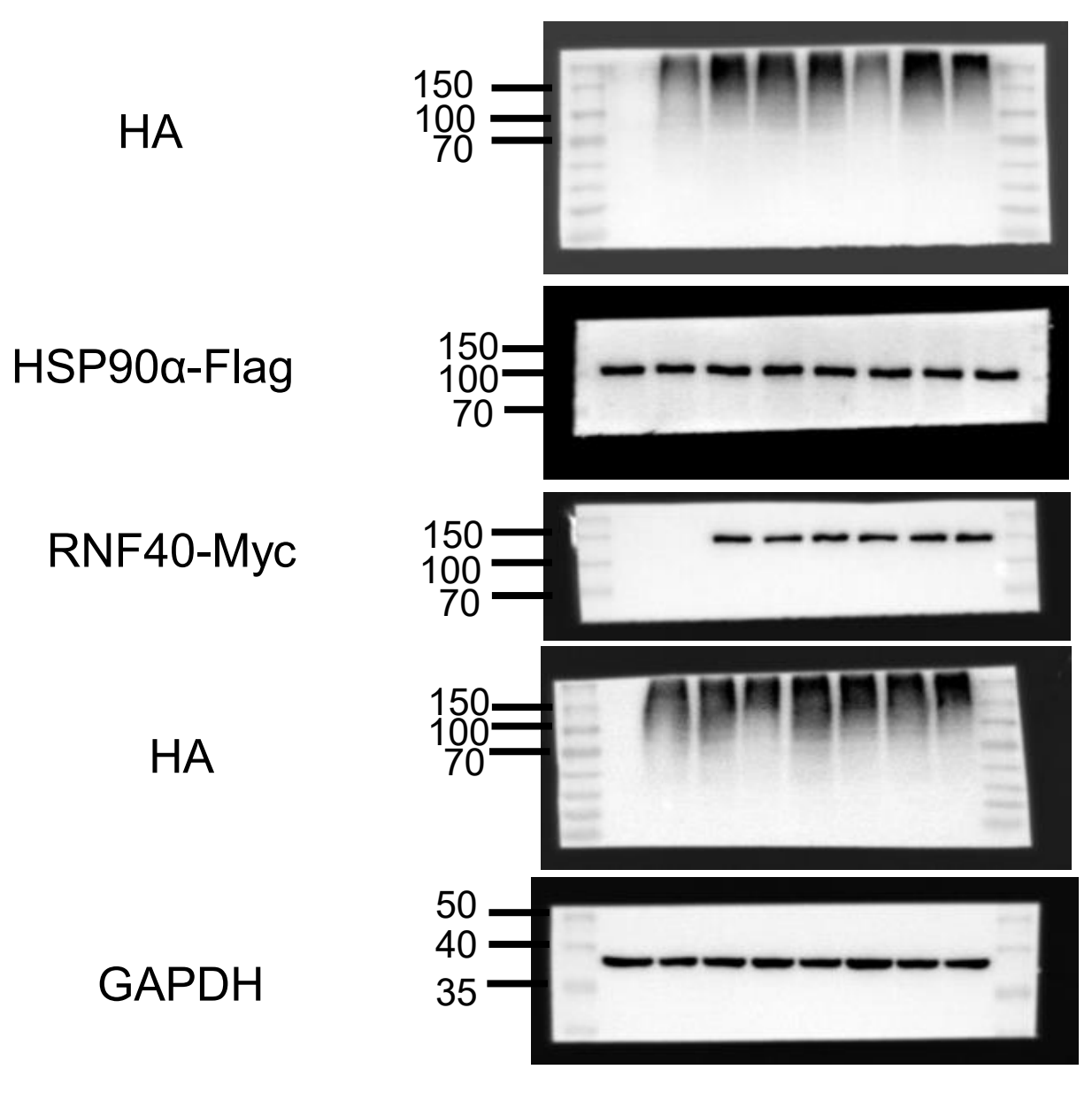

Figure5I

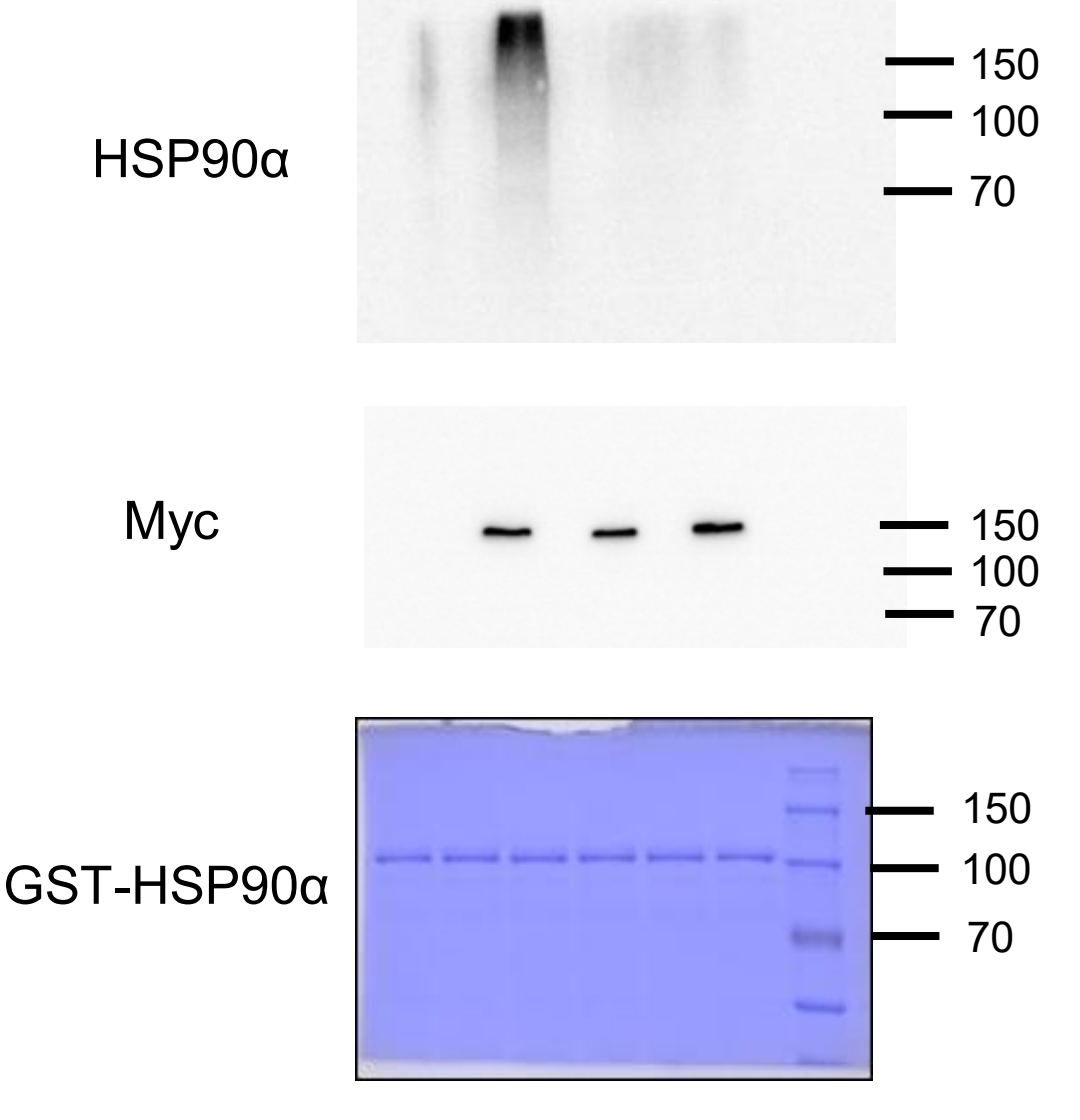

FigureS4B

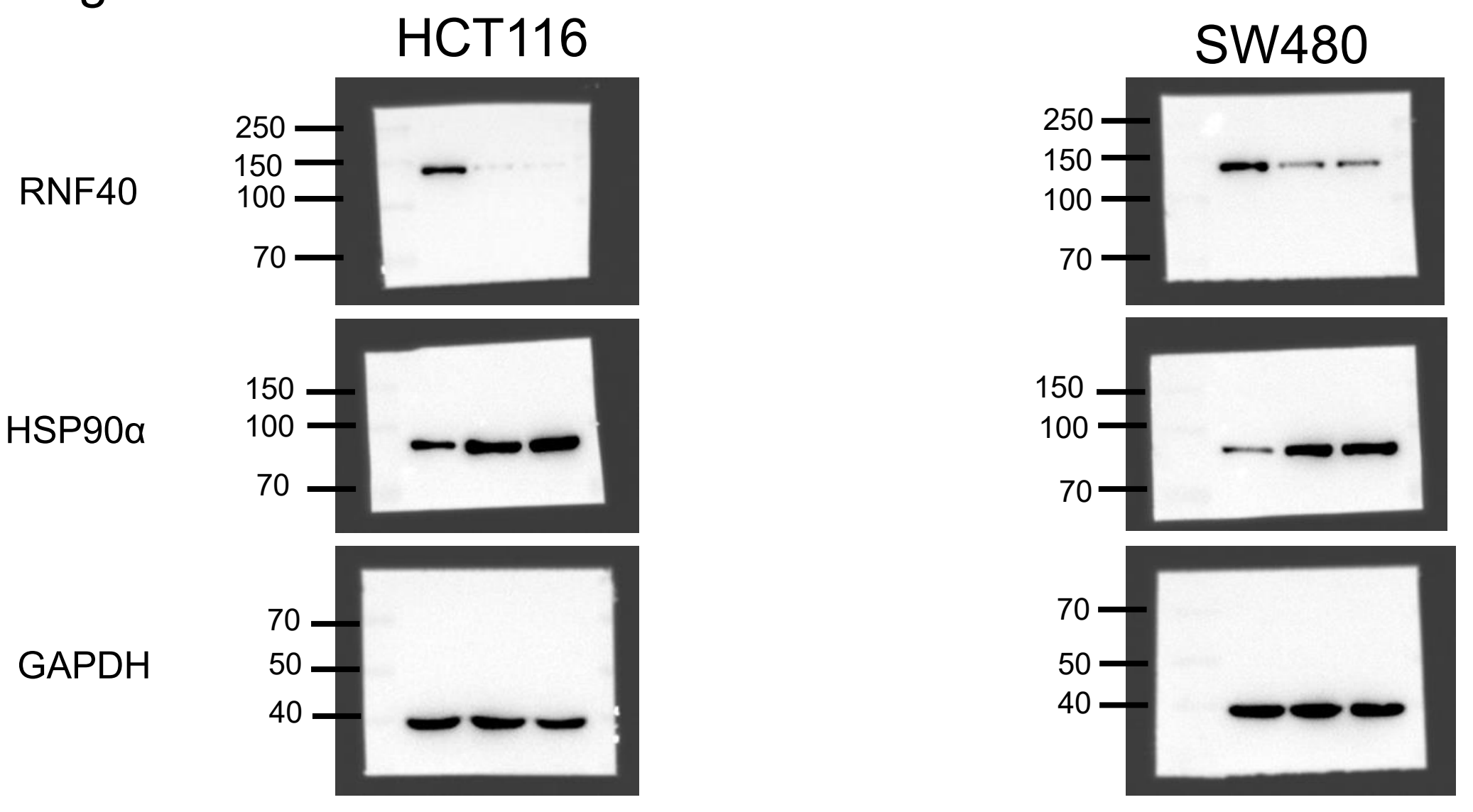

FigureS4C

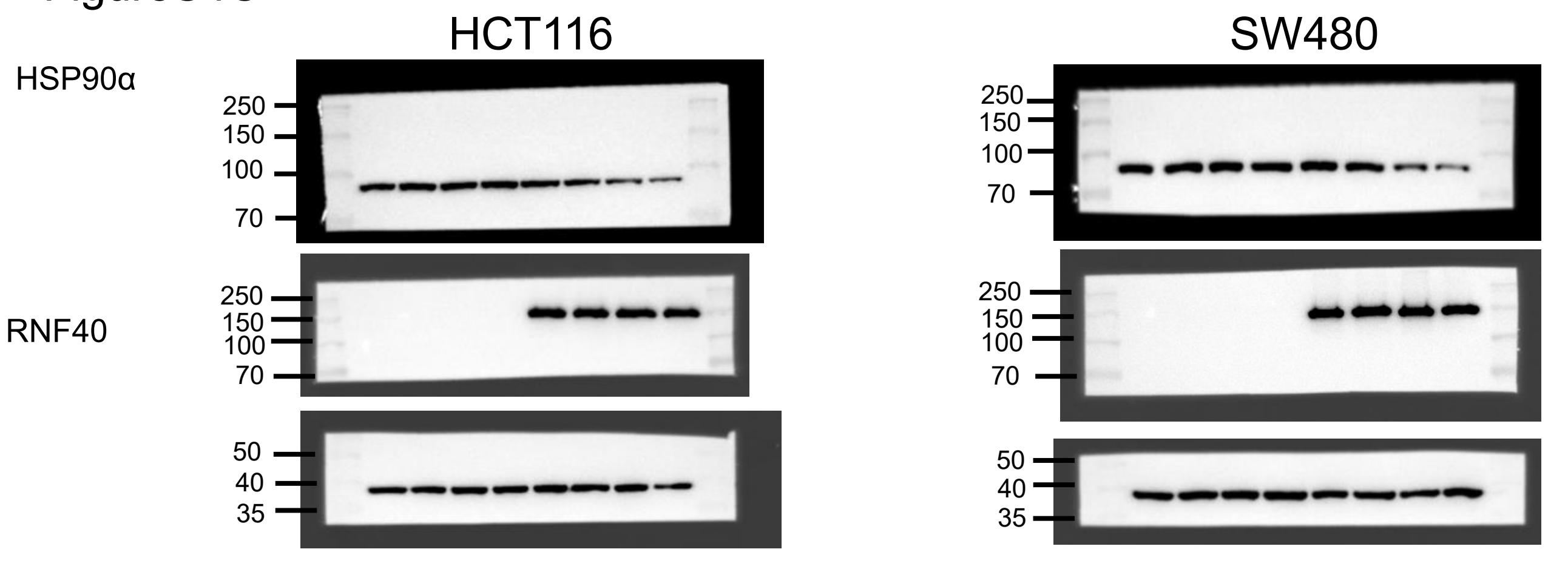

FigureS5A

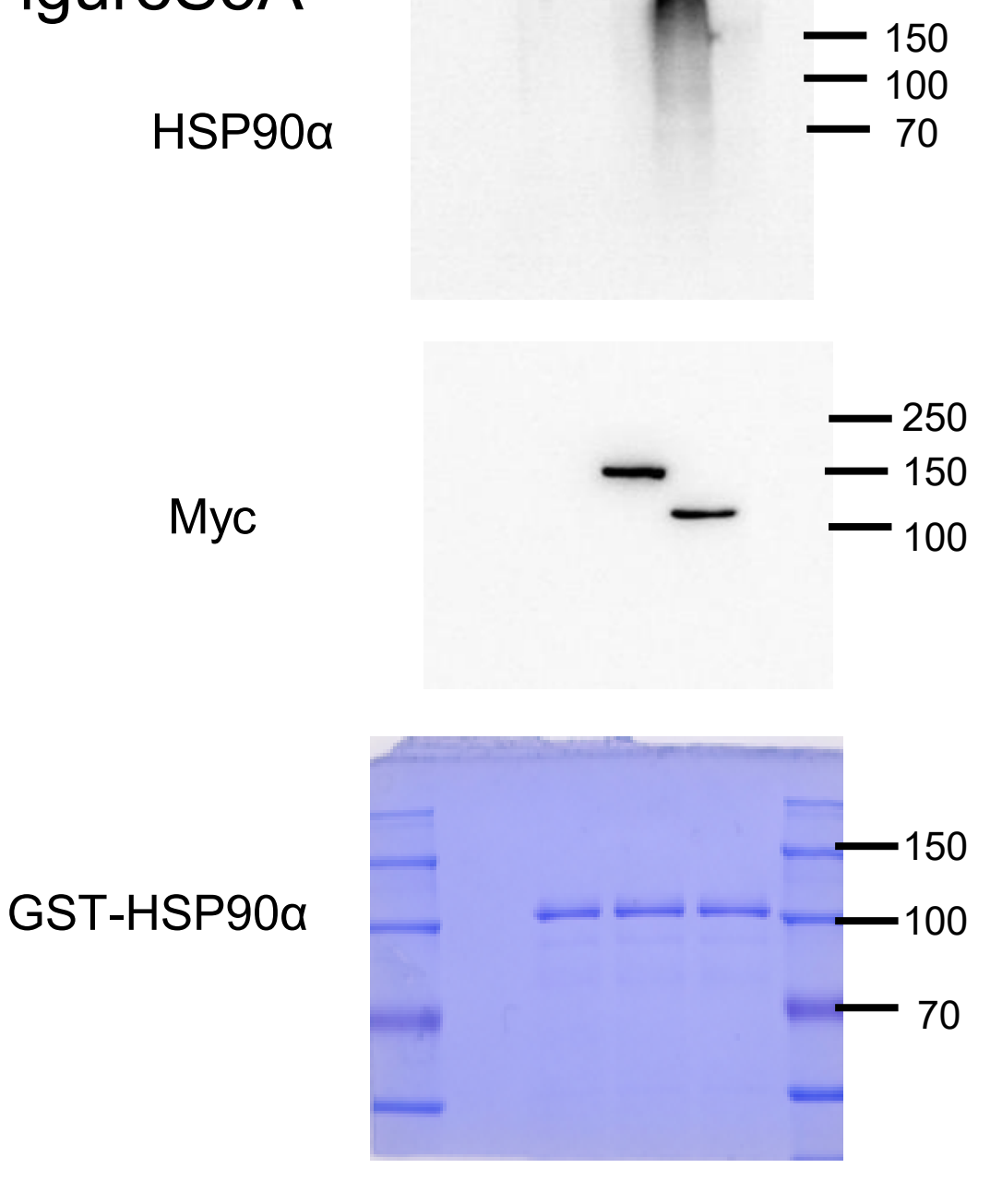

FigureS5D

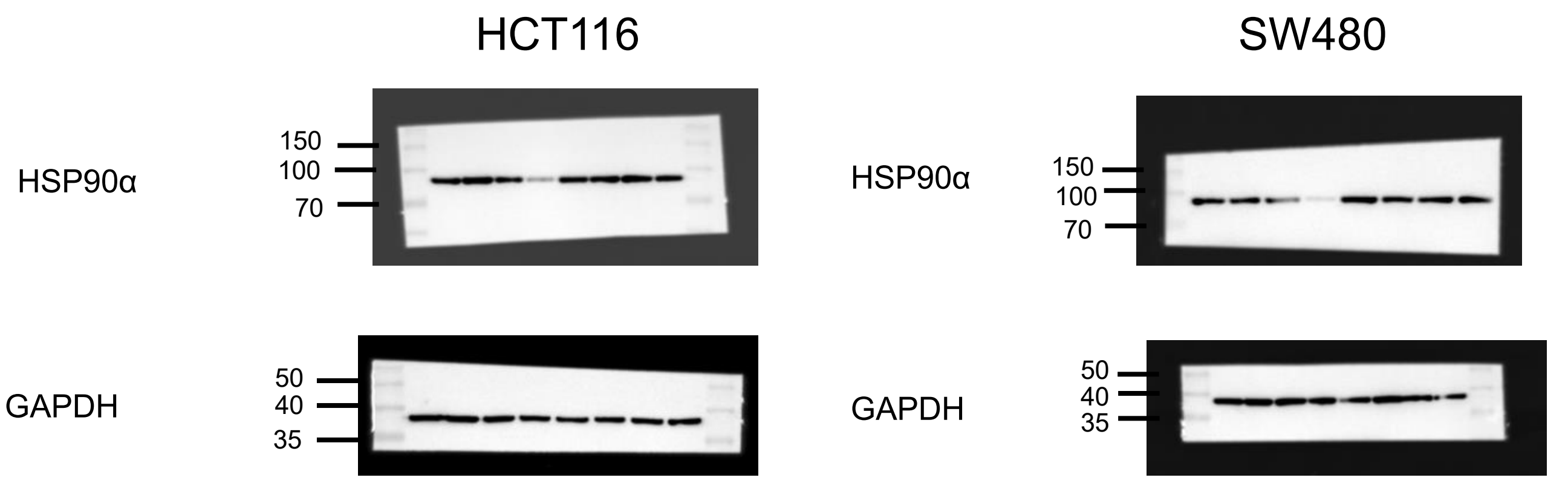
HCT116
SW480
HSP90α
150
100
70
GAPDH
50
40
35
HSP90α
150
100
70
GAPDH
50
40
35
