# Supplementary material for: Levistilide A promotes ferroptosis through the RNF40-HSP90α axis and inhibit colorectal cancer lung metastasis
Source: Cell Death Discov. 2026 Aug 15;12:453. doi: 10.1038/s41420-026-03265-x (PMC13754910; doi:10.1038/s41420-026-03265-x)
Supplement: Supplementary file 2 [file 41420_2026_3265_MOESM1_ESM.docx]

**FigureS1. Levistilide A suppresses colorectal cancer cells without causing damage to animal organs.**

(A-B)CCK-8 assay was performed to determine the half-maximal inhibitory concentration (IC₅₀) of LeA in SW480 cells after 48h or 72h treatment with various concentrations of the compound.

(C-D)CCK-8 assay was performed to determine the IC₅₀ of LeA in HCT116 cells after 48h or 72h treatment with various concentrations of the compound.

(E-G) CCK-8 assay was performed to determine the IC₅₀ of LeA in HCT8 cells after 24h, 48h or 72h treatment with various concentrations of the compound.

(H-J) CCK-8 assay was performed to determine the IC₅₀ of LeA in MC38 cells after 24h, 48h or 72h treatment with various concentrations of the compound.

(K-L) HE staining showing the morphology of the heart, liver, spleen, and kidneys in the mouse subcutaneous xenograft model (Left) or mouse lung metastasis model.

**FigureS2. Levistilide A–induced ferroptosis is associated with potential regulatory factors.**

(A) GSEA enrichment analysis showing the signaling pathways enriched in colorectal cancer cells upon LeA treatment. The top five ranked pathways are displayed, with the red arrow indicating the ferroptosis pathway.

(B) HCT116 cells were treated with the 17 compounds identified from the primary screen, with ferrostatin-1 (Fer-1, 10 μM) added 1 hour prior to drug treatment. After 24 hours of incubation, cell viability was measured using the CCK-8 assay. The y-axis represents relative cell viability. LeA ranked as the most potent compound.

(C) GSH/GSSG ratio in HCT116 and SW480 cells treated with DMSO, Fer-1 (5μM)，LeA (20 or 40 μM), or LeA (20 or 40 μM) plus Fer-1 (5 μM) for 12 hours. Data are presented as mean ± SEM from three independent experiments. **p < 0.01, ***p < 0.001, ****p < 0.0001; ns, not significant; one-way ANOVA with Tukey's multiple comparisons test.

(D) Intracellular Fe²⁺ levels measured by FerroOrange staining in HCT116 and SW480 cells treated with DMSO, Fer-1 (5μM)，LeA (20 or 40 μM), or LeA (20 or 40 μM) plus Fer-1 (5 μM) for 16 hours. Data are presented as mean ± SEM from three independent experiments. **p < 0.01, ***p < 0.001, ****p < 0.0001; ns, not significant; one-way ANOVA with Tukey's multiple comparisons test.

(E-F) A heatmap illustrating the changes in the expression of ferroptosis-related proteins in response to treatment with increasing concentrations of LeA (5, 10, 20, 40, and 80 μM) for 12h.

**FigureS3. HSP90α, the molecular target of levistilide A, functions as a novel suppressor of ferroptosis.**

(A) Molecular dynamics analysis illustrates the 3D free energy landscape of LeA binding to HSP90α, with radius of gyration (Rg) and root-mean-square deviation (RMSD) as the axes, representing the free energy distribution of the system in conformational space. The dark blue regions indicate the global free energy minimum, corresponding to the most thermodynamically stable conformations.

(B) The root-mean-square deviation (RMSD) is a reliable metric for assessing the conformational stability of proteins and ligands, reflecting the deviation of atomic positions from their initial coordinates. Smaller RMSD values indicate greater conformational stability. As shown, the complex system reaches equilibrium after 30 ns, indicating that this small molecule exhibits high stability when bound to its target protein.

(C) Root-mean-square fluctuation (RMSF) reflects the flexibility of amino acid residues within a protein. As shown, the RMSF values of this complex are relatively low, indicating reduced flexibility and consequently enhanced structural stability.

(D) RT–qPCR analysis showed that treatment with low (LeA-L, 20 μM) or high (LeA-H, 40 μM) concentrations of LeA did not significantly affect HSP90AA1 mRNA expression in HCT116 and SW480 cells.

(E) CTRP database analysis demonstrated that, in intestinal tissues, HSP90AA1 expression is significantly associated with sensitivity to multiple ferroptosis inducers, including ML162, ML210, 1S,3R-RSL3, and erastin.

(F) DepMap database analysis showed that HSP90AA1 expression exhibited no significant correlation with the knockdown of FSP1, GPX4, SLC7A11, or DHODH.

(G) In the HCT116 and SW480 cell lines, knockdown of HSP90AA1 increased cellular sensitivity to the ferroptosis inducer RSL3.

(H) In the HCT116 and SW480 cell lines, knockdown of HSP90AA1 increased cellular sensitivity to the ferroptosis inducer Erastin.

**FigureS4. RNF40 downregulates HSP90α protein expression.**

(A) TCGA database showing a weak correlation between HSP90AA1 and RNF40 (R = 0.19, *P* = 0.00079).
(B) GSE21815 dataset (n=141) validating the positive correlation (R = 0.5771, *P* < 0.001).
(C) GSE31905 dataset (n=67) further validating the positive correlation (R = 0.3485, *P* < 0.0039).

(D) Knockdown of RNF40 increases HSP90α expression in HCT116 and SW480 cells.

(E) Overexpression of RNF40-Myc significantly decreases the protein half-life of HSP90α in HCT116 and SW480 cells treated with CHX (100 μg/ml) for 0, 6, 12, and 24 hours.

(F) Representative SPR sensograms and steady-state fitting for binding of LeA (0.15625 – 20 μM) to immobilized RNF40. The equilibrium dissociation constant (K_D_) was calculated as 0.853 μM.

(G) RNF40 was pre-incubated with LeA (10 μM) for 1 hour, and the complex was injected over a CM5 chip immobilized with HSP90α. The resulting binding curve yielded a K_D_ of 0.29 μM, representing an approximately 2.5- to 3-fold higher affinity compared to the binary interactions (LeA-HSP90α: 0.74 μM, LeA-RNF40: 0.853 μM).

**FigureS5. RNF40 catalyzes the ubiquitination of HSP90α at K407**

(A) 50 µg of purified GST-HSP90α protein was incubated at 4 °C with HEK293T crude lysates transfected with HA-Ub, RNF40-Myc, or RNF40-Myc mutation plasmids. After incubation, proteins were eluted, mixed with 2× SDS loading buffer, boiled, and subjected to WB analysis.

(B) Schematic representation of the modifiable sites on HSP90α protein. Ubiquitination sites with References (HTP + LTP) scores greater than 30 were selected, including K292, K362, K407, K443, and K615.

(C) Conservation of the K407 residue across multiple species.

(D-E)Overexpression of HSP90α-Flag K407R significantly decreases the protein half-life of HSP90α in HCT116 and SW480 cells treated with CHX (100 μg/ml) for 0, 6, 12, and 24 hours.

**FigureS6. LeA affects cellular MDA levels through RNF40-HSP90α axis**

(A) HCT116 or SW480 cells stably transfected with shRNF40 or HSP90α were treated with 40 µM LeA or DMSO for 16 hours, Subsequently, MDA levels were measured using MDA assay kit.

(B) HCT116 or SW480 cells stably transfected with RNF40 or shHSP90α were treated with 40 µM LeA or DMSO for 16 hours. Subsequently, MDA levels were measured using MDA assay kit.

**FigureS7. The tissue microarray showing HSP90α staining in colorectal cancer samples.**

(A) The tissue microarray showing HSP90α staining in colorectal cancer samples.
