# Supplementary material for: Levistilide A promotes ferroptosis through the RNF40-HSP90α axis and inhibit colorectal cancer lung metastasis
Source: Cell Death Discov. 2026 Aug 15;12:453. doi: 10.1038/s41420-026-03265-x (PMC13754910; doi:10.1038/s41420-026-03265-x)
Supplement: Supplementary file 3 [file 41420_2026_3265_MOESM2_ESM.docx]

**Table S1. The shRNA primers in this study.**

| **shRNA** |  | **Primers (5’-3’)** |
| --- | --- | --- |
| HSP90AA1-1 | Forward | CCGGGAAGCAGGGCACCTGTTAACTCTCGAGAGTTAACAGGTGCCCTGCTTCTTTTTG |
|  | Reverse | AATTCAAAAAGAAGCAGGGCACCTGTTAACTCTCGAGAGTTAACAGGTGCCCTGCTTC |
| HSP90AA1-2 | Forward | CCGGGCAGCCATTTATATTGCTTAGCTCGAGCTAAGCAATATAAATGGCTGCTTTTTG |
|  | Reverse | AATTCAAAAAGCAGCCATTTATATTGCTTAGCTCGAGCTAAGCAATATAAATGGCTGC |
| RNF40-1 | Forward | CCGGGCACCTGGCAGAGGCCTTAGACTCGAGTCTAAGGCCTCTGCCAGGTGCTTTTTG |
|  | Reverse | AATTCAAAAAGCACCTGGCAGAGGCCTTAGACTCGAGTCTAAGGCCTCTGCCAGGTGC |
| RNF40-2 | Forward | CCGGGCTTAACTCTGGCTACTATGTCTCGAGACATAGTAGCCAGAGTTAAGCTTTTTG |
|  | Reverse | AATTCAAAAAGCTTAACTCTGGCTACTATGTCTCGAGACATAGTAGCCAGAGTTAAGC |
| Hsp90aa1-1 | Forward | CCGGGGAAGGAGCTGCACATCAATCCTCGAGGATTGATGTGCAGCTCCTTCCTTTTTG |
|  | Reverse | AATTCAAAAAGGAAGGAGCTGCACATCAATCCTCGAGGATTGATGTGCAGCTCCTTCC |
| Hsp90aa1-2 | Forward | CCGGGCTGCACATCAATCTCATTCCCTCGAGGGAATGAGATTGATGTGCAGCTTTTTG |
|  | Reverse | AATTCAAAAAGCTGCACATCAATCTCATTCCCTCGAGGGAATGAGATTGATGTGCAGC |

**Table S2. Primers for HSP90AA1 mutation sites.**

| **sites** |  | **Primers (5’-3’)** |
| --- | --- | --- |
| K292R | Forward | GCTCAACAGAACAAAGCCCATCTGGACCAGA |
|  | Reverse | GCTTTGTTCTGTTGAGCTCTTCTTGATCGATGTACT |
| K362R | Forward | CAGAACTTGTATGTACGCAGAGTTTTCATCA |
|  | Reverse | CGTACATACAAGTTCTGATGTTGTTCTTTTTCTTTCTGTTTTC |
| K407R | Forward | GTTGCAACAAAGCAGAATTTTGAAAGTTATCAGGAAGAATTTGG |
|  | Reverse | TTCTGCTTTGTTGCAACATCTCACGG |
| K443R | Forward | GCAGTTCTCTAGAAACATAAAGCTTGGAATACACGAAGA |
|  | Reverse | TGTTTCTAGAGAACTGCTCATAGAATTTCTTGTAGT |
| K615R | Forward | ATCATGAGAGCTCAAGCCCTAAGAGACAACTCA |
|  | Reverse | GCTTGAGCTCTCATGATTCTCTCCATGTTTGCTGT |

**Table S3. Information on the antibodies**.

| **Antibodies** | **Brand** | **Catalog Number** | **Concentration** | **Application** |
| --- | --- | --- | --- | --- |
| Mouse anti-Flag | Abclonal | AE005 | 2ug | IP |
| Rabbit anti-Myc | Abclonal | AE070 | 2ug | IP |
| Rabbit anti-HA | Abclonal | AE105 | 2ug | IP |
| Mouse anti-Flag | Abclonal | AE005 | 1:20000 | WB |
| Rabbit anti-Myc | Abclonal | AE070 | 1:50000 | WB |
| Rabbit anti-HA | Abclonal | AE105 | 1:20000 | WB |
| Rabbit anti-ACSL4 | Abclonal | A20414 | 1:10000 | WB |
| Rabbit anti-GPX4 | ZEN-BIOSCIENCE | R381958 | 1:2000 | WB |
| Rabbit anti-HSP90α | ZEN-BIOSCIENCE | R380809 | 1:2000 | WB |
| Rabbit anti-xCT | ZEN-BIOSCIENCE | R382036 | 1:2000 | WB |
| Rabbit anti-FSP1 | ZEN-BIOSCIENCE | 342551 | 1:2000 | WB |
| Rabbit anti-TFR1 | ZEN-BIOSCIENCE | R381603 | 1:2000 | WB |
| Rabbit anti-FTH1 | ZEN-BIOSCIENCE | R23306 | 1:2000 | WB |
| Rabbit anti-SLC3A2 | ZEN-BIOSCIENCE | R383101 | 1:2000 | WB |
| Mouse anti-GAPDH | ZEN-BIOSCIENCE | R380626 | 1:20000 | WB |
| Goat anti-rabbit | ZEN-BIOSCIENCE | 511203 | 1:10000 | WB |
| Goat anti-mouse | ZEN-BIOSCIENCE | 511103 | 1:10000 | WB |
| Mouse anti-RNF40 | Santa cruz | sc-293333 | 1:50 | IF |
| Rabbit anti-HSP90α | ZEN-BIOSCIENCE | R380809 | 1:100 | IF |
| Goat Anti-Rabbit 488 | Proteintech | SA00013-02 | 1:200 | IF |
| Donkey Anti-Mouse 594 | Proteintech | SA00013-07 | 1:200 | IF |
| CD8 | Abclonal | A11856 | 1:200 | IHC |
| GZMB | Abclonal | A2557 | 1:200 | IHC |
